# Design and synthesis of cabotegravir derivatives bearing 1,2,3-triazole and evaluation of anti-liver cancer activity

**DOI:** 10.3389/fphar.2023.1265289

**Published:** 2023-10-06

**Authors:** Huaxia Xie, Longfei Mao, Gaolu Fan, Ziyuan Wu, Yimian Wang, Xixi Hou, Jiangang Wang, Huili Wang, Ling Liu, Sanqiang Li

**Affiliations:** ^1^ College of Basic Medicine and Forensic Medicine, Henan University of Science and Technology, Luoyang, China; ^2^ Department of Pharmacy, Luoyang Third People’s Hospital, Luoyang, China; ^3^ Department of Pharmacy, The First Affiliated Hospital, College of Clinical Medicine of Henan University of Science and Technology, Luoyang, China; ^4^ University of North Carolina Hospitals, Chapel Hill, NC, United States

**Keywords:** cabotegravir, 1,2,3-triazole, derivatives, apoptosis, invasion

## Abstract

Based on the structure of the anti-HIV drug cabotegravir, we introduced 1,2,3-triazole groups with different substituents to obtain 19 cabotegravir derivatives and tested their activity against HepG2 cells. The proliferation of HepG2 cells was examined following treatment with derivatives. Most of the compounds demonstrated significant inhibitory effects, particularly compounds KJ-5 and KJ-12 with IC_50_ values of 4.29 ± 0.10 and 4.07 ± 0.09 μM, respectively. Furthermore, both compounds 5 and 12 significantly caused cell apoptosis, G2/M arrest, and DNA damage, and suppressed invasion and migration in a concentration-dependent manner. In addition, KJ-5 and KJ-12 could trigger apoptosis via the mitochondrial pathway by increasing the ratio of Bax/Bcl-2 and activating cleaved caspase-9, cleaved caspase-3, and cleaved PARP.

## 1 Introduction

**FIGURE 1:**
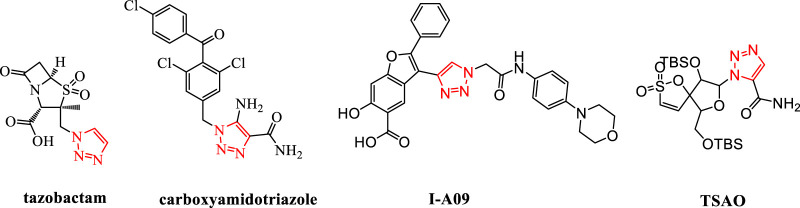
Structures of tazobactam, carboxyamidotriazole, I-A09, and TSAO.

1,2,3-Triazole is an important nitrogen-containing five-membered heterocyclic group. It can act as a bioisostere for various groups such as amides, carboxylic acids, and esters (Wang B. et al., 2018). By replacing the corresponding structures in the original drug molecules, it can avoid related patent protection and is an important building block for drug molecules (Wu et al., 2017; Qi et al., 2020). The use of 1,2,3-triazole for structural modification has become an effective weapon for breaking through the patents of original research drugs and carrying out generic innovation. It has now appeared widely in marketed drugs, such as the marketed drug tazobactam; carboxyamidotriazole, which is being applied for listing; and I-A09 and TSAO containing the 1,2,3-triazole structure, which are undergoing clinical research (Mao L. et al., 2020; Thacker et al., 2020) (Figure 1).

**FIGURE 2:**
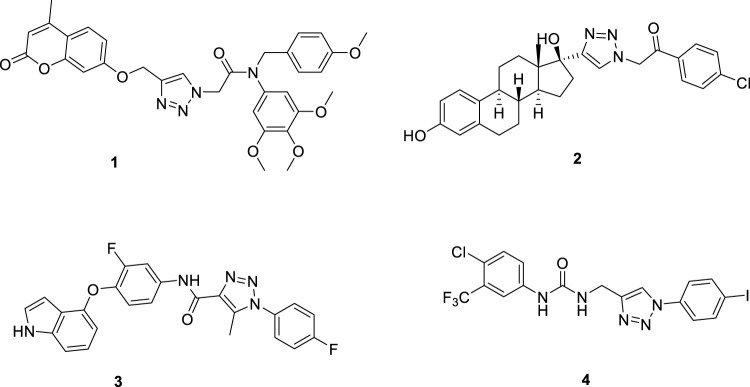
Structures of some 1,2,3-triazole derivatives.

Meanwhile, due to the planar rigid structure of 1,2,3-triazole, it can be inserted into the DNA of tumor cells, interact with DNA, and cause damage to the DNA of tumor cells. Therefore, this structure is widely used in the development of new anti-tumor drugs (Bertrand et al., 2015; Rohrig et al., 2019; Mao L. F. et al., 2020; Mao et al., 2022). For example, a research group synthesized a series of trimethoxyphenyl-1,2,3-triazole hybrids containing coumarin, among which compound **1** had a half-maximal inhibitory concentration (IC_50_) of 1.71 μM for the inhibition of HepG2 cells (Fu et al., 2019). A research group used ethynyl estradiol as the parent nucleus and obtained a series of compounds containing the 1,2,3-triazole structure through the click chemistry reaction, which had moderate inhibitory effects on HepG2 cells. Among them, compound **2** had an IC_50_ value of 17.8 μM for HepG2 cells (Queiroz et al., 2019). Another research group synthesized a series of pyrrolo [2,3-d] pyrimidine and pyrazolo[3,4-d] pyrimidine derivatives bearing 1,2,3-triazole, which had good inhibitory effects on HepG2 cells. Among them, compound **3** had an IC_50_ value of 2.03 μM for HepG2 cells and could inhibit apoptosis and cause cell cycle arrest at the G2/M phase (Wang L. et al., 2018; Chen et al., 2022b). A research group synthesized a new series of 1,2,3-triazole-cored structures incorporating aryl urea to test their inhibitory activity against HepG2 cells. They found that compound **4** had an IC_50_ value of 5.57 μM for HepG2 cells, which was comparable to the positive drug sofosbuvir (Figure 2). It could induce apoptosis in HepG2 cells and showed good affinity, making it a potential anti-liver cancer lead compound (Chen et al., 2022a; Oekchuae et al., 2022).

**FIGURE 3:**
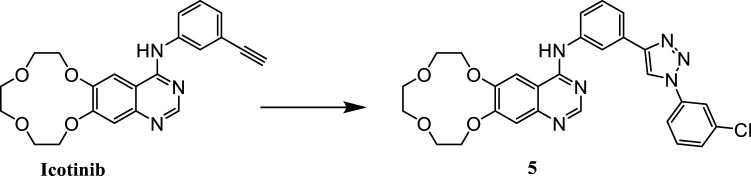
Structure of icotinib derivatives (5).

Our preliminary research was based on the structural modification of icotinib to obtain 1,2,3-triazole compounds for anti-tumor activity experiments (Mao L. F. et al., 2020). We found that derivatives with phenyl substituents were superior to those with benzyl substituents. Compound 5 is one of icotinib derivatives (Figure 3). Because the epidermal growth factor receptor (EGFR) inhibitor icotinib has a structural feature of a terminal alkyne, it reacted with 3-chlorophenyl azide to produce compound 5, which showed excellent inhibitory effects on mutant lung cancer cells (PC-9) and wild-type lung cancer cells (A549), and had a stronger effect than icotinib. Compound 5 downregulated the expression of caspase-3, causing fragmentation, pyknosis, and dense hyperchromasia of nuclei in A549 cells, inducing apoptosis and arresting A549 cells at the G2/M phase.

Cabotegravir is an inhibitor of HIV integrase, which means it blocks the HIV’s enzyme integrase, thereby preventing its genome from being integrated into the human cells’ DNA (Engelman and Engelman, 2021). Studies have found that in the later stages of HIV, the accumulation of drug toxicity due to long-term medication and low immunity can lead to the occurrence of liver cancer (Hu and Ludgate, 2007).

**FIGURE 4:**
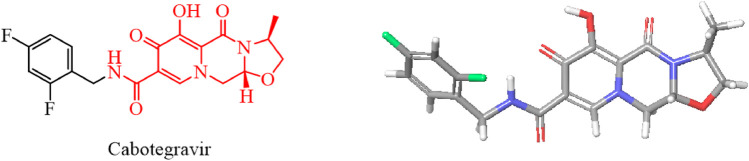
Energy minimization molecular structure of cabotegravir.

We used the rigid planar structure of cabotegravir as a lead compound for structural modification. After minimizing the energy of the cabotegravir structure, we found that the red part of its structure exhibits a planar structure. We utilized this feature to modify its structure by introducing the 1,2,3-triazole structure, which also has a rigid planar structure and can be inserted into DNA. We hope that the compound we obtain will show good inhibitory activity against tumor cells (Figure 4).

**FIGURE 5:**
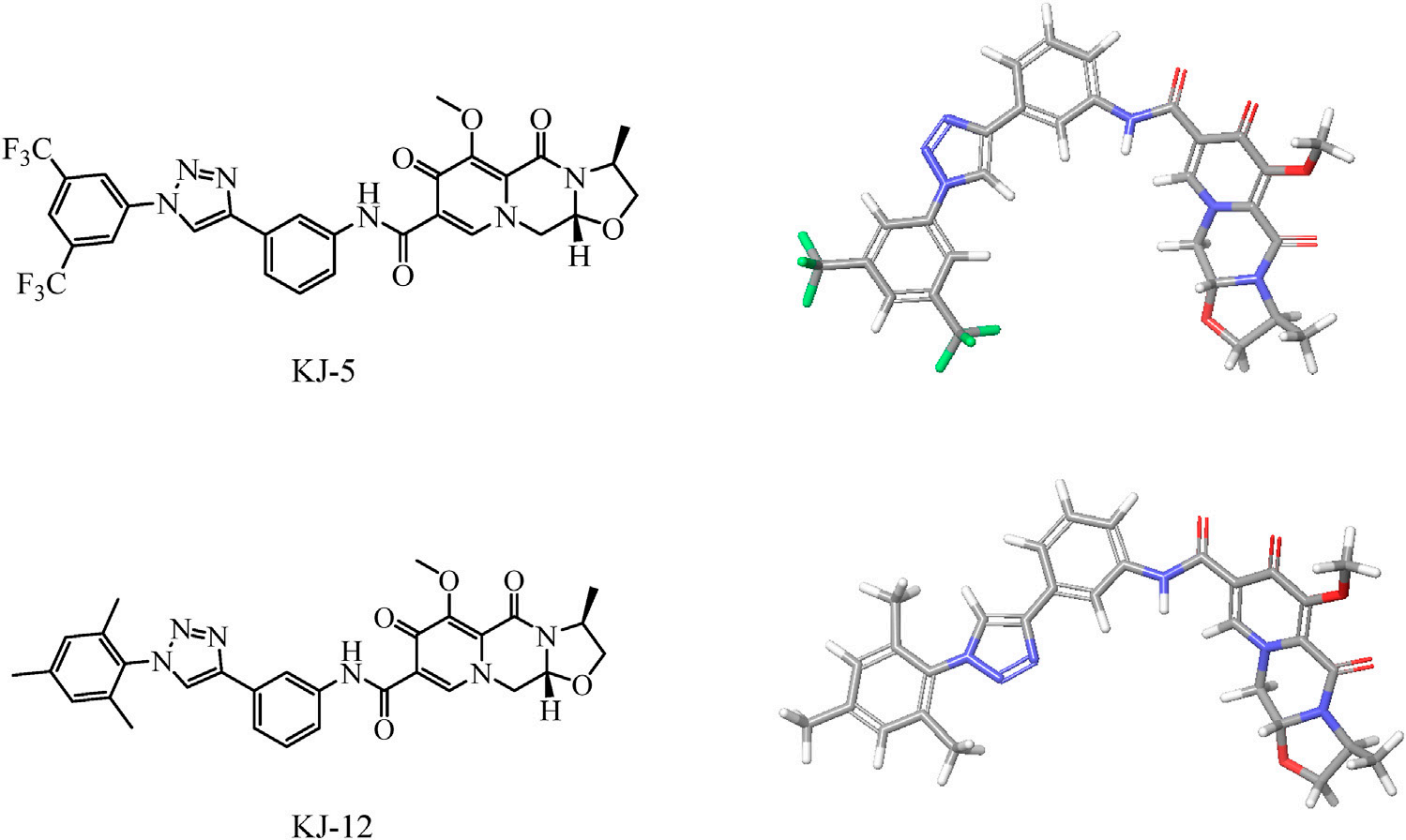
Energy minimization molecular structure of KJ-5 and KJ-12.

Based on the structure of the anti-HIV drug cabotegravir (Johns et al., 2013; Andrews and Heneine, 2015), we introduced 1,2,3-triazole groups with different substituents to obtain 19 cabotegravir derivatives. We tested their activity against HepG2 cells and found that most of the compounds showed good inhibitory activity. Among them, compounds **KJ-5** and **KJ-12** were the most prominent and could effectively inhibit the growth of HepG2 cells, with IC_50_ values of 4.29 ± 0.10 and 4.07 ± 0.09 μM, respectively. Meanwhile, we analyzed the molecular structures of KJ-5 and KJ-12 by minimizing their molecular structure energy (Figure 5). We found that the overall molecular structure tends to be planar and is composed of multiple rigid planar structures, including two benzene ring structures, a 1,2,3-triazole ring structure, and the mother nucleus structure of captopril. These rigid structures can be inserted into the DNA of tumor cells to cause damage. Accordingly, compounds **KJ-5** and **KJ-12** could significantly cause cell apoptosis, G2/M arrest, and DNA damage, and suppressed invasion and migration in HepG2 cells.

## 2 Chemistry

**FIGURE 6:**
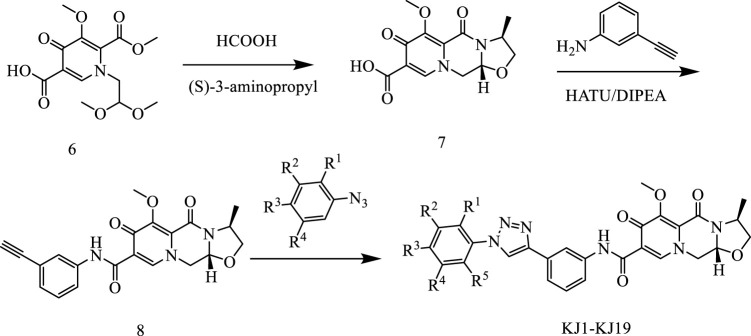
Reaction routes to compounds KJ1–KJ19.

In this way, 1-(2,2-dimethoxyethyl)-5-methoxy-6-(methoxycarbonyl)-4-oxo−1,4-dihydropyridine-3-carboxylic acid (**6**) was used as raw material, and it was hydrolyzed under formic acid. (S)-3-aminopropyl was added directly to the vacuum concentration, and the mixture was refluxed in acetonitrile to give compound **7**. Compound **7** was condensed with 3-amine phenylacetylene to obtain terminal alkyne compound **8**. Compound **8** was reacted with azide compounds of different substituents to obtain 19 novel structure target compounds **KJ1–KJ19** as shown in Figure 6 and Table 1. The structures of the target compound were confirmed by ^1^H and ^13^C nuclear magnetic resonance spectroscopy.

**TABLE 1:** R-group of compounds KJ1–KJ19.

Compound no.	R_1_	R_2_	R_3_	R_4_	R_5_	Compound no.	R_1_	R_2_	R_3_	R_4_	R_5_
KJ-1	CF_3_	H	H	H	H	KJ-11	CF_3_	H	H	CF_3_	H
KJ-2	F	H	H	H	H	KJ-12	CH_3_	H	CH_3_	H	CH_3_
KJ-3	CH_3_	H	H	H	H	KJ-13	Br	H	H	H	H
KJ-4	CH_2_CH_3_	H	H	H	H	KJ-14	H	H	F	H	H
KJ-5	H	CF_3_	H	CF_3_	H	KJ-15	I	H	H	H	H
KJ-6	H	F	H	H	H	KJ-16	H	H	H	H	H
KJ-7	OCH_3_	H	H	H	H	KJ-17	H	Cl	H	H	H
KJ-8	H	CF_3_	H	H	H	KJ-18	H	Br	H	H	H
KJ-9	OCF_3_	H	H	H	H	KJ-19	Cl	H	H	H	H
KJ-10	H	H	CF_3_	H	H						

## 3 Results and discussion

### 3.1 Cabotegravir derivatives suppress cancer cell viability

In order to investigate the anti-proliferative activity of cabotegravir-1,2,3-triazole derivatives to hepatoma carcinoma cells, we performed an MTT assay to detect the effects of all the compounds on the cell viability of the HepG2 cell line (Table 2). We measured and calculated the half-maximal inhibitory concentration (IC_50_) of all the compounds. As shown in Table 2, KJ-5 and KJ-12 were suggested as the most highly active compound against HepG2 cells with IC_50_ values at 4.29 ± 0.10 and 4.07 ± 0.09 μM, respectively. Moreover, the effects of KJ-5 and KJ-12 on the proliferation of other tumor cells were also measured. As shown in Table 3, KJ-5 and KJ-12 also showed good inhibitory effects in three other tumor cell lines (HeLa, MCF-7, and KYSE-30), but the IC_50_ value for HepG2 cells was the lowest. Therefore, HepG2 cells were selected as the research object for further mechanism exploration in subsequent experiments. Additionally, the inhibition rate of KJ-5 and KJ-12 at a concentration of 4 μM on normal hepatic cells L02 is less than 20%, while the inhibition rate on liver cancer cells can reach 50%, suggesting that these two compounds have a certain selective effect on tumor cells (Table 4).

**TABLE 2:** Half-maximal inhibitory concentration (IC_50_) of all the compounds.

Compound no.	IC_50(_μΜ), 48 h	Compound no.	IC_50(_μΜ), 48 h
	HepG2		HepG2
KJ-1	13.64 ± 1.09	KJ-11	>50
KJ-2	25.27 ± 2.07	**KJ-12**	**4.07 ± 0.09**
KJ-3	19.47 ± 0.50	KJ-13	>50
KJ-4	15.14 ± 0.28	KJ-14	9.94 ± 0.78
**KJ-5**	**4.29 ± 0.10**	KJ-15	41.83 ± 1.22
KJ-6	7.63 ± 1.20	KJ-16	28.82 ± 0.10
KJ-7	21.66 ± 1.07	KJ-17	9.83 ± 0.85
KJ-8	9.41 ± 1.30	KJ-18	6.57 ± 1.45
KJ-9	5.06 ± 0.06	KJ-19	7.50 ± 1.19
KJ-10	>50	Cabotegravir	>50

The bold values represent the IC_50_ value of KJ-5 and KJ-12 with significant inhibitory effects.

**TABLE 3:** Half-maximal inhibitory concentration (IC50) of KJ-5 and KJ-12 in other tumor cell lines.

Compound no.	IC_50_ (μM), 48 h		
	HeLa	MCF-7	KYSE-30
KJ-5	5.02 ± 0.94	8.41 ± 0.14	8.51 ± 0.43
KJ-12	5.17 ± 0.34	8.55 ± 0.60	11.73 ± 0.21

**TABLE 4:** Inhibitory rate of KJ-5 and KJ-12 in L02 cells.

Compound no.	Inhibition rate (%), 48 h				
	1 μM	2 μM	4 μM	8 μM	16 μM
KJ-5	9.85 ± 1.60	14.88 ± 1.97	18.04 ± 0.13	27.05 ± 1.03	34.88 ± 2.22
KJ-12	10.77 ± 0.95	13.58 ± 0.75	16.67 ± 1.14	22.26 ± 2.93	30.46 ± 1.20

### 3.2 KJ-5 and KJ-12 induce apoptosis in HepG2 cells

**FIGURE 7:**
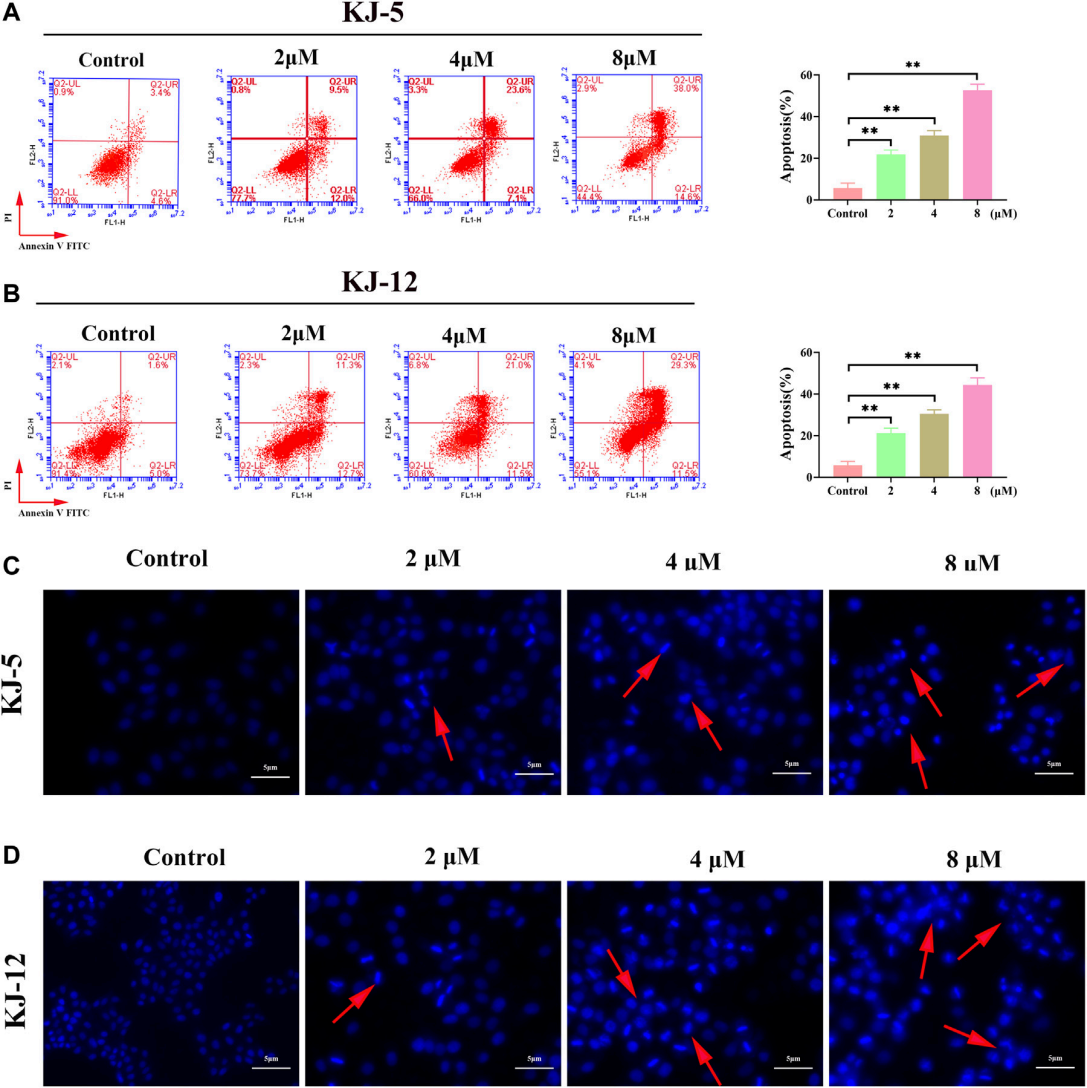
Cell apoptosis induced by KJ-5 and KJ-12 in HepG2 cells. **(A,B)** Apoptosis quantification detected by Annexin V-FITC/PI staining; **(C,D)**. Cell apoptosis morphological changes detected by DAPI staining. Data were mean ± SD. *n* = 3 for each concentration. *p < 0.05, **p < 0.01, vs. control group.

To investigate whether the anti-proliferative effects of these compounds were associated with apoptosis, we further tested compounds KJ-5 or KJ-12, which exhibited potent inhibition of the growth of HepG2 cells. HepG2 cells were treated with various concentrations of KJ-5 or KJ-12 for 24 h, stained with Annexin-V and PI, and measured the percentage of apoptotic cells by flow cytometry. As shown in Figures 7A, B, both KJ-5 and KJ-12 significantly caused cell apoptosis in a concentration-dependent manner. The proportions of apoptotic cells treated with KJ-5 were (21.8 ± 1.3) % (2 μM), (30.9 ± 1.4) % (4 μM), and (52.7 ± 1.7) % (8 μM). The proportions of apoptotic cells treated with KJ-12 were (21.2 ± 1.4) % (2 μM), (30.5 ± 1.1) % (4 μM), and (44.4 ± 1.9) % (8 μM). These results confirmed that both KJ-5 and KJ-12 promoted apoptosis of HepG2 cells.

To further testify apoptosis induced by compounds KJ-5 and KJ-12, HepG2 cells were treated with various concentrations of KJ-5 and KJ-12 and then stained with DAPI. As shown in Figures 7C, D, cell shrinkage, nuclear condensation, and chromatin fragmentation, indicative of apoptosis, were observed in KJ-5 and KJ-12 cells.

### 3.3 KJ-5 and KJ-12 cause G2/M arrest in HepG2 cells

**FIGURE 8:**
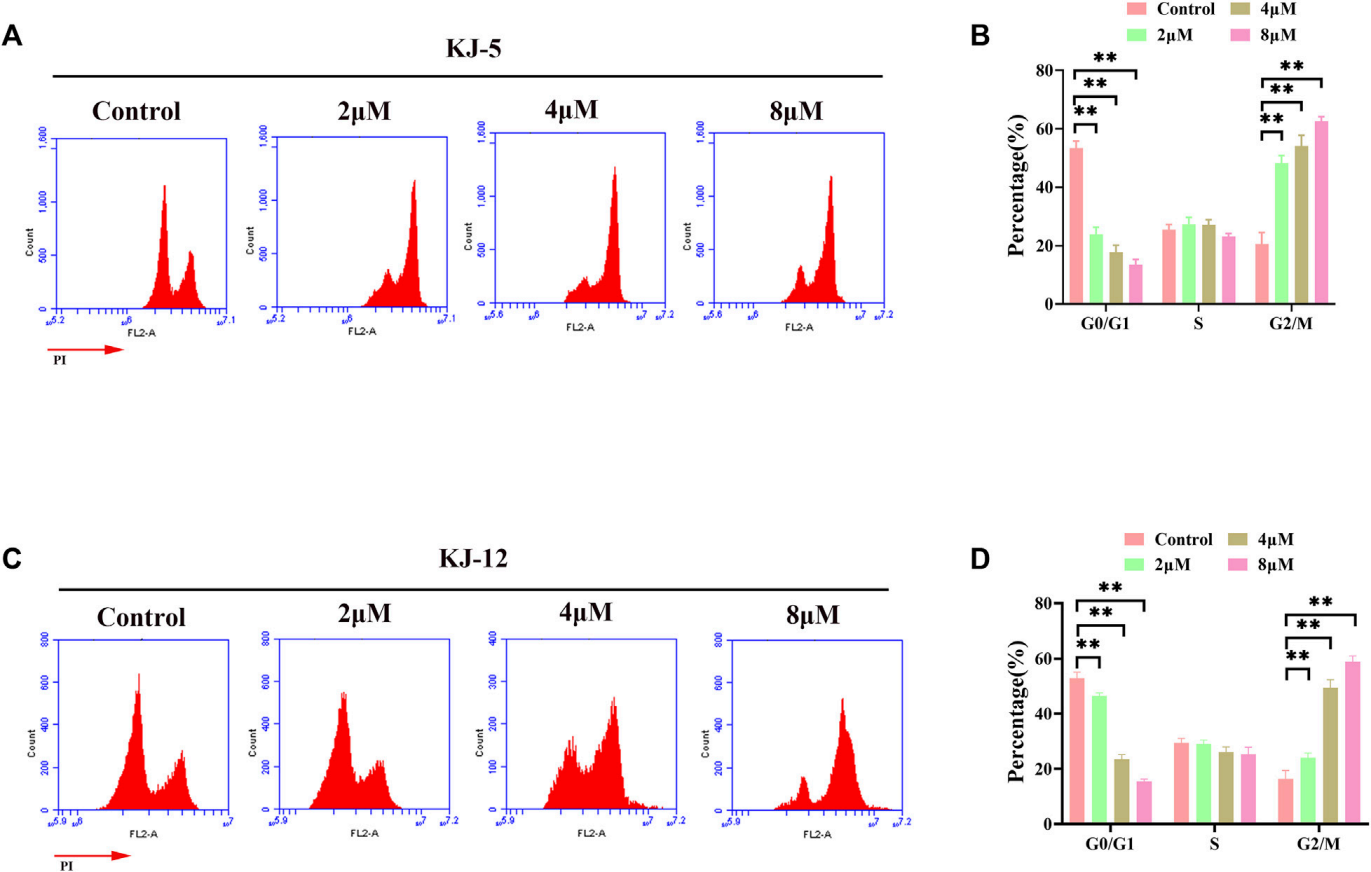
Cell cycle arrests induced by KJ-5 and KJ-12 in HepG2 cells. **(A,B)** Cell cycle arrests induced by KJ-5 in HepG2 cells. **(C,D)** Cell cycle arrests induced by KJ-12 in HepG2 cells. Data were mean ± SD. *n* = 3 for each concentration. *p < 0.05, **p < 0.01, vs. control group.

Flow cytometry analysis was further performed on HepG2 cells after 24 h of treatment with compounds KJ-5 and KJ-12 to examine their effects on the cell cycle phases. As shown in Figures 8A, B, the percentage of KJ-5-treated cells in the G2/M phase increased in a concentration-dependent manner, from 24.8% in the control group to 50.4% (2 μM), 58.3% (4 μM), and 62.6% (8 μM), while the percentage of cells in the G1 phase decreased correspondingly, from 50.8% in the control group to 21.2% (2 μM), 15% (4 μM), and 13.5% (8 μM). The percentage of cells in the S phase did not change significantly. Additionally, there was also an increase in the fraction of cells in the G2/M phase with an increasing KJ-12 concentration, from 18.3% in the control group to 22.2% (2 μM), 47.5% (4 μM), and 59.7% (8 μM) and a decrease in the fraction of cells in the G1 phase, from 50.4% in the control group to 47.4% (2 μM), 25% (4 μM), and 16.4% (8 μM) (Figures 8C, D). The fraction of cells in the S phase remained unchanged substantially. These results suggested that KJ-5 and KJ-12 caused antiproliferative effects by arresting the cell cycle at the G2/M phase.

### 3.4 KJ-5 and KJ-12 cause DNA damage in HepG2 cells

**FIGURE 9:**
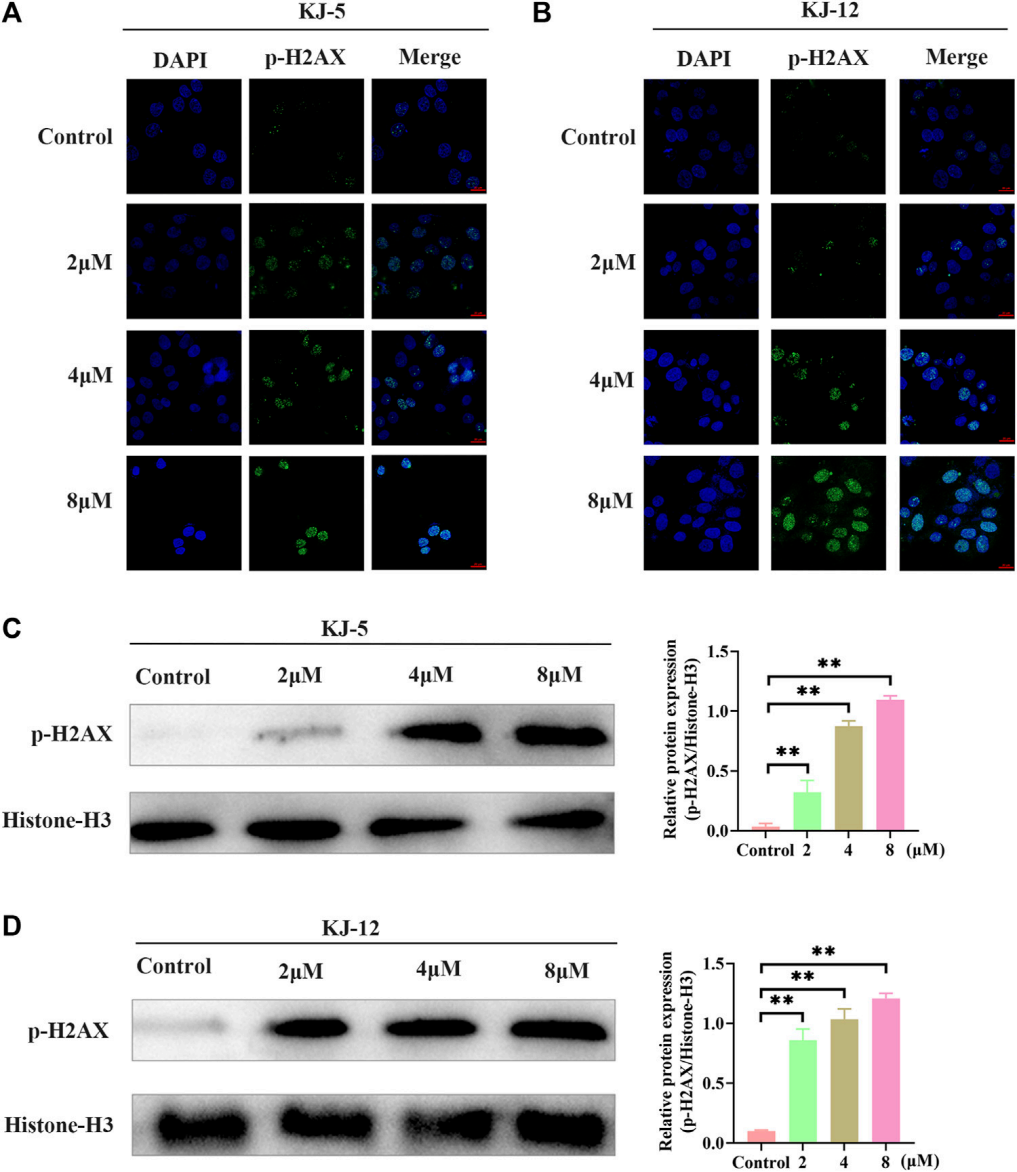
H2Ax phosphorylation induced by KJ-5 and KJ-12 in HepG2 cells. **(A,B)** H2Ax phosphorylation induced by KJ-5 and KJ-12 in HepG2 cells by immunofluorescence. **(C,D)** H2Ax phosphorylation induced by KJ-5 and KJ-12 in HepG2 cells by Western blotting. Data were mean ± SD. *n* = 3 for each concentration. *p < 0.05, **p < 0.01, vs. control group.

The phosphorylation of H2AX is a highly specific and sensitive molecular marker for DNA damage. Immunofluorescence and Western blotting were conducted to detect whether the inhibitory effect of KJ-5 and KJ-12 on HepG2 cells was related to p-H2AX. The results indicated that both KJ-5 and KJ-12 increased the expression of p-H2AX in HepG2 cells, suggesting that the inhibition effect and apoptosis induction of the compounds were related to DNA damage (Figure 9).

### 3.5 KJ-5 and KJ-12 suppress invasion and metastasis in HepG2 cells

**FIGURE 10:**
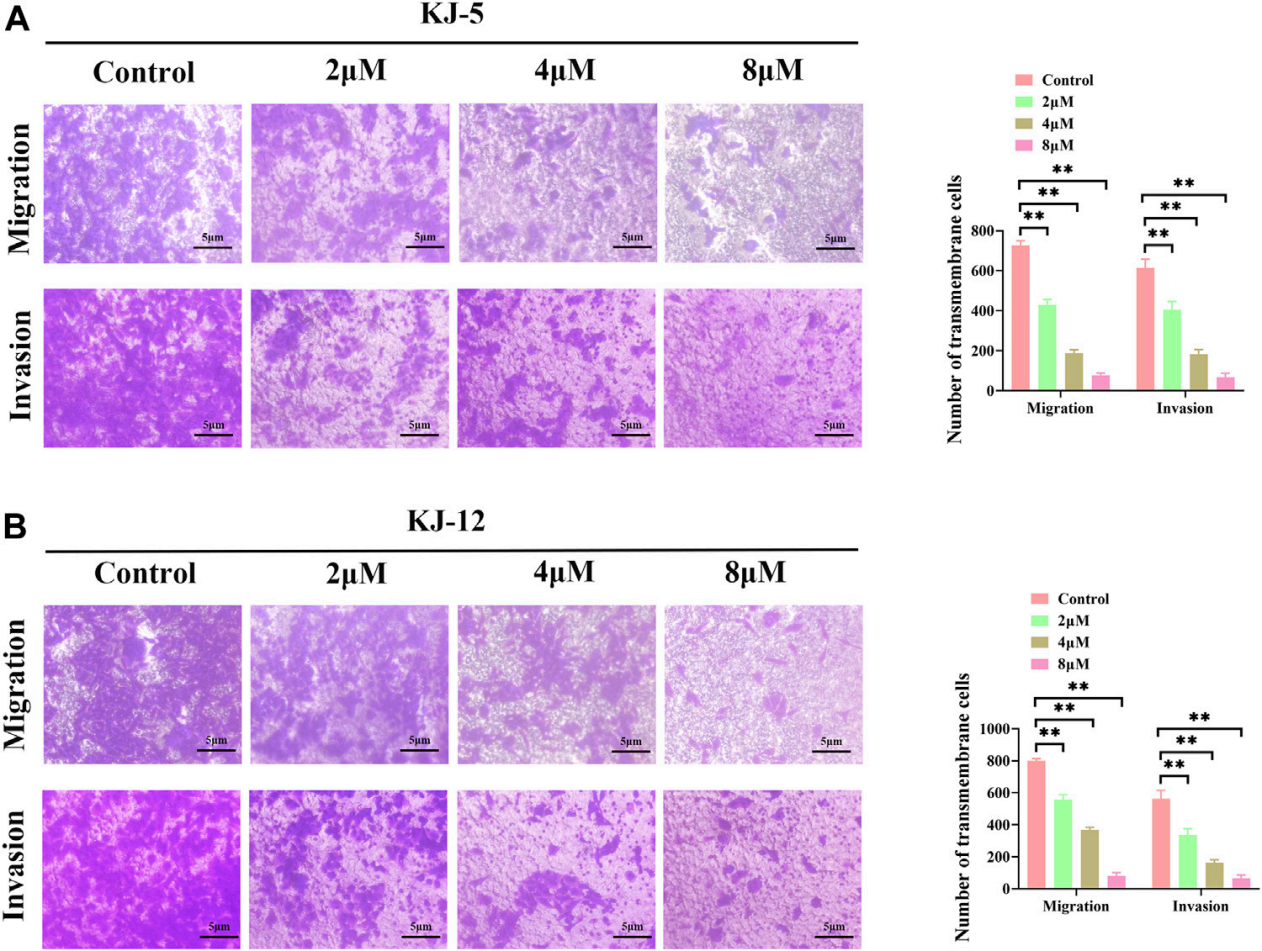
Cell migration and invasion inhibition induced by KJ-5 and KJ-12 in HepG2 cells. **(A,B)** Cell migration and invasion inhibition induced by KJ-5 in HepG2 cells. **(C,D)** Cell migration and invasion inhibition induced by KJ-12 in HepG2 cells. Data were mean ± SD. *n* = 3 for each concentration. *p < 0.05, **p < 0.01, vs. control group.

Metastasis is the leading cause of mortality in hepatocellular carcinoma patients and occurs at a high frequency in this malignancy. To investigate the effects of KJ-5 and KJ-12 on the inhibition of HepG2 cells, transwell migration and invasion with or without Matrigel assays were conducted in HepG2 cells. As shown in Figure 10, either KJ-5 or KJ-12 treatment significantly impaired the migration and invasion of HepG2 cells, as shown by the reduced number of cells that penetrated the lower chamber in transwell assays with or without Matrigel compared with the control group. Taking together, these results demonstrated that KJ-5 and KJ-12 significantly inhibited the migration, invasion, and adhesion of HepG2 cells.

### 3.6 KJ-5 and KJ-12 trigger apoptosis via the mitochondrial pathway

**FIGURE 11:**
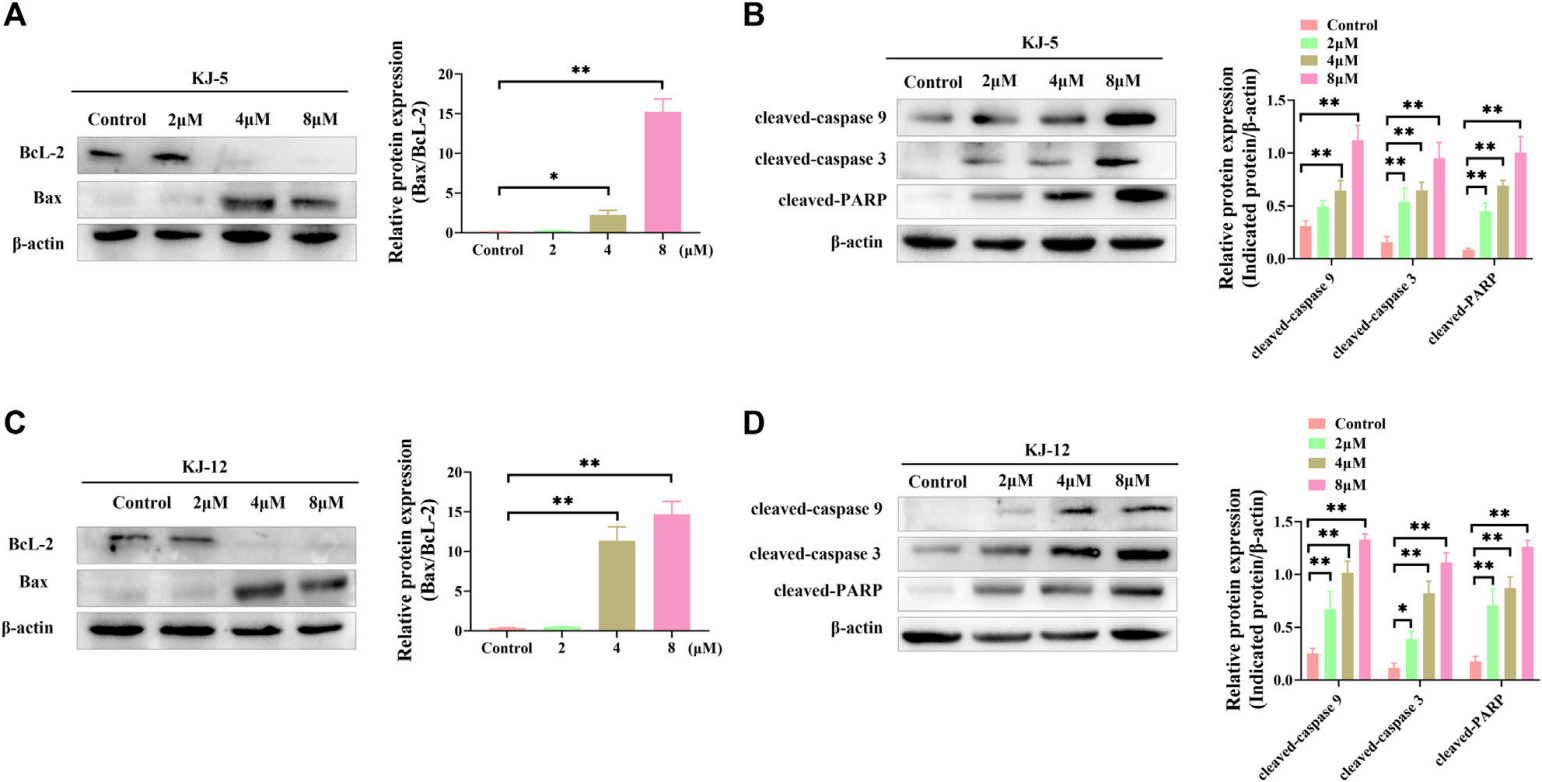
Apoptosis-associated proteins induced by KJ-5 and KJ-12 in HepG2 cells. **(A,B)** Apoptosis-associated protein changes induced by KJ-5 in HepG2 cells. **(C,D)** Apoptosis-associated protein changes induced by KJ-12 in HepG2 cells. Data were mean ± SD. *n* = 3 for each concentration. *p < 0.05, **p < 0.01, vs. control group.

Western blotting was conducted to determine the involvement and expression of some mitochondrial apoptotic proteins, including Bcl-2, Bax, cleaved caspase-9, cleaved caspase-3, and cleaved PARP in KJ-5 and KJ-12-induced HepG2 cell apoptosis. As shown in Figure 11, Bcl-2 protein levels were decreased, Bax levels were increased, and the Bax/Bcl-2 ratio was increased when HepG2 cells were treated with KJ-5 or KJ-12 at the concentrations of 2, 4, or 8 μM. Additionally, the expressions of cleaved caspase-9, cleaved caspase-3, and cleaved PARP were significantly increased in KJ-5-treated HepG2 cells compared with the control group. Meanwhile, the significant increase in cleaved caspase-9, cleaved caspase-3, and cleaved PARP was also observed in KJ-12-treated HepG2 cells compared with the control group. All these results indicated that the activation of the mitochondrial pathway was involved in apoptosis induced by both KJ-5 and KJ-12.

## 4 Conclusion

In summary, we have shown that the cabotegravir derivative demonstrated obvious inhibitory effects of HepG2 cells. In particular, compounds KJ-5 and KJ-12 are particularly prominent in their anti-tumor effects, with IC_50_ values of 4.29 ± 0.10 and 4.07 ± 0.09 μM, respectively. Furthermore, these two compounds could induce cell apoptosis, arrest the G2/M phase, and inhibit the invasion and migration of HepG2 cells. Further study demonstrated that compounds KJ-5 and KJ-12 could trigger apoptosis via the mitochondrial pathway by increasing the ratio of Bax/Bcl-2 and activating cleaved caspase-9, cleaved caspase-3, and cleaved PARP. The promising anti-tumor bioactivity including a significant inhibitory effect, obvious induction of apoptosis, and good suppression of invasion and migration against tumor cells make them ideal candidates for further structural optimization.

## 5 Experiment

### 5.1 Materials and chemistry

Dulbecco’s modified Eagle's medium (DMEM), diamidino-phenyl-indole (DAPI), propidium iodide, RNase A solution, and enhanced chemiluminescence (ECL) solution were purchased from Solarbio Science Technology (Beijing, China). The Annexin V-FITC/PI kit was purchased from Jiangsu KeyGEN Biotech (Nanjing, China). Matrigel was obtained from Corning (NY, United States). Methylthiazolyl tetrazolium biomide (MTT) was purchased from Beijing Labgic Technology (Beijing, China). The primary antibodies against cleaved caspase-3 (1:500), cleaved caspase-9 (1:1000), cleaved PARP (1:750), Bcl-2 (1:500), and Bax (1:1000) were obtained from Wan lei Biotechnology (Shenyang, China). β-actin (1:2000) was purchased from Proteintech (Wuhan, China). HRP-conjugated affinipure goat anti-rabbit IgG (1:1000) was purchased from Cell Signaling Technology (MA, United States).

#### 5.1.1 Synthesis of (3S,11aR)-6-methoxy-3-methyl-5,7-dioxo-2,3,5,7,11,11a-hexahydrooxazolo[3,2-a]pyrido[1,2-d]pyrazine-8-carboxylic acid (compound 7)


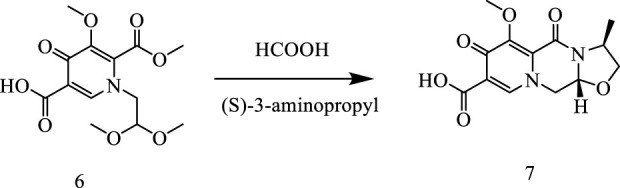


In a reaction flask, 1-(2,2-dimethoxyethyl)-1,4-dihydro-3- methoxy-4-oxo-2,5- pyridinedicarboxylic acid-2-methyl ester (compound **6**, 30 g) was added to 150 mL of anhydrous formic acid. The reaction was carried out at 65°C with stirring and under argon atmosphere. The reaction was completed at about 3 h when the starting material was used up, as monitored with TLC. Under vacuum, formic acid was concentrated and evaporated at 45°C to give crude oil. Then, 150 mL of acetonitrile was added to the crude oil to dissolve with stirring. Furthermore, 10 g of (S)-3-aminopropyl was added and stirred for 10 min. Then, the temperature was raised to an internal temperature of 82°C, and stirring was continued for 5 h. The reaction was concentrated to remove most of the solvent at 45°C. Afterward, 200 mL of dichloromethane was added, and then, 100 mL water was added while stirring. Then, 2N HCl was used to adjust the pH to 1–2, stirred for 10 min, and then the lower organic phase was separated. The organic phase was concentrated under vacuum to give 17.2 g of pure product (Compound **7**).

#### 5.1.2 General synthetic procedure for compound 8


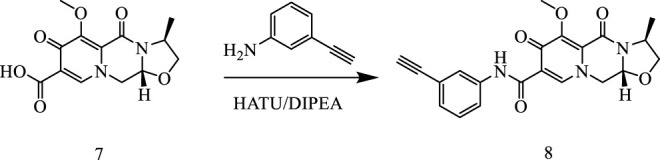


Compound 7 (3 g, 0.01 mol), 3-aminophenylacetylene (2.35 g, 0.02 mol), HATU (7.6 g, 0.02 mol), DIPEA (6.2 g, 0.04 mol), and solvent DMF 200 mL were added to a reaction flask at room temperature and stirred under nitrogen protection for 24 h. After the reaction was completed, DMF was removed with an oil pump, dichloromethane was added, washed with saturated salt water, organic phase was combined, dried with anhydrous sodium sulfate, and concentrated in vacuum to obtain solid compound 8 (4.1 g).

#### 5.1.3 General synthetic procedure for compounds KJ1–KJ19


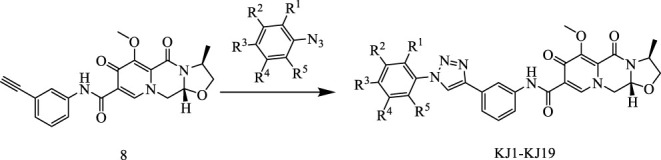


In the reaction flask, compound **8** (1 mmol) and substituted azide (1.2 mmol) were added to 15 mL of a mixed solvent (tetrahydrofuran/water/tert-butanol = 1:1:1). Anhydrous copper sulfate (0.1 mmol) and sodium ascorbate (0.2 mmol) were added, and the mixture was stirred at 80°C for 6 h. After the reaction was completed (monitored by TLC), dichloromethane (10 mL × 5) was used to extract, the organic solution was combined, and the saturated sodium chloride aqueous solution was used to wash (10 mL × 2). The combined organic layer was washed with brine (10 mL × 2), dried over sodium sulfate, and concentrated *in vacuo* to give the crude product. Recrystallization in ethyl acetate produced the desired compound, which was pure enough for further characterization and anti-tumor study.

The spectroscopic characterization of compounds **KJ1**–**KJ19** is provided as Supplementary Material data.

### 5.2 Biological evaluation

#### 5.2.1 Cell culture

HepG2, HeLa, MCF-7, and KYSE-30 cells were grown in a humidified incubator at 37°C with 5% CO_2_ in DMEM supplemented with 10% fetal bovine serum (FBS), 100 mg/mL streptomycin, and 100 U/mL penicillin.

#### 5.2.2 MTT assay for cell proliferation and cytotoxicity

When HepG2 cells reached 80% confluence, they were treated with 1 mL of trypsin and resuspended at a density of 10^5^ cells/mL. Then, 100 μL of cell suspension was seeded into each well of a 96-well plate and incubated in a humidified atmosphere. The different concentrations of KJ-5 and KJ-12 (0, 1, 2, 4, 8, 16, and 32 μM) were added to each well, and the cells were further incubated for 48 h. Afterward, 20 μL of the MTT solution (5 mg/mL) was added to each well, and incubation was continued for another 4 h. The supernatant was discarded, and 200 μL of the DMSO solution was added to each well. The plate was shaken for 10 min in the dark. The absorbance values were measured at 490 nm using an enzyme-linked detector. The cell inhibition rate (%) was calculated using the following formula: (1-Mean A_490__sample_/Mean A_490 control_) × 100%.

#### 5.2.3 Annexin V-FITC/PI staining for cell apoptosis

HepG2 cells were plated in a 6-well plate at a density of 5 × 10^6^ cells/well and incubated for 24 h. Then, the original culture medium was replaced with 2 mL DMEM containing KJ-5 or KJ-12 (2, 4, and 8 μM) and incubated for 24 h. After incubation with KJ-5 or KJ-12, the cells were washed with cold PBS and then resuspended in 100 µL binding buffer at a concentration of 1 × 10^6^ cells/mL. Then, 5 μl of FITC Annexin V and 5 µl of PI were added to the solution. The cells were gently vortexed and incubated for 15 min at room temperature (25°C) in the dark. Subsequently, 400 µL binding buffer was added to each tube, and the samples were analyzed by flow cytometry (BD Accuri™ C6 Plus) within 1 h.

#### 5.2.4 DAPI staining for cell apoptosis

Apoptotic morphological changes were determined by DAPI staining. HepG2 cells were seeded in a 48-well plate and treated with different concentrations of KJ-5 and KJ-12 (2, 4, and 8 μM) for 24 h. After 24 h, they were then washed with PBS and fixed with 4% paraformaldehyde for 20 min. Subsequently, they were then washed with PBS and treated with DAPI (10 μg/mL) at 37°C for 5 min. After washing with PBS, the cells were resuspended in PBS for the rest of the experiment. A fluorescence microscope was used to observe the apoptotic morphological changes.

#### 5.2.5 Cell cycle analysis

HepG2 cells were seeded in a 6-well plate at a density of 5 × 10^6^ cells/well and treated with either KJ-5 or KJ-12 at concentrations of 2, 4, or 8 μM for 24 h. Cells were then collected and responded in 300 L of cold PBS. Subsequently, 700 μL of cold 70% ethanol was added to the cells and gently mixed by inversion on ice for 1 h. Cells were then centrifuged at 2,000 rpm for 10 min, washed once with 2 mL of cold PBS, and the supernatant was discarded. The cell pellet was resuspended in 250 μL of cold PBS, followed by the addition of 10 μL of RNase at a final concentration of 0.4 mg/mL and incubated at 37°C for 30 min. An additional 250 μL of PBS was added, followed by the addition of 5 μL of PI solution at a final concentration of 10 μg/mL. The samples were incubated at 37°C in the dark overnight at 4°C. Finally, the samples were analyzed by flow cytometry.

#### 5.2.6 Immunofluorescence analysis

HepG2 cells were plated in laser confocal dishes (Nest, China), followed by the addition of KJ-5 or KJ-12 for 24 h. Then, 4% paraformaldehyde was added to each laser confocal dish for 20 min after rinsing with PBS. Before the goat serum was added to block for 1 h, 0.5% Triton X-100 was used to penetrate HepG2 for 30 min. Subsequently, the cells were incubated with p-H2AX (Cell Signaling Technology, MA, United States) at a 1:800 dilution at 4°C overnight. The cells were rinsed with PBS and incubated with Alexa Fluor 488 (Proteintech, Wuhan, China) at a 1:1000 dilution in dark at room temperature for 1 h. DAPI was used to stain the cell nucleus for 5 min. Then, the cells were rinsed with PBS before observing the cells under a laser confocal microscope (Nikon, Tokyo, Japan).

#### 5.2.7 Transwell migration and invasion (Matrigel) assays

HepG2 cells were treated with either KJ-5 or KJ-12 for 24 h. Then, the cells were suspended and seeded into the upper chamber of a 24-well Transwell plate (Corning Inc., United States) in serum-free medium. The cells were cultured for 24 h at 37°C; a medium containing 20% FBS as a chemoattractant was added to the lower chamber. HepG2 cells that had invaded through the membrane were fixed with 4% paraformaldehyde for 20 min and stained with 0.1% crystal violet. Five random fields were then imaged and quantified using an inverted fluorescence microscope (Axio Observer3, Germany). To assess invasion, transwell assays were performed in a similar manner, with the exception of coating the upper chamber of the Transwell with 100 μL of diluted Matrigel solution per well.

#### 5.2.8 Western blot analysis

After being pretreated with either KJ-5 or KJ-12 at concentrations of 2, 4, or 8 μM for 24 h, the cells were harvested and lysed using an ice-cold cell lysis buffer from Beyotime Biotechnology (Shanghai, China). The proteins were mixed with a loading buffer and denatured by heating at 95°C–100°C for 5–10 min. Equal amounts of protein were loaded onto a 10% SDS-PAGE gel along with a molecular weight marker and separated by electrophoresis for 2 h. The separated proteins were transferred onto a PVDF membrane, which was blocked with 5% non-fat milk for non-specific binding sites on the membrane. Then, the membrane was incubated with primary antibodies at 4°C overnight. Following incubation with the primary antibody, the membrane was incubated with HRP-conjugated goat anti-rabbit or goat anti-mouse IgG at 37°C for 1 h. Protein bands were visualized on the PVDF membrane using an ECL hypersensitive chemiluminescence solution and a Bio-Rad gel imaging and analysis system.

#### 5.2.9 Statistical analyses

Data were obtained from one of three independent experiments. Differences between groups were analyzed using one-way analysis of variance (ANOVA), followed by Dunnett’s *t*-test. A *p*-value of less than 0.05 was considered statistically significant, while a *p*-value of less than 0.01 indicated highly significant differences.

## Data Availability

The original contributions presented in the study are included in the article/Supplementary Material; further inquiries can be directed to the corresponding authors.
