# Supplementary material for: Design and synthesis of cabotegravir derivatives bearing 1,2,3-triazole and evaluation of anti-liver cancer activity
Source: Front Pharmacol. 2023 Oct 6;14:1265289. doi: 10.3389/fphar.2023.1265289 (PMC10590056; doi:10.3389/fphar.2023.1265289)
Supplement: Supplementary file 1 [file DataSheet1.zip › supplementary experimental data/1.MTT assay/4.Tables.docx]

**Table.3 The half-maximal inhibitory concentration (IC50) of KJ-5 and KJ-12**

**in other tumor cell lines**

| Compd no. | IC_50_ (μM)，48 h | | |
| --- | --- | --- | --- |
|  | Hela | MCF-7 | KYSE-30 |
|  |  |  |  |
| KJ-5 | 5.02±0.94 | 8.41±0.14 | 8.51±0.43 |
| KJ-12 | 5.17±0.34 | 8.55±0.60 | 11.73±0.21 |

**Table.4 The inhibitory rate of KJ-5 and KJ-12 in L02 cells**

| Compd no. | Inhibition rate (%)，48 h | | | | |
| --- | --- | --- | --- | --- | --- |
|  | 1μM | 2μM | 4μM | 8μM | 16μM |
| KJ-5 | 9.85±1.60 | 14.88±1.97 | 18.04±0.13 | 27.05±1.03 | 34.88±2.22 |
| KJ-12 | 10.77±0.95 | 13.58±0.75 | 16.67±1.14 | 22.26±2.93 | 30.46±1.20 |

**Table. Preliminary screening of 19 compounds (mean±SD)**

| Compd no. | Inhibition rate (16μM)，48 h | | | |
| --- | --- | --- | --- | --- |
|  | HepG2 | Hela | MCF-7 | KYSE-30 |
| KJ-1 | 46.83±3.00 | 44.98±3.26 | 44.37±5.97 | 39.31±3.16 |
| KJ-2 | 51.03±0.23 | 47.99±4.29 | 47.43±2.55 | 44.92±6.50 |
| KJ-3 | 43.68±3.13 | 37.61±4.86 | 38.91±5.12 | 32.35±5.86 |
| KJ-4 | 50.88±0.63 | 45.95±6.42 | 47.13±5.83 | 43.8±6.15 |
| KJ-5 | 85.21±0.73 | 79.39±1.04 | 62.65±1.76 | 65.68±0.91 |
| KJ-6 | 60.48±0.82 | 56.13±4.18 | 52.3±1.16 | 54.65±6.31 |
| KJ-7 | 41.07±0.48 | 35.68±4.35 | 41.2±4.56 | 44.77±3.26 |
| KJ-8 | 63.99±1.68 | 65.81±3.35 | 58.85±3.95 | 59.67±1.33 |
| KJ-9 | 80.72±1.18 | 75.9±1.83 | 68.32±5.95 | 63.87±4.34 |
| KJ-10 | 32.59±1.79 | 33.99±1.59 | 33.36±3.87 | 26.17±7.59 |
| KJ-11 | 24.66±1.73 | 22.31±2.4 | 18.01±3.00 | 23.45±2.69 |
| KJ-12 | 76.92±2.40 | 71.86±1.86 | 54.67±1.79 | 56.13±4.59 |
| KJ-13 | 24.19±0.79 | 19.16±1.71 | 25.6±3.81 | 25.16±3.96 |
| KJ-14 | 55.74±1.15 | 51.02±5.15 | 51.15±2.92 | 52.98±4.08 |
| KJ-15 | 35.56±0.75 | 28.08±4.59 | 35.25±1.83 | 40.93±1.83 |
| KJ-16 | 40.70±2.68 | 35.41±1.09 | 41.19±2.3 | 39.76±2.74 |
| KJ-17 | 62.85±0.70 | 59.34±4.79 | 55.76±5.48 | 55.22±4.84 |
| KJ-18 | 70.90±2.87 | 64.65±1.09 | 58.01±2.89 | 57.48±4.60 |
| KJ-19 | 73.27±1.09 | 63.13±0.49 | 57.71±4.31 | 55.43±2.87 |
