# Supplementary material for: Design and synthesis of cabotegravir derivatives bearing 1,2,3-triazole and evaluation of anti-liver cancer activity
Source: Front Pharmacol. 2023 Oct 6;14:1265289. doi: 10.3389/fphar.2023.1265289 (PMC10590056; doi:10.3389/fphar.2023.1265289)

**Design and Synthesis of cabotegravir derivatives bearing 1,2,3-triazole and evaluation of anti-liver cancer activity**

Huaxia Xie ^†a^, Longfei Mao^†a^, Gaolu Fan^b^, Ziyuan Wu ^a^, Yimian Wang ^a^, Xixi Hou^c^, Jian gang Wang ^a^, Huili Wang^d^, Ling Liu^a^*, Sanqiang Li^a^*

^a^ College of Basic Medicine and Forensic Medicine, Henan University of Science and Technology, Luoyang 471023, China

^b^ Department of Pharmacy, Luoyang third people’ hospital, Luoyang 471000, China

^c^Department of Pharmacy, The First Affiliated Hospital, and College of Clinical Medicine of Henan University of Science and Technology, Luoyang, 471003, China

^d^University of North Carolina Hospitals, 101 Manning Dr, Chapel Hill, Orange County, NC 27599, USA

†These authors have contributed equally to this work as first co-authors

*Corresponding Author

**Ling Liu**, College of Basic Medicine and Forensic Medicine, Henan University of Science and Technology. 263 Kaiyuan Road, Luoyang 471003, China. E-mail: liuling921@126.com

**San-qiang Li,** College of Basic Medicine and Forensic Medicine, Henan University of Science and Technology, 263 Kaiyuan Road, Luoyang 471003, China. E-mail: sanqiangli2001@163.com

**Compound** KJ1；– MS (ESI): *m/z* (%) = 581 [M + H]^+^. Pure 98.1%. white solid, HR-MS(ESI): Calcd. C28H23F3N6O5 [M+H]^+^ *m/z*: 581.1760, found: 581.1789. m.p. 177-180 ^o^C. ^1^H NMR(400MHz, DMSO-d_6_): 12.60 (d, J=8.0Hz, 1H), 9.10 (s, 1H), 8.72 (s, 1H), 8.25 (s, 1H), 8.08 (d, J=8.0Hz, 1H), 7.98 (t, J_1_=8.0Hz, J_2_=8.0Hz, 1H), 7.92-7.82 (m, 3H), 7.69 (d, J=8.0Hz, 1H), 7.50 (t, J_1_=8.0Hz, J_2_=8.0Hz, 1H), 5.54-5.34 (m, 1H), 4.98-4.79 (m, 1H), 4.46-4.36 (m, 1H), 4.19-4.05 (m, 2H), 3.92 (d, J=4.0Hz, 3H), 3.25-3.04 (m, 1H), 1.37-1.30 (m, 3H); ^13^C NMR(100MHz, DMSO-d_6_): 174.20, 162.27, 154.50, 154.34, 152.06, 147.75, 144.21, 139.51, 137.59, 131.86, 131.32, 130.36, 125.68, 125.46, 124.35, 121.34, 120.68, 120.18, 118.15, 117.08, 116.92, 82.74, 82.27, 74.75, 74.16, 61.00, 55.49, 54.96, 49.95, 18.41

**Compound** KJ2：– MS (ESI): *m/z* (%) = 531 [M + H]^+^. Pure 99.2%. white solid, HR-MS(ESI): Calcd. C27H23FN6O5 [M+H]^+^ *m/z*: 531.1792, found: 531.1825. m.p. 199-202 ^o^C. ^1^H NMR(400MHz, DMSO-d_6_): 12.59 (s, 1H), 9.16 (s, 1H), 8.71 (s, 1H), 8.23 (s, 1H), 7.92 (t, J_1_=8.0Hz, J_2_=4.0Hz, 1H), 7.85 (d, J=4.0Hz, 1H), 7.71 (d, J=4.0Hz, 1H), 7.66-7.60 (m, 2H), 7.50 (dd, J_1_=4.0Hz, J_2_=8.0Hz, 2H), 5.54-5.34 (m, 1H), 4.96-4.79 (m, 1H), 4.46-4.36 (m, 2H), 3.92 (d, J=4.0Hz, 3H), 3.25-3.04 (m, 1H), 1.37-1.30 (m, 3H)；^13^C NMR(100MHz, DMSO-d_6_): 174.11, 162.27, 154.34, 153.09, 152.02, 147.11, 144.24, 144.13, 139.47, 131.85, 131.63, 131.32, 126.50, 126.11, 123.63, 121.46, 120.10, 118.14, 117.79, 116.97, 82.74, 74.15, 60.99, 55.45, 49.95, 18.14.

**Compound** KJ3：– MS (ESI): *m/z* (%) = 527 [M + H]^+^. Pure 96.4%. gray solid, HR-MS(ESI): Calcd. C28H27N6O5 [M+H]^+^ *m/z*: 527.2043, found: 527.2053. m.p. 188-191 ^o^C. ^1^H NMR(400MHz, DMSO-d_6_): 12.59 (s, 1H), 9.02 (s, 1H), 8.71 (s, 1H), 8.21 (s, 1H), 7.84 (d, J=4.0Hz, 1H), 7.69 (d, J=4.0Hz, 1H), 7.54-7.44 (m, 4H), 5.53-5.34 (m, 1H), 4.97-4.79 (m, 1H), 4.45-4.36 (m, 1H), 4.18-4.05 (m, 2H), 3.91 (d, J=4.0Hz, 3H), 3.25-3.04 (m, 1H), 1.37-1.30 (m, 3H). ^13^C NMR(100MHz, DMSO-d_6_):174.17, 162.25, 154.51, 152.02, 146.65, 144.12, 139.43, 136.75, 133.48, 131.91, 130.26, 127.52, 126.46, 123.81, 121.42, 119.92, 118.15, 116.89, 82.74, 82.27, 74.74, 74.15, 60.99, 55.37, 54.81, 49.55, 18.39, 17.99.

**Compound** KJ4：– MS (ESI): *m/z* (%) = 541 [M + H]^+^. Pure 97.8%. white solid, HR-MS(ESI): Calcd. C29H28N6O5 [M+H]^+^ *m/z*: 541.2199, found: 541.2210. m.p. 170-173 ^o^C. ^1^H NMR(400MHz, DMSO-d_6_): 12.60 (s, 1H), 9.02 (s, 1H), 8.72 (s, 1H), 8.22 (s, 1H), 7.84 (d, J=8.0Hz, 1H), 7.69 (d, J=4.0Hz, 1H), 7.59-7.45 (m, 5H), 5.54-5.34 (m, 1H), 4.98-4.80 (m, 1H), 4.46-4.36 (m, 1H), 4.19-4.04 (m, 2H), 3.91 (d, J=4.0Hz, 3H), 3.25-3.04 (m, 1H), 2.56-2.52 (m, 2H), 1.37-1.30 (m, 3H), 1.07 (t, J_1_=8.0Hz, J_2_=8.0Hz, 3H). ^13^C NMR(100MHz, DMSO-d_6_):174.10, 162.23, 154.32, 151.99, 146.65, 144.21, 139.75, 136.21, 131.69, 130.73, 130.37, 130.25, 127.47, 126.89, 124.11, 121.40, 119.92, 118.03, 116.88, 82.73, 82.27, 74.73, 74.14, 60.97, 55.43, 49.94, 24.30, 18.38, 15.33

**Compound** KJ5：– MS (ESI): *m/z* (%) = 649 [M + H]^+^. Pure 98.3%. white solid, HR-MS(ESI): Calcd. C29H22F6N6O5 [M+H]^+^ *m/z*: 649.1634, found: 649.1650. m.p. 274-277 ^o^C. ^1^H NMR(400MHz, DMSO-d_6_): 12.63 (d, J=4.0Hz, 1H), 9.67 (s, 1H), 8.69 (s, 1H), 8.29 (s, 1H), 8.15 (s, 1H), 7.86 (d, J=8.0Hz, 1H), 7.68 (d, J=8.0Hz, 1H), 7.51 (t, J_1_=8.0Hz, J_2_=8.0Hz, 1H), 5.55-5.34 (m, 1H), 4.98-4.79 (m, 1H), 4.47-4.36 (m, 1H), 4.19-4.05 (m, 2H), 3.92-3.91 (m, 3H), 3.25-3.04 (m, 1H), 1.38-1.31 (m, 3H). ^13^C NMR(100MHz, DMSO-d_6_): 174.15, 162.23, 154.31, 151.96, 147.89, 144.18, 139.51, 138.31, 132.50, 132.16, 130.99, 130.41, 124.64, 121.92, 120.93, 120.23, 118.10, 116.77, 82.72, 82.26, 74.74, 74.15, 60.98, 55.43, 54.80, 49.94, 18.39

**Compound** KJ6：– MS (ESI): *m/z* (%) = 531 [M + H]^+^. Pure 96.6%. white solid, HR-MS(ESI): Calcd. C27H23FN6O5 [M+H]^+^ *m/z*: 531.1792, found: 531.1810. m.p. 211-214 ^o^C. ^1^H NMR(400MHz, DMSO-d_6_): 12.68 (s, 1H), 9.50 (s, 1H), 8.77 (s, 1H), 8.22 (s, 1H), 7.98-7.93 (m, 3H), 7.78-7.74 (m, 2H), 7.57 (t, J_1_=4.0Hz, J_2_=4.0Hz, 1H), 7.45 (t, J_1_=4.0Hz, J_2_=8.0Hz, 1H), 5.60-5.40 (m, 1H), 5.03-4.85 (m, 1H), 4.52-4.42 (m, 1H), 4.24-4.11 (m, 2H), 3.98-3.97 (m, 3H), 3.31-3.11 (m, 1H), 1.43-1.36 (m, 3H). ^13^C NMR(100MHz, DMSO-d_6_): 174.13, 164.16, 162.26, 154.50, 152.02, 147.61, 144.11, 139.50, 138.27, 132.45, 131.32, 130.37, 121.31, 120.56, 120.13, 118.13, 116.86, 116.34, 115.82, 108.07, 82.74, 82.27,74.74, 74.15, 60.99, 55.38, 18.39

**Compound** KJ7：– MS (ESI): *m/z* (%) = 543 [M + H]^+^. Pure 95.7%. white solid, HR-MS(ESI): Calcd. C28H26N6O6 [M+H]^+^ *m/z*: 543.1992, found: 543.2007. m.p. 253-256 ^o^C. ^1^H NMR(400MHz, DMSO-d_6_): 12.59 (s, 1H), 9.00 (s, 1H), 8.74 (s, 1H), 8.23 (s, 1H), 7.84 (d, J=8.0Hz, 1H), 7.72-7.70 (m, 2H), 7.59 (t, J_1_=8.0Hz, J_2_=8.0Hz, 1H), 7.50 (t, J_1_=8.0Hz, J_2_=8.0Hz, 1H), 7.39-7.37 (m, 1H), 7.20 (t, J_1_=8.0Hz, J_2_=8.0Hz, 1H), 5.56-5.36 (m, 1H), 5.00-4.80 (m, 1H), 4.48-4.37 (m, 1H), 4.21-4.06 (m, 2H), 3.94-3.92 (m, 6H), 3.28-3.06 (m, 1H), 1.39-1.32 (m, 3H). ^13^C NMR(100MHz, DMSO-d_6_): 174.17, 162.25, 154.51, 152.31, 152.01, 146.39, 144.23, 139.42, 131.76, 131.39, 130.23, 126.43, 126.20, 124.14, 121.36, 119.17, 118.17, 116.86, 113.49, 82.74, 82.28, 74.74, 74.15, 60.99, 56.66, 55.44, 49.95, 18.39

**Compound** KJ8：– MS (ESI): *m/z* (%) = 581 [M + H]^+^. Pure 97.3%. white solid, HR-MS(ESI): Calcd. C28H23F3N6O5 [M+H]^+^ *m/z*: 581.1760, found: 581.1776. m.p. 160-163 ^o^C. ^1^H NMR(400MHz, DMSO-d_6_): 12.64 (s, 1H), 9.58 (s, 1H), 8.73 (s, 1H), 8.39-8.36 (m, 1H), 8.21(s, 1H), 7.93-7.89 (m, 3H), 7.73 (d, J=8.0Hz, 1H), 7.54 (t, J_1_=8.0Hz, J_2_=8.0Hz, 1H), 5.57-5.36 (m, 1H), 5.00-4.81 (m, 1H), 4.49-4.38 (m, 1H), 4.20-4.19 (m, 1H), 4.12-4.09 (m, 1H), 3.94-3.93 (m, 3H), 3.28-3.06 (m, 1H), 1.40-1.32 (m, 3H). ^13^C NMR(100MHz, DMSO-d_6_):174.19, 162.27, 154.33, 152.02, 147.73, 144.11, 139.51, 137.58, 131.88, 131.31, 130.39, 125.74, 125.01, 124.34, 121.32, 120.71, 120.16, 118.00, 116.88, 82.28, 74.16, 67.40, 61.00, 55.46, 49.56, 49.07, 31.78, 18.40

**Compound** KJ9：– MS (ESI): *m/z* (%) = 597 [M + H]^+^. Pure 95.2%. white solid, HR-MS(ESI): Calcd. C28H23F3N6O6 [M+H]^+^ *m/z*: 597.1709, found: 597.1713. m.p. 263-266 ^o^C. ^1^H NMR(400MHz, DMSO-d_6_): 12.62 (s, 1H), 9.17 (s, 1H), 8.74 (d, J=4.0Hz, 1H), 8.26 (s, 1H), 7.96-7.94 (m, 1H), 7.87-7.85 (m, 1H), 7.79-7.78 (m, 2H), 7.74-7.70 (m, 2H), 7.52 (t, J_1_=8.0Hz, J_2_=8.0Hz, 1H), 5.56-5.36 (m, 1H), 5.00-4.80 (m, 1H), 4.48-4.38 (m, 1H), 4.21-4.06 (m, 2H), 3.94-3.93 (m, 3H), 3.28-3.06 (m, 1H), 1.39-1.32 (m, 3H). ^13^C NMR(100MHz, DMSO-d_6_):174.18, 162.27, 154.50, 154.33, 152.01, 146.95, 144.24, 141.61, 139.50, 132.18, 131.64, 131.30, 130.35, 130.18, 129.36, 128.06, 123.96, 121.41, 120.12, 116.93, 82.74, 74.74, 60.99, 55.45, 49.95, 18.41

**Compound** KJ10：– MS (ESI): *m/z* (%) = 581 [M + H]^+^. Pure 98.3%. white solid, HR-MS(ESI): Calcd. C28H23F3N6O5 [M+H]^+^ *m/z*: 581.1760, found: 581.1769. m.p. 216-219 ^o^C. ^1^H NMR(400MHz, DMSO-d_6_): 12.65 (s, 1H), 9.58 (s, 1H), 8.74 (d, J=4.0Hz, 1H), 8.29-8.22 (m, 3H), 8.07 (d, J=8.0Hz, 1H), 7.92-7.90 (m, 1H), 7.75-7.73 (m, 1H), 7.54 (t, J_1_=8.0Hz, J_2_=8.0Hz, 1H), 5.57-5.36 (m, 1H), 5.00-4.81 (m, 1H), 4.49-4.38 (m, 1H), 4.21-4.07 (m, 2H), 3.94-3.93 (m, 3H), 3.28-3.08 (m, 1H), 1.40-1.32 (m, 3H). ^13^C NMR(150MHz, DMSO-d_6_):173.12, 161.20, 153.26, 150.94, 146.74, 143.15, 138.43, 130.59, 130.16, 129.31, 126.71, 120.28, 119.79, 119.55, 119.12, 116.92, 115.83, 81.66, 81.19, 73.66, 73.07, 59.91, 54.38, 48.87, 17.31,

**Compound** KJ11：– MS (ESI): *m/z* (%) = 649 [M + H]^+^. Pure 94.6%. white solid, HR-MS(ESI): Calcd. C29H22F6N6O5 [M+H]^+^ *m/z*: 649.1634, found: 649.1679. m.p. 172-175 ^o^C. ^1^H NMR(400MHz, DMSO-d_6_): 12.63 (s, 1H), 9.20 (s, 1H), 8.73 (d, J=4.0Hz, 1H), 8.46 (s, 1H), 8.37-8.27 (m, 3H), 7.86-7.83 (m, 1H), 7.72-7.70 (m, 1H), 7.53 (t, J_1_=8.0Hz, J_2_=8.0Hz, 1H), 5.56-5.36 (m, 1H), 5.00-4.80 (m, 1H), 4.49-4.38 (m, 1H), 4.21-4.07 (m, 2H), 3.94-3.90 (m, 3H), 3.28-3.08 (m, 1H), 1.39-1.32 (m, 3H). ^13^C NMR(100MHz, DMSO-d_6_): 174.18, 162.28, 154.33, 152.02, 146.86, 144.11, 139.53, 135.62, 131.51, 130.40, 129.76, 128.69, 127.11, 125.17, 124.06, 121.39, 120.18, 118.00, 116.95, 82.74, 82.28, 74.74, 74.15, 60.98, 55.46, 54.82, 49.55, 18.39

**Compound** KJ12：– MS (ESI): *m/z* (%) = 555 [M + H]^+^. Pure 97.1%. white solid, HR-MS(ESI): Calcd. C30H30N6O5 [M+H]^+^ *m/z*: 555.2356, found: 555.2397. m.p. 181-184 ^o^C. ^1^H NMR(400MHz, DMSO-d_6_): 12.59-12.57 (m, 1H), 8.85 (s, 1H), 8.71 (s, 1H), 8.21 (s, 1H), 7.82 (d, J=4.0Hz, 1H), 7.67 (d, J=8.0Hz, 1H), 7.48 (t, J_1_=8.0Hz, J_2_=8.0Hz, 1H), 7.13 (s, 2H), 5.53-5.34 (m, 1H), 4.96-4.79 (m, 1H), 4.45-4.36 (m, 1H), 4.18-4.12 (m, 1H), 4.09-4.05 (m, 1H), 3.91-3.90 (m, 3H), 3.25-3.04 (m, 1H), 2.35 (s, 3H), 1.96 (s, 6H), 1.37-1.30 (m, 3H). ^13^C NMR(100MHz, DMSO-d_6_): 174.11, 162.24, 154.34, 152.00, 146.61, 144.22, 140.07, 139.43, 134.96, 133.87, 131.83, 130.24, 129.41, 124.19, 121.39, 119.87, 118.03, 116.88, 82.74, 74.74, 74.15, 60.98, 55.45, 49.95, 21.14, 18.42, 17.37

**Compound** KJ13：– MS (ESI): *m/z* (%) = 591 [M + H]^+^. Pure 99.1%. white solid, HR-MS(ESI): Calcd. C27H23BrN6O5 [M+H]^+^ *m/z*: 591.0992, found: 591.1010. m.p. 207-210 ^o^C. ^1^H NMR(400MHz, DMSO-d_6_): 12.60-12.59 (m, 1H), 9.10 (s, 1H), 8.71 (s, 1H), 8.23 (s, 1H), 8.00-7.95 (m, 1H), 7.89-7.82 (m, 1H), 7.77-7.48 (m, 5H), 5.54-5.34 (m, 1H), 4.98-4.78 (m, 1H), 4.48-4.34 (m, 1H), 4.19-4.04 (m, 2H), 3.91-3.90 (m, 3H), 3.25-3.03 (m, 1H), 1.37-1.30 (m, 3H). ^13^C NMR(100MHz, DMSO-d_6_): 174.18, 162.26, 154.34, 152.03, 146.64, 144.23, 139.47, 136.68, 134.15, 132.56, 131.50, 130.31, 129.48, 129.18, 124.39, 121.40, 120.44, 120.02, 119.37, 118.03, 116.90, 82.74, 82.27, 74.74, 74.15, 60.98, 56.50, 49.94, 19.01

**Compound** KJ14：– MS (ESI): *m/z* (%) = 531 [M + H]^+^. Pure 98.0%. white solid, HR-MS(ESI): Calcd. C27H23FN6O5 [M+H]^+^ *m/z*: 531.1792, found: 531.1821. m.p. 277-280 ^o^C. ^1^H NMR(400MHz, DMSO-d_6_): 12.61 (d, J=4.0Hz, 1H), 9.36 (s, 1H), 8.71 (s, 1H), 8.17 (s, 1H), 8.06-8.02 (m, 1H), 7.87 (d, J=8.0Hz, 1H), 7.70 (d, J=8.0Hz, 1H), 7.51 (t, J_1_=8.0Hz, J_2_=8.0Hz, 3H), 5.55-5.35 (m, 1H), 4.98-4.79 (m, 1H), 4.47-4.36 (m, 1H), 4.21-4.05 (m, 2H), 3.92 (d, J=4.0Hz, 3H), 3.27-3.05 (m, 1H), 1.38-1.31 (m, 3H). ^13^C NMR(100MHz, DMSO-d_6_): 174.14, 162.26, 160.95, 154.34, 152.06, 147.53, 144.10, 139.48, 133.73, 131.63, 131.51, 130.31, 122.87, 121.34, 120.63, 120.06, 118.17, 117.38, 117.15, 116.90, 82.76, 74.16, 61.00, 55.49, 54.85, 49.56, 18.41

**Compound** KJ15：– MS (ESI): *m/z* (%) = 639 [M + H]^+^. Pure 96.6%. white solid, HR-MS(ESI): Calcd. C27H23IN6O5 [M+H]^+^ *m/z*: 639.0853, found: 639.0868. m.p. 181-184 ^o^C. ^1^H NMR(400MHz, DMSO-d_6_): 12.61 (d, J=4.0Hz, 1H), 9.40 (s, 1H), 8.72 (s, 1H), 8.18 (s, 1H), 8.00-7.98 (m, 1H), 7.88 (d, J=8.0Hz, 1H), 7.72-7.63 (m, 3H), 7.55-7.49 (m, 3H), 5.55-5.34 (m, 1H), 4.98-4.79 (m, 1H), 4.46-4.35 (m, 1H), 4.19-4.05 (m, 2H), 3.92-3.91 (m, 3H), 3.25-3.04 (m, 1H), 1.37-1.30 (m, 3H). ^13^C NMR(100MHz, DMSO-d_6_): 174.18, 162.25, 154.50, 151.99, 147.49, 144.22, 144.11, 139.47, 137.12, 131.54, 130.42, 130.31, 129.21, 121.32, 120.44, 120.36, 120.01, 118.02, 116.85, 99.99, 82.27, 74.15, 60.99, 54.80, 49.55, 18.41

**Compound** KJ16：– MS (ESI): *m/z* (%) = 513 [M + H]^+^. Pure 94.9%. white solid, HR-MS(ESI): Calcd. C27H24N6O5 [M+H]^+^ *m/z*: 513.1886, found: 513.1895. m.p. 229-232 ^o^C. ^1^H NMR(400MHz, DMSO-d_6_): 12.63 (d, J=4.0Hz, 1H), 9.41 (s, 1H), 8.73 (d, J=4.0Hz,1H), 8.18 (s, 1H), 8.01-7.98 (m, 1H), 7.88 (d, J=12.0Hz, 1H), 7.72-7.63 (m, 3H), 7.55-7.49 (m, 3H), 5.55-5.34 (m, 1H), 4.98-4.79 (m, 1H), 4.48-4.36 (m, 1H), 4.20-4.05 (m, 2H), 3.92 (d, J=4.0Hz, 3H), 3.26-3.04 (m, 1H), 1.37-1.30 (m, 3H). ^13^C NMR(100MHz, DMSO-d_6_):174.18, 162.26, 154.34, 152.00, 147.49, 144.13, 139.46, 137.11, 131.66, 131.54, 130.43, 130.33, 129.23, 121.34, 120.44, 118.13, 116.84, 82.73, 82.27, 74.74, 74.15, 60.99, 55.44, 49.55, 18.39

**Compound** KJ17：– MS (ESI): *m/z* (%) = 547 [M + H]^+^. Pure 97.5%. white solid, HR-MS(ESI): Calcd. C27H23ClN6O5 [M+H]^+^ *m/z*: 547.1497, found: 547.1510. m.p. 204-207 ^o^C. ^1^H NMR(600MHz, DMSO-d_6_): 12.62 (d, J=6.0Hz, 1H), 9.47 (s, 1H), 8.71 (d, J=6.0Hz, 1H), 8.16 (s, 1H), 8.12 (t, J_1_=6.0Hz, J_2_=6.0Hz, 1H), 8.02-8.00 (m, 1H), 7.88 (d, J=12.0Hz, 1H), 7.70-7.66 (m, 2H), 7.60 (d, J=12.0Hz, 1H), 7.51 (t, J_1_=6.0Hz, J_2_=6.0Hz, 1H), 5.54-5.34 (m, 1H), 4.97-4.79 (m, 1H), 4.46-4.37 (m, 1H), 4.18-4.05 (m, 2H), 3.91(d, J=6.0Hz, 3H), 3.25-3.05 (m, 1H), 1.37-1.30 (m, 3H). ^13^C NMR(150MHz, DMSO-d_6_): 174.19, 162.26, 154.50, 154.33, 152.00, 147.62, 144.21, 144.11, 139.50, 138.15, 134.73, 132.18, 131.65, 131.53, 131.33, 130.37, 128.99, 121.30, 120.55, 120.22, 119.01, 118.01, 116.85, 82.74, 74.75, 74.15, 60.99, 55.46, 49.95, 18.40

**Compound** KJ18：– MS (ESI): *m/z* (%) = 591 [M + H]^+^. Pure 96.7%. white solid, HR-MS(ESI): Calcd. C27H23BrN6O5 [M+H]^+^ *m/z*: 591.0992, found: 591.0998. m.p. 231-234 ^o^C. ^1^H NMR(600MHz, DMSO-d_6_): 12.62 (d, J=6.0Hz, 1H), 9.46 (s, 1H), 8.71 (d, J=6.0Hz, 1H), 8.24 (s, 1H), 8.16 (s, 1H), 8.05-8.04 (m, 1H), 7.88 (d, J=12.0Hz, 1H), 7.74-7.68 (m, 2H), 7.60 (t, J_1_=12.0Hz, J_2_=6.0Hz, 1H), 7.51 (t, J_1_=6.0Hz, J_2_=12.0Hz, 1H), 5.54-5.34 (m, 1H), 4.97-4.79 (m, 1H), 4.46-4.37 (m, 1H), 4.18-4.05 (m, 2H), 3.92 (d, J=6.0Hz, 3H), 3.25-3.04 (m, 1H), 1.37-1.30 (m, 3H). ^13^C NMR(150MHz, DMSO-d_6_): 174.19, 162.25, 154.50, 154.33, 152.02, 147.60, 144.10, 139.50, 138.22, 132.38, 131.90, 131.51, 131.33, 130.35, 122.95, 121.29, 120.53, 120.11, 119.38, 118.14, 116.84, 82.28, 74.15, 60.99, 54.82, 49.56, 18.40

**Compound** KJ19：– MS (ESI): *m/z* (%) = 547 [M + H]^+^. Pure 95.4%. white solid, HR-MS(ESI): Calcd. C27H23ClN6O5 [M+H]^+^ *m/z*: 547.1497, found: 547.1511. m.p. 259-262 ^o^C. ^1^H NMR(600MHz, DMSO-d_6_): 12.60 (d, J=6.0Hz, 1H), 9.12 (s, 1H), 8.72 (d, J=6.0Hz, 1H), 8.23 (s, 1H), 7.84-7.79 (m, 3H), 7.69-7.62 (m, 3H), 7.50 (t, J_1_=6.0Hz, J_2_=12.0Hz, 1H), 5.54-5.34 (m, 1H), 4.97-4.79 (m, 1H), 4.45-4.36 (m, 1H), 4.18-4.05 (m, 2H), 3.91 (d, J=6.0Hz, 3H), 3.25-3.04 (m, 1H), 1.37-1.30 (m, 3H). ^13^C NMR(150MHz, DMSO-d_6_): 174.18, 162.27, 154.34, 152.01, 146.70, 144.23, 139.48, 134.99, 132.27, 131.63, 131.44, 131.10, 130.32, 129.01, 128.92, 124.39, 121.42, 120.05, 118.15, 116.92, 82.74, 74.74, 74.15, 60.99, 55.45, 49.95, 18.42

# Figure 1. ^1^H NMR and ^13^C NMR spectrums of compound KJ1


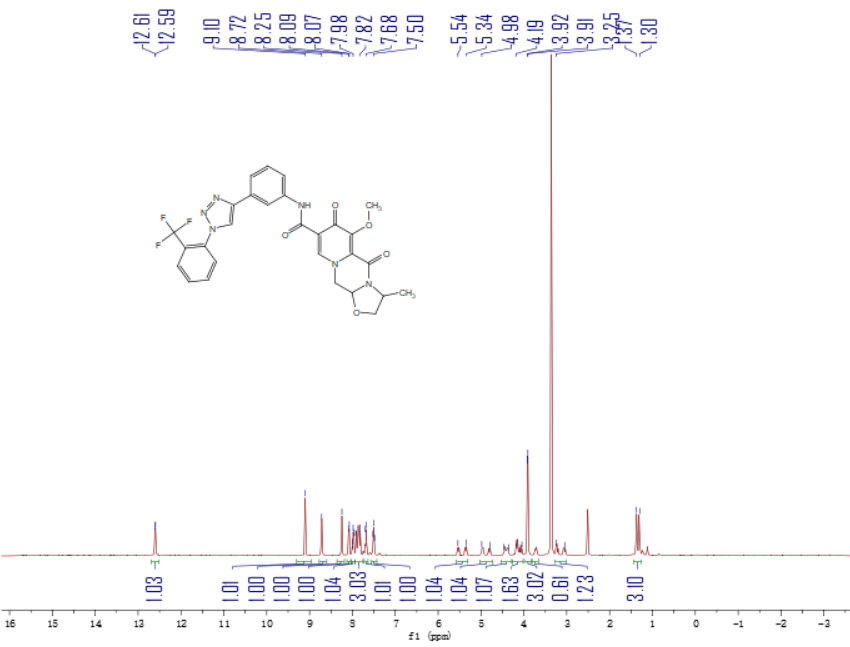


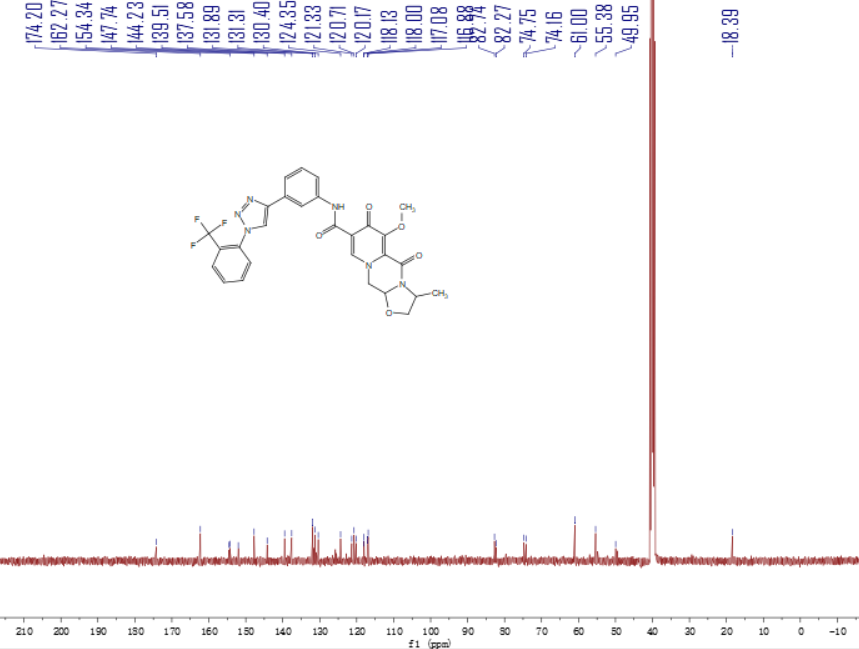


# Figure 2. ^1^H NMR and ^13^C NMR spectrums of compound KJ2


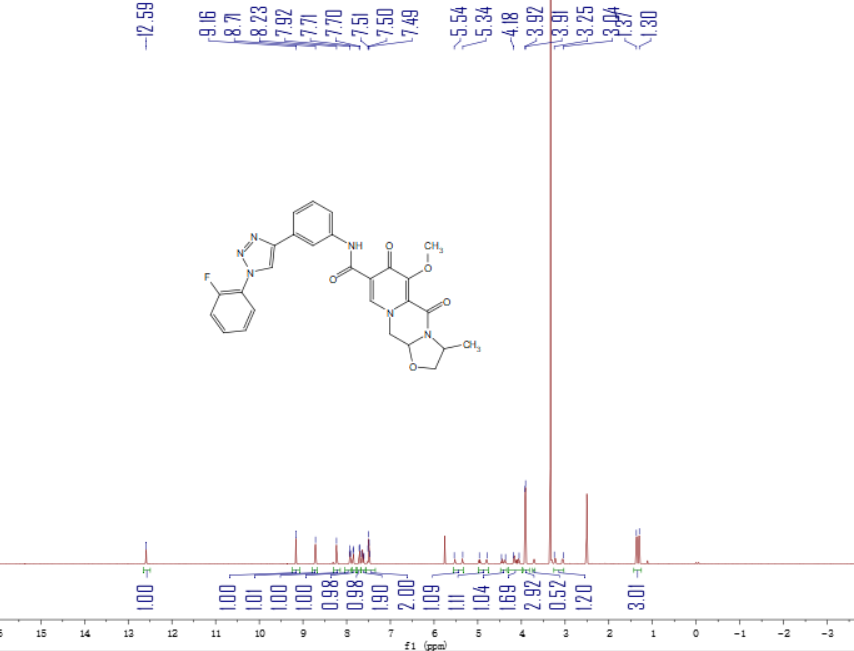


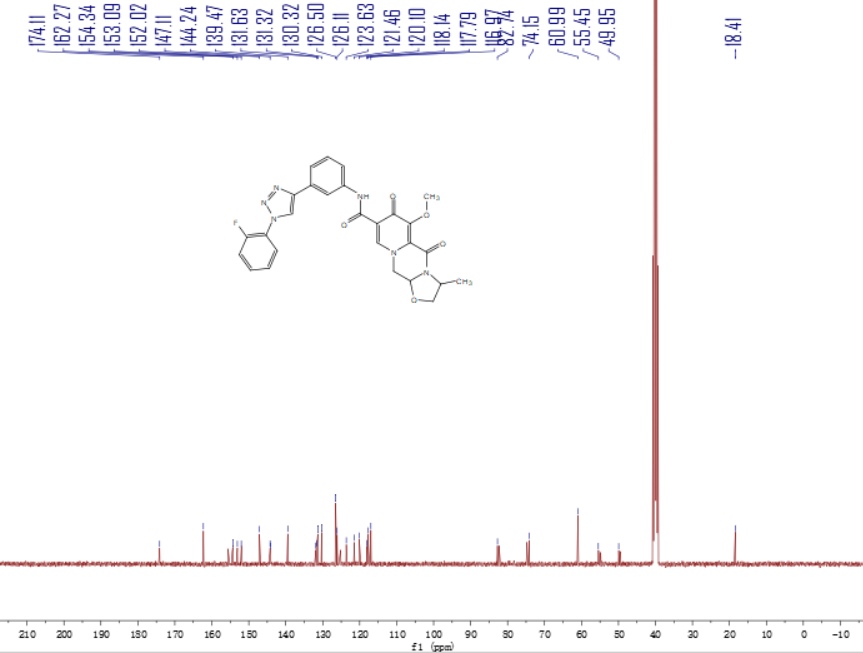


# Figure 3. ^1^H NMR and ^13^C NMR spectrums of compound KJ3


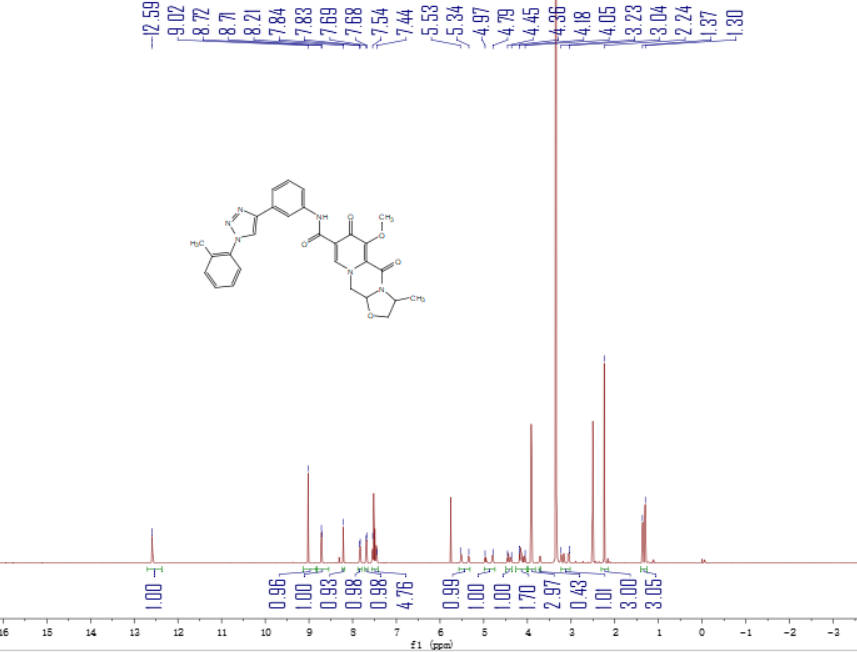


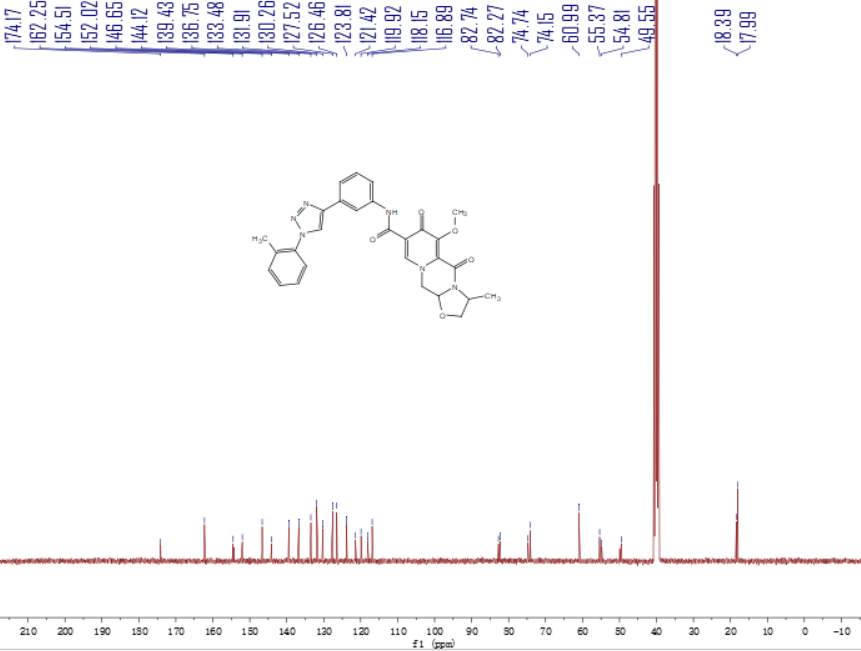


# Figure 4. ^1^H NMR and ^13^C NMR spectrums of compound KJ4


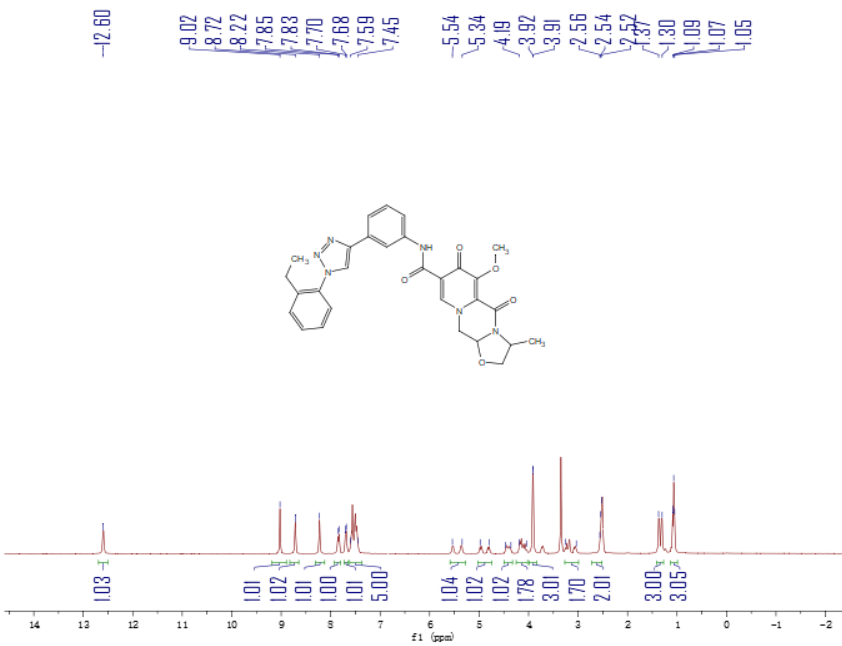


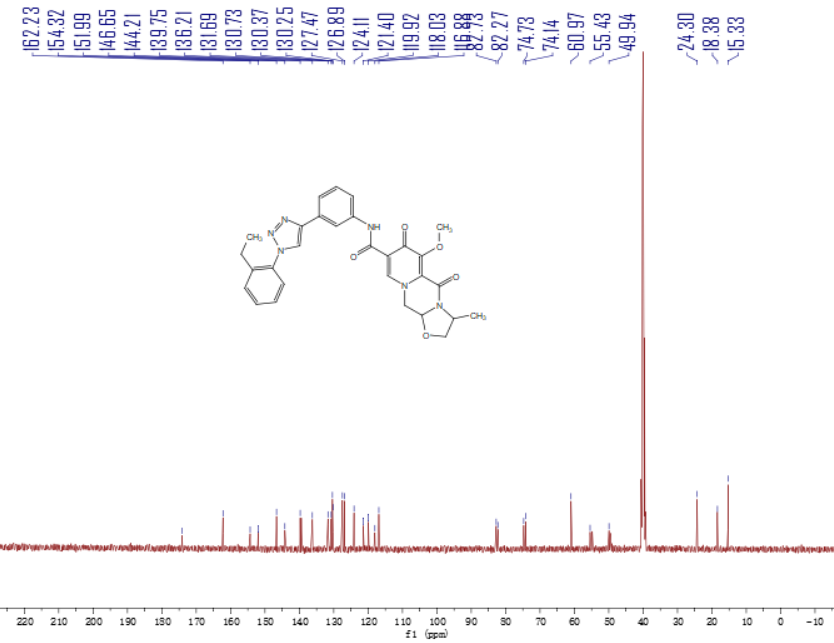


# Figure 5. ^1^H NMR and ^13^C NMR spectrums of compound KJ5


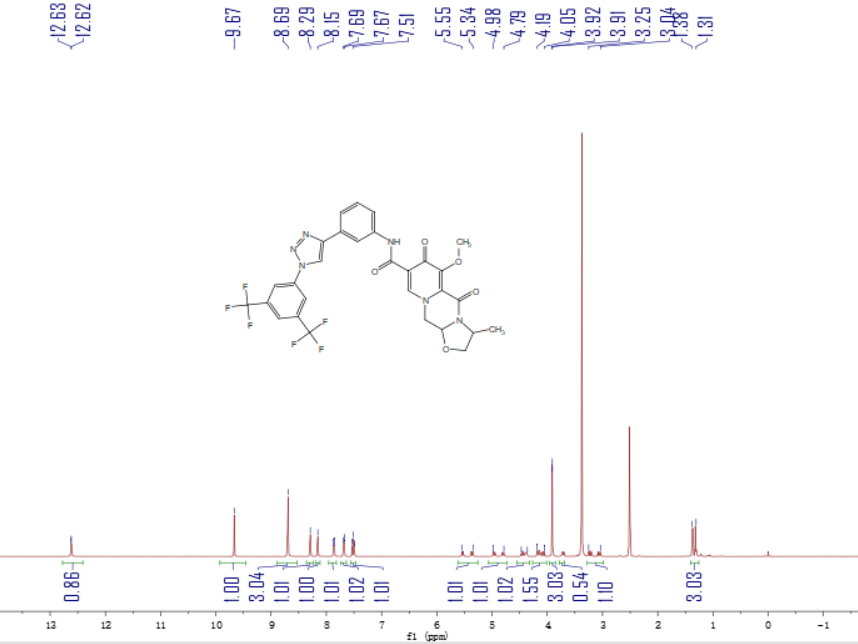


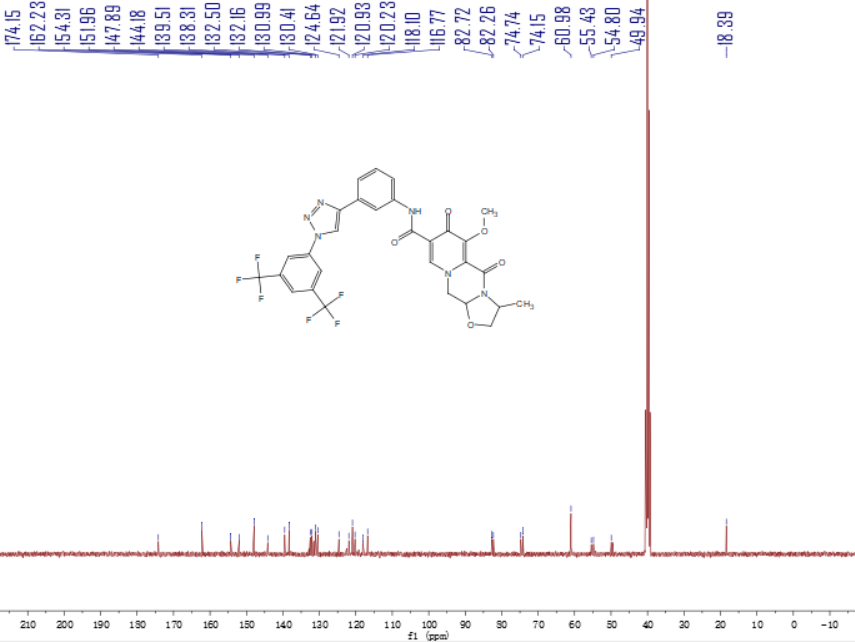


# Figure 6. ^1^H NMR and ^13^C NMR spectrums of compound KJ6


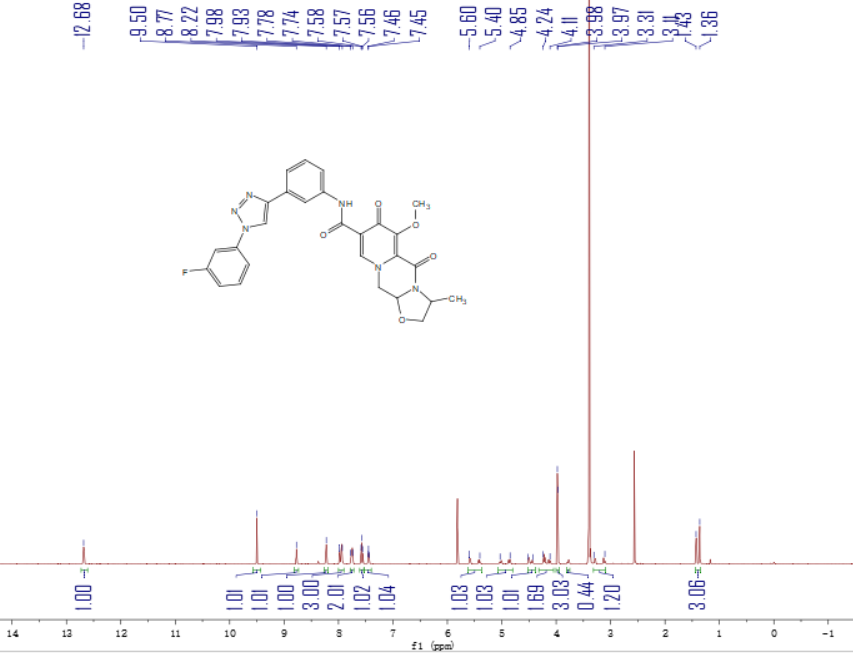


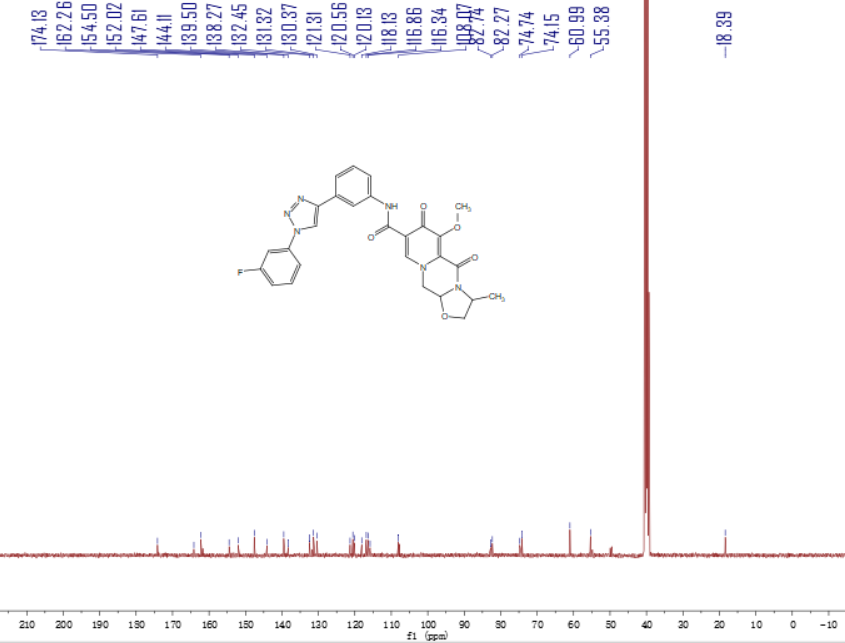


# Figure 7. ^1^H NMR and ^13^C NMR spectrums of compound KJ7


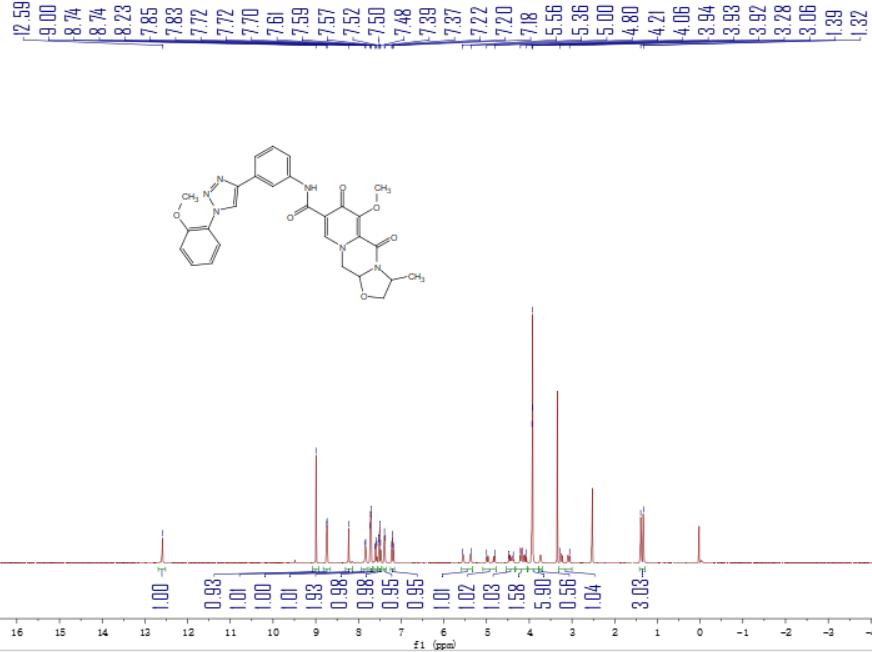


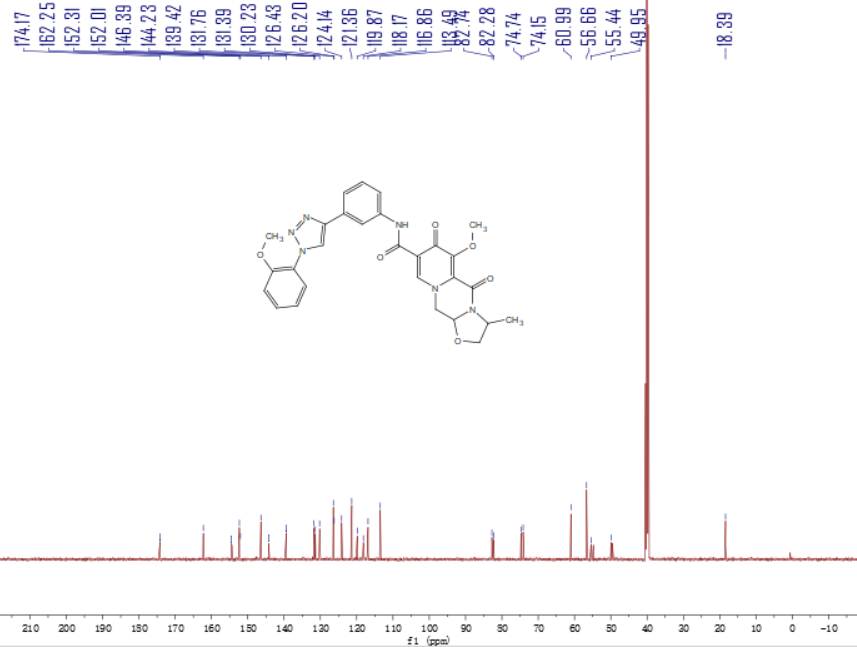


# Figure 8. ^1^H NMR and ^13^C NMR spectrums of compound KJ8


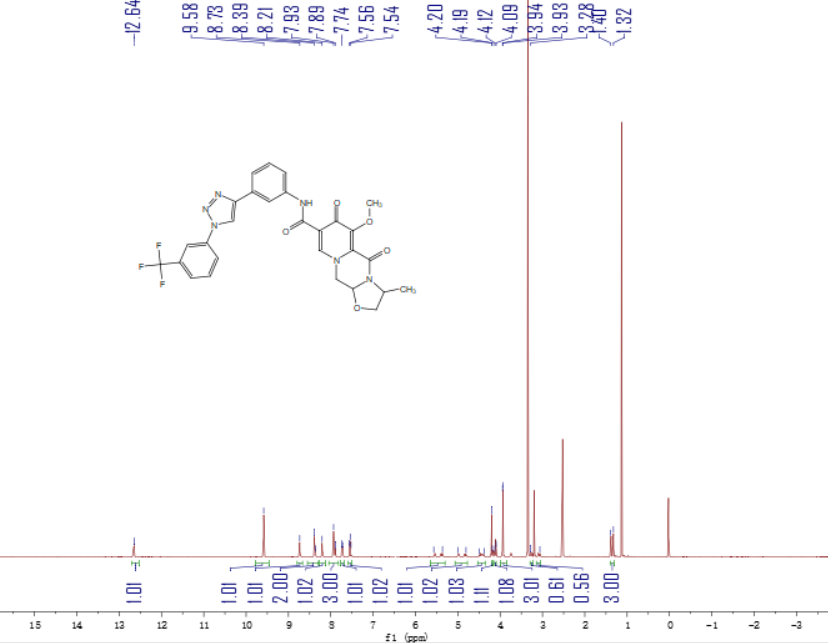


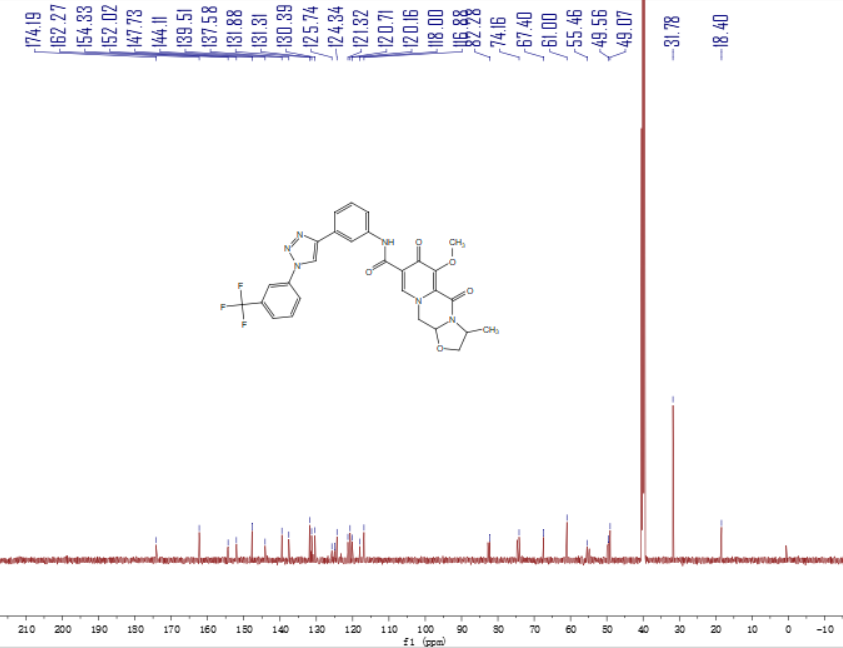


# Figure 9. ^1^H NMR and ^13^C NMR spectrums of compound KJ9


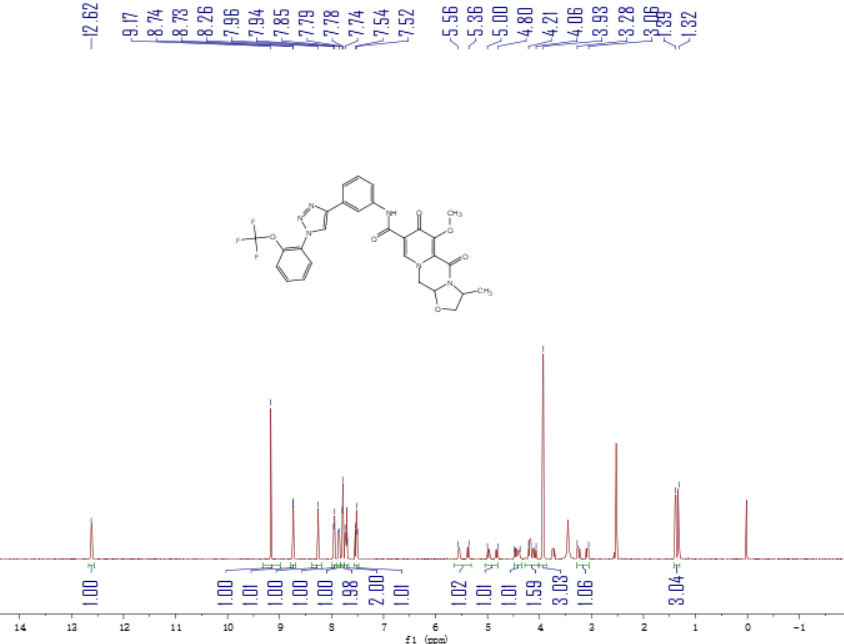


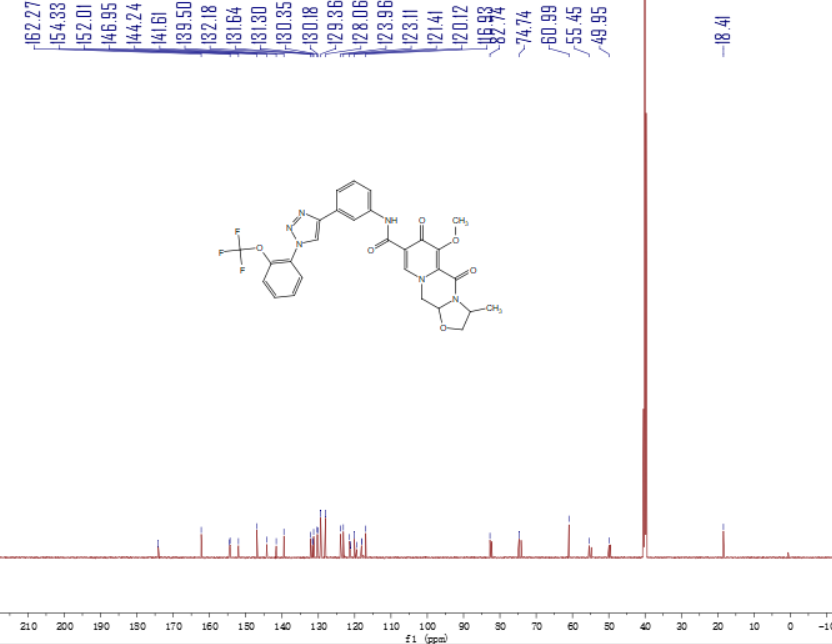


# Figure 10. ^1^H NMR and ^13^C NMR spectrums of compound KJ10


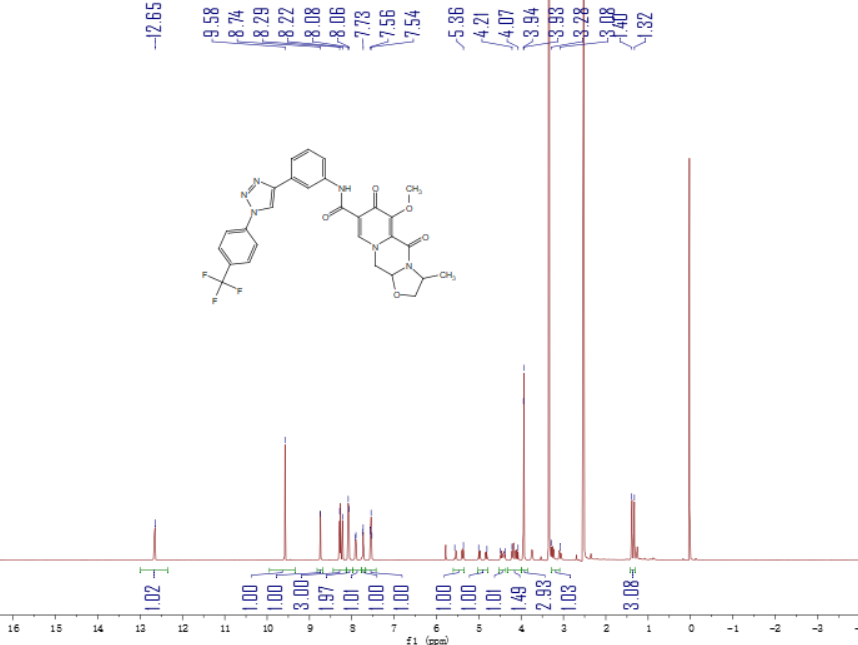


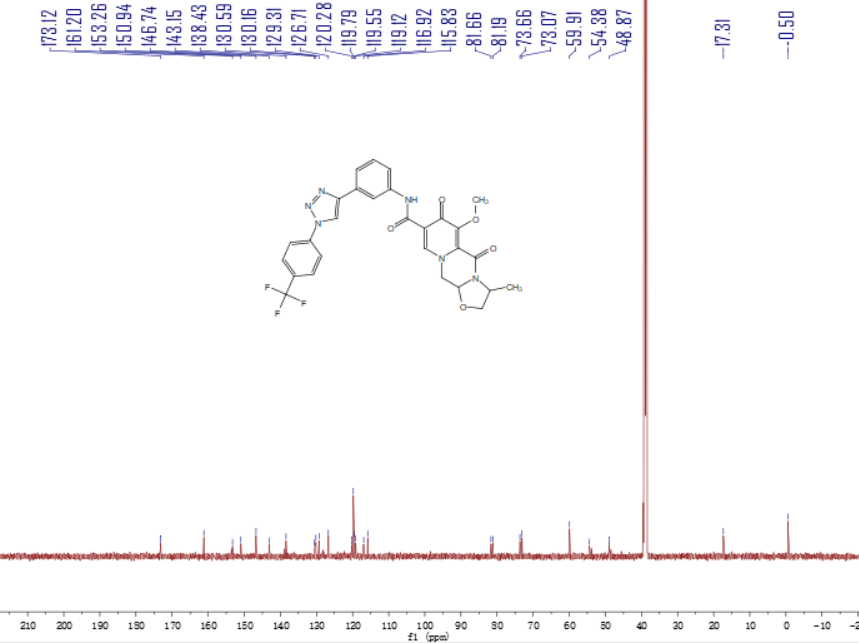


# Figure 11. ^1^H NMR and ^13^C NMR spectrums of compound KJ11


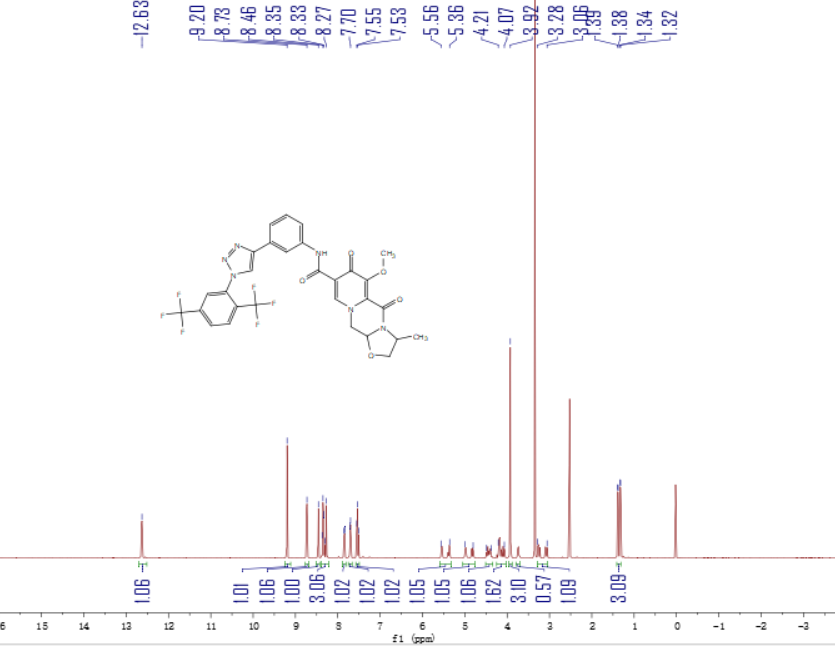


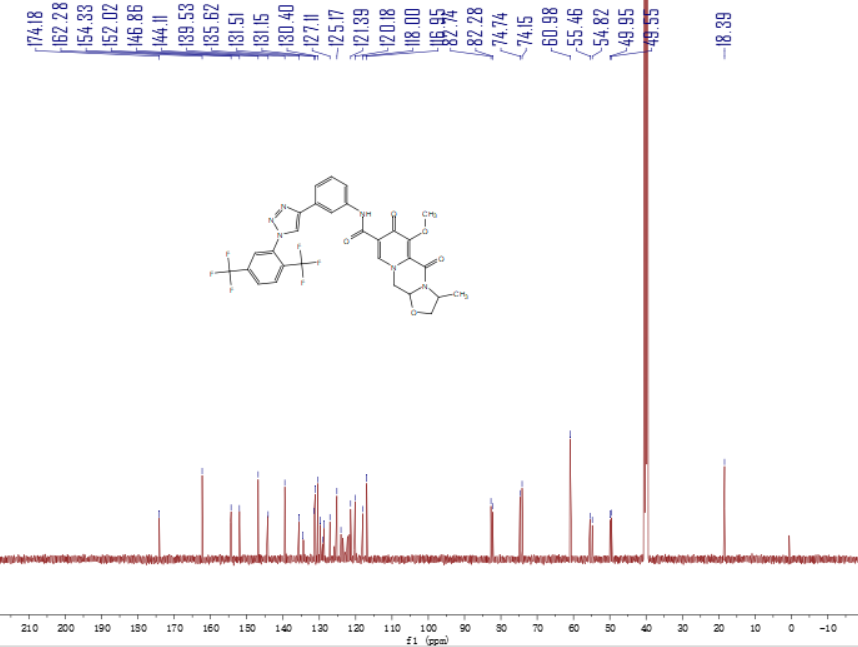


# Figure 12. ^1^H NMR and ^13^C NMR spectrums of compound KJ12


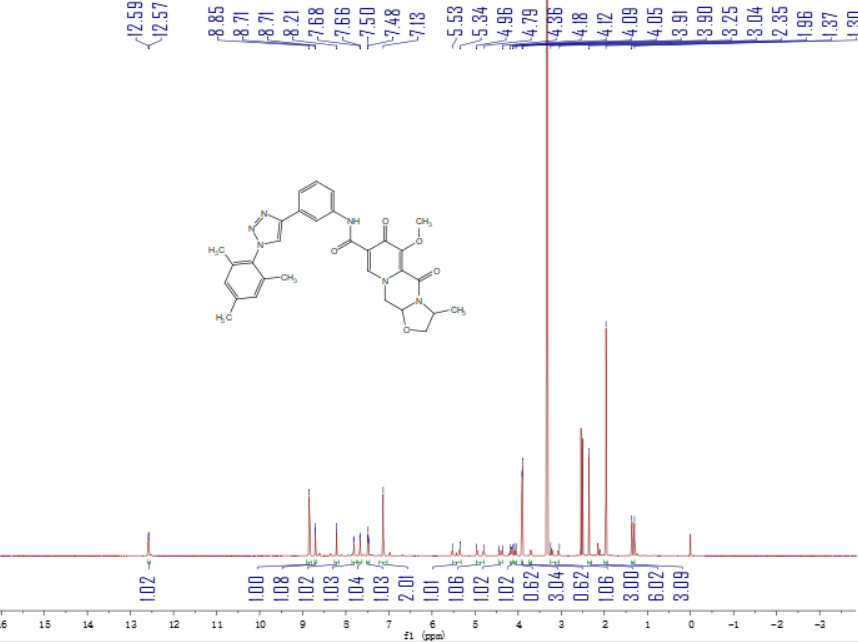


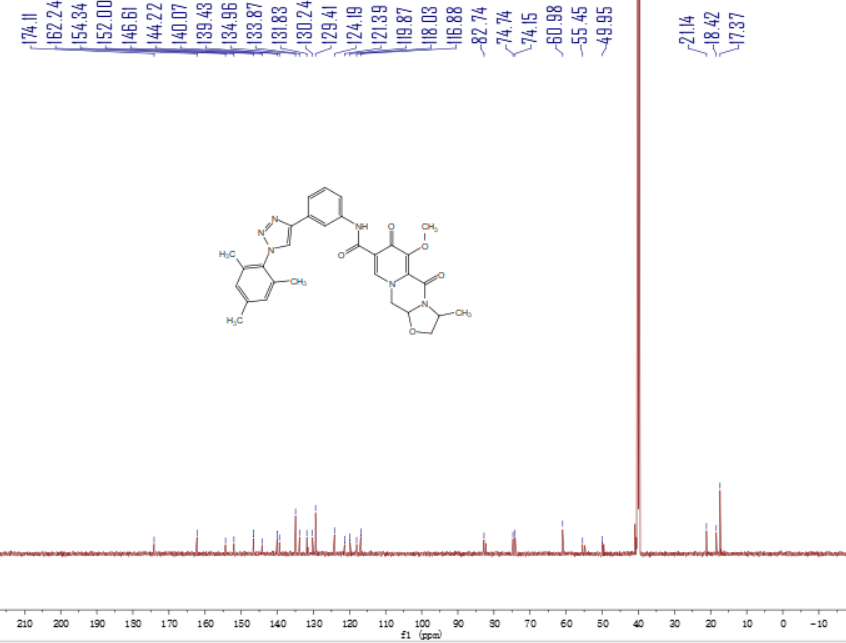


# Figure 13. ^1^H NMR and ^13^C NMR spectrums of compound KJ13


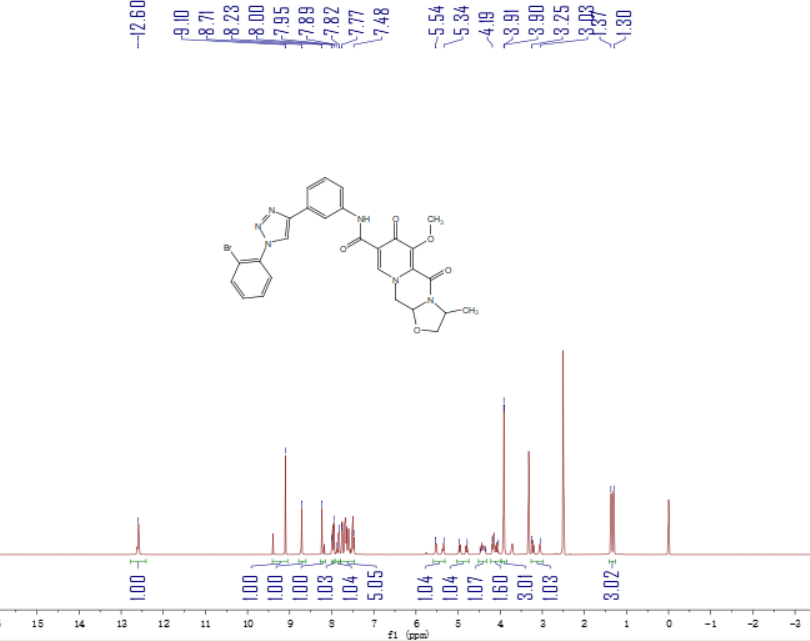


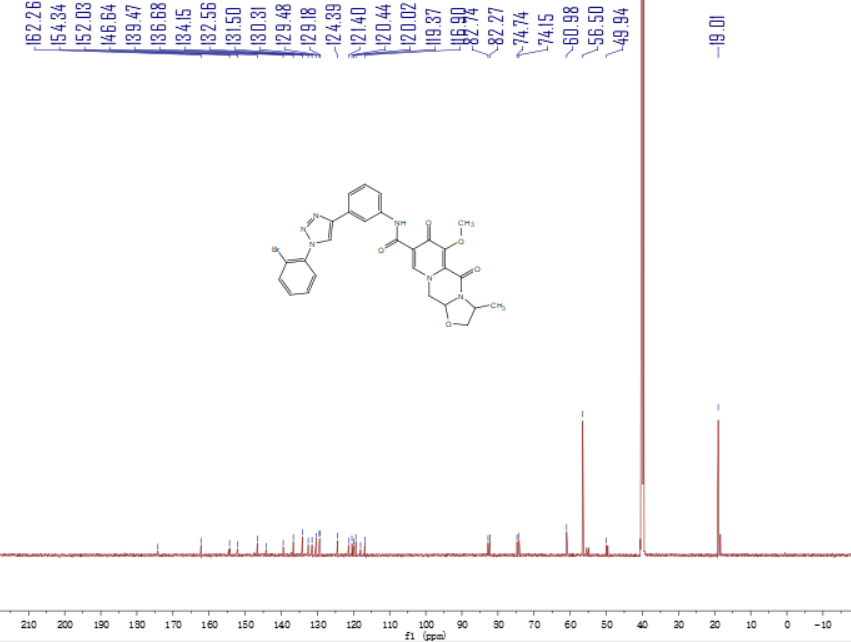


# Figure 14. ^1^H NMR and ^13^C NMR spectrums of compound KJ14


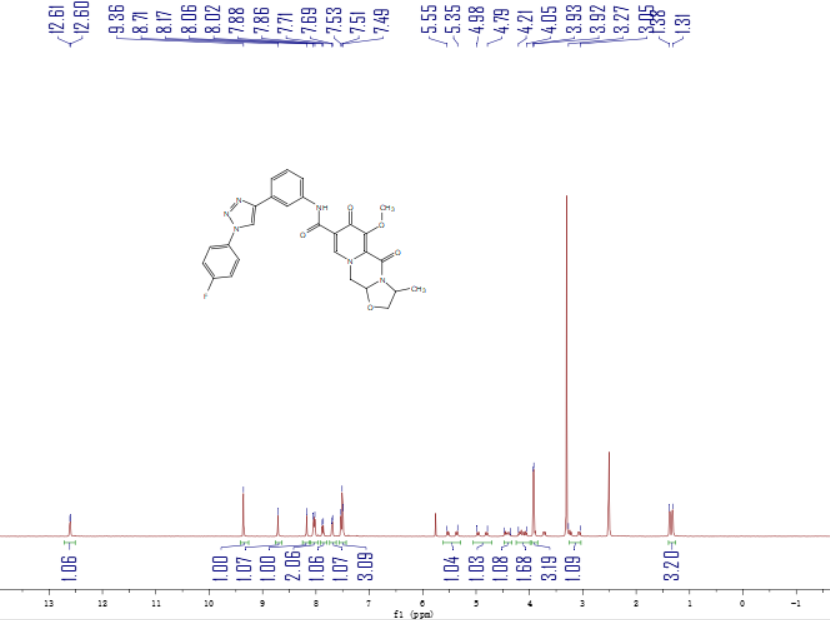


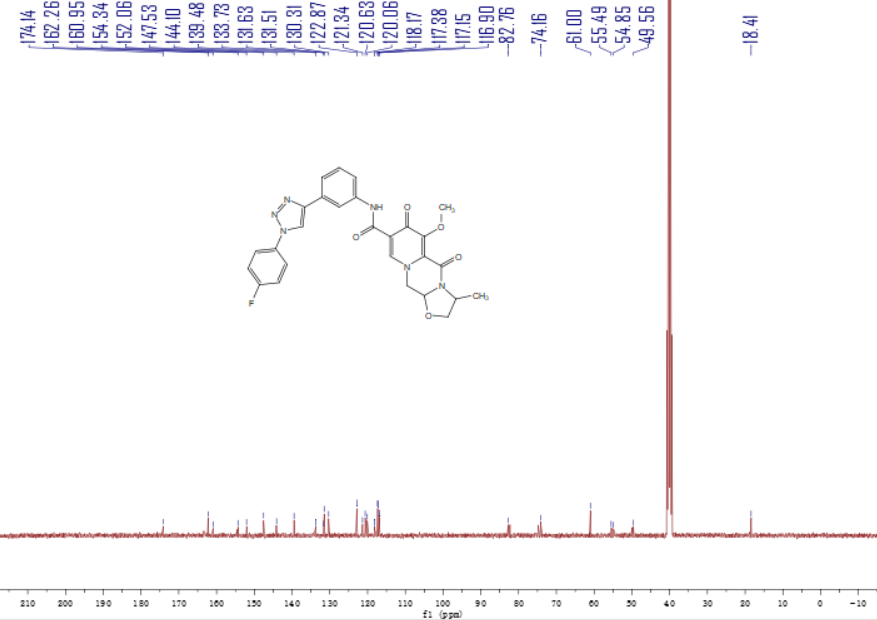


# Figure 15. ^1^H NMR and ^13^C NMR spectrums of compound KJ15


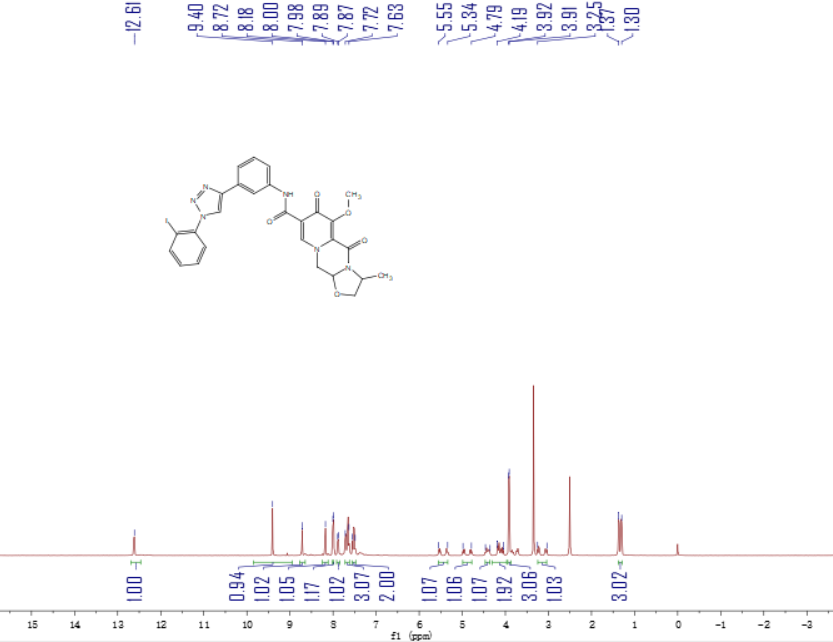


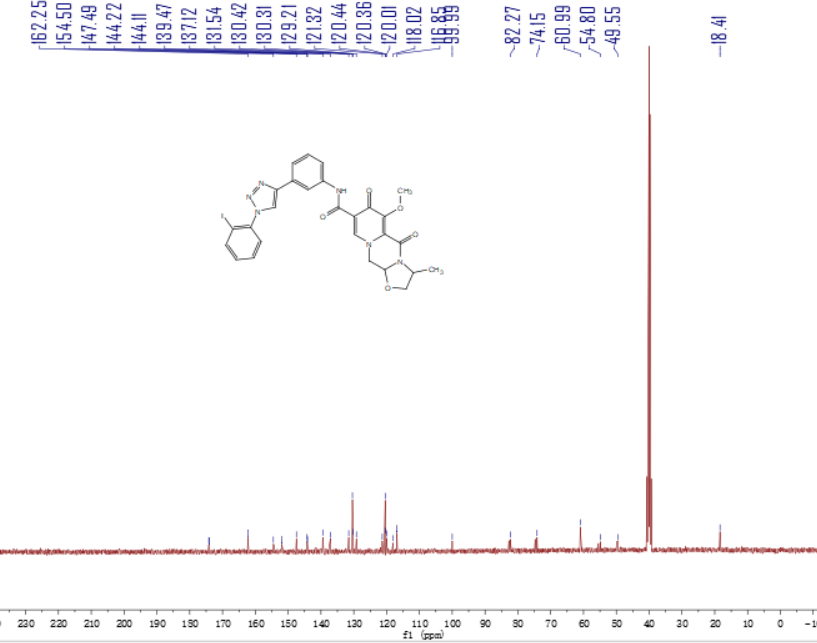


# Figure 16. ^1^H NMR and ^13^C NMR spectrums of compound KJ16


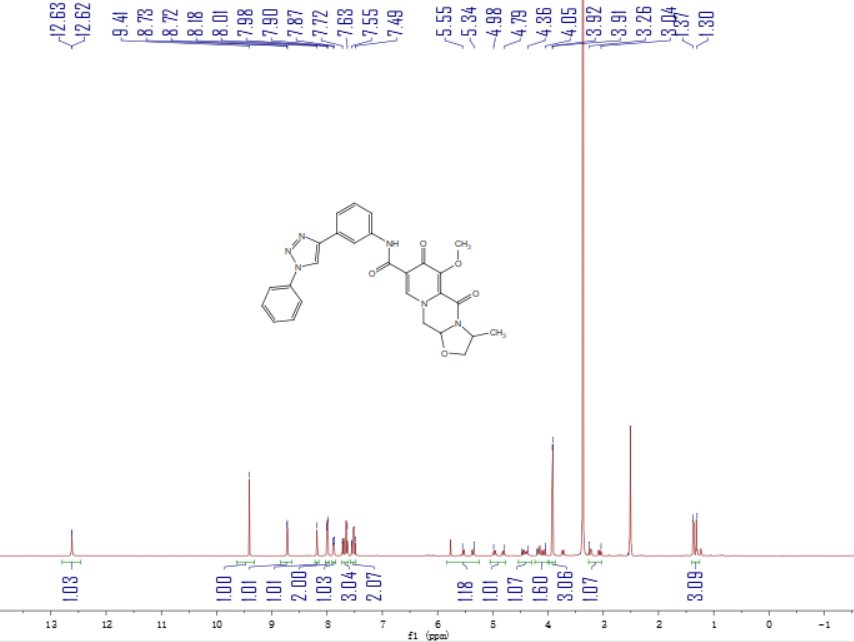


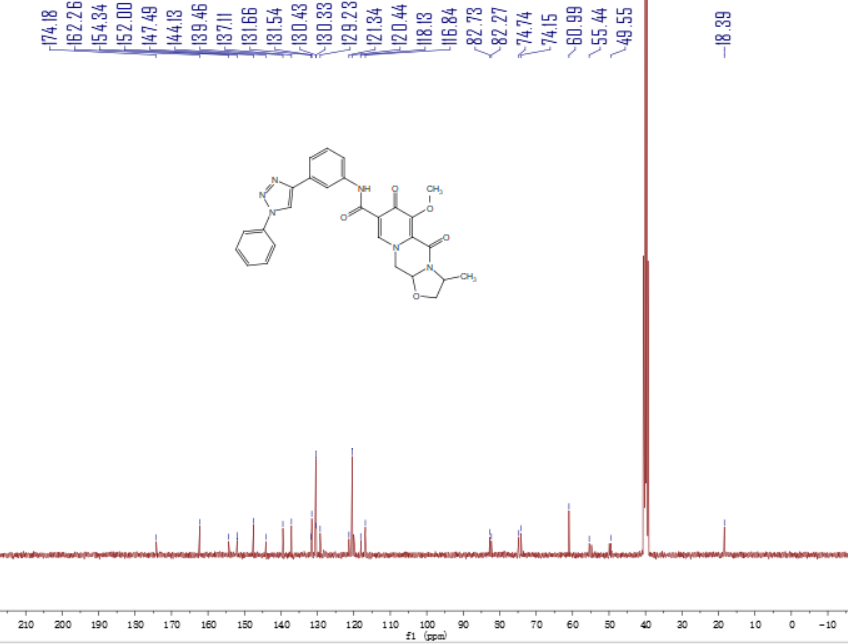


# Figure 17. ^1^H NMR and ^13^C NMR spectrums of compound KJ17


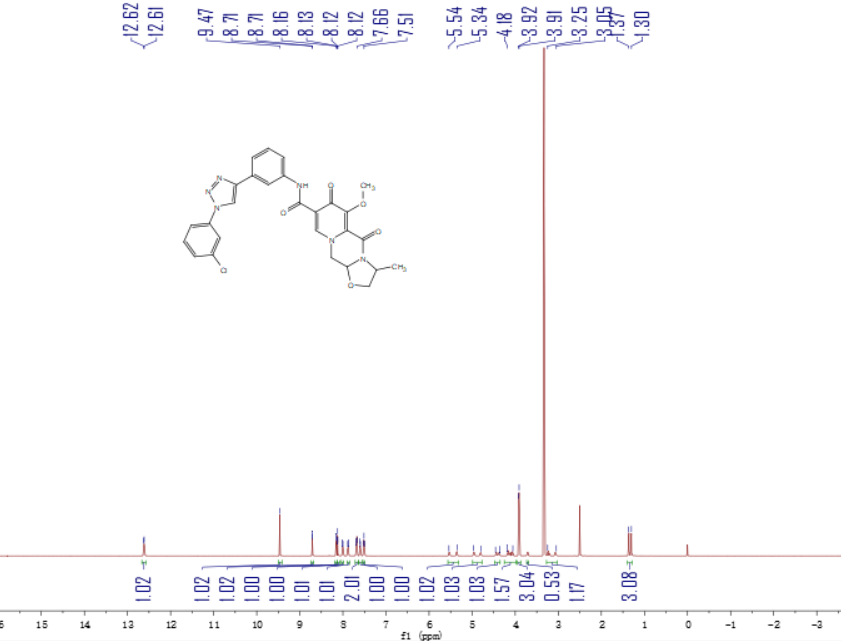


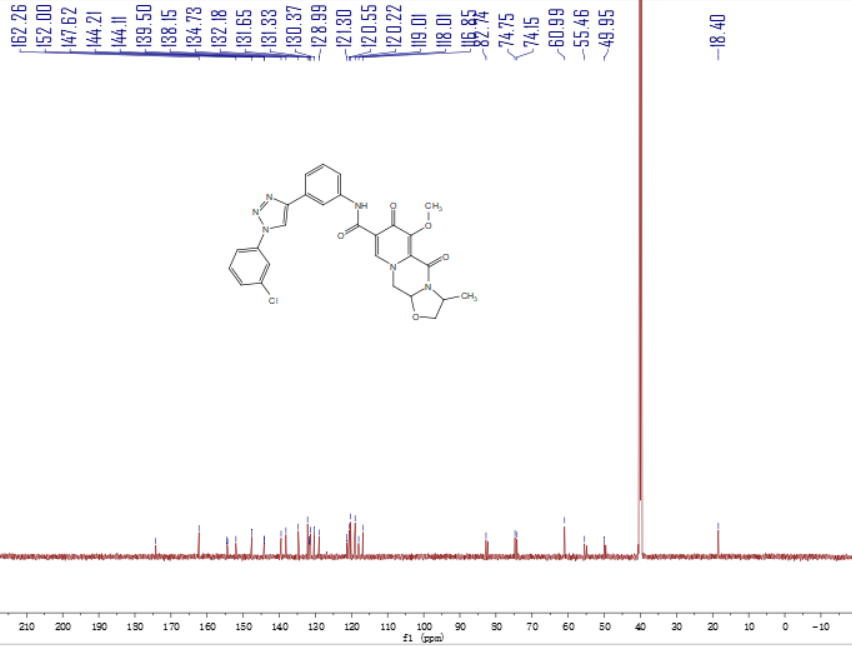


# Figure 18. ^1^H NMR and ^13^C NMR spectrums of compound KJ18


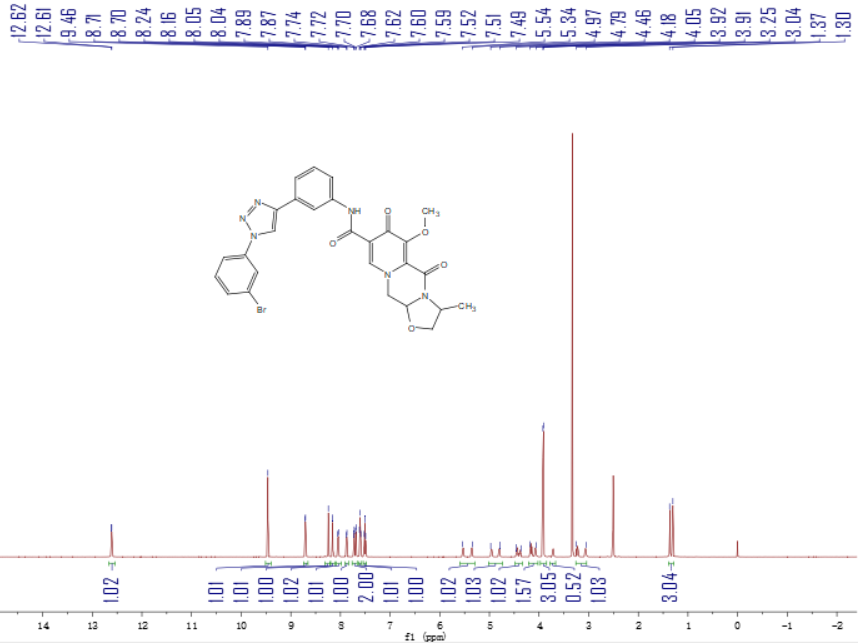


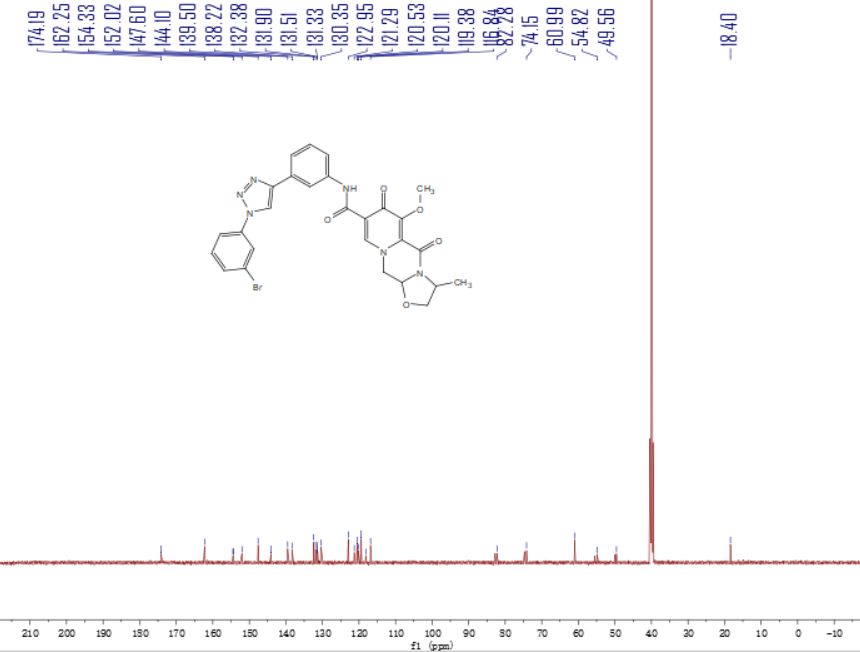


# Figure 19. ^1^H NMR and ^13^C NMR spectrums of compound KJ19


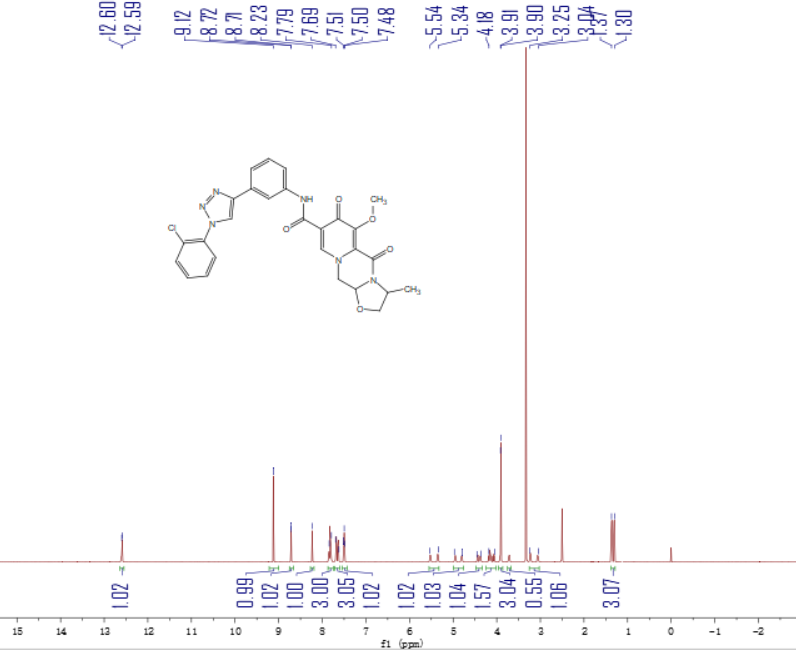


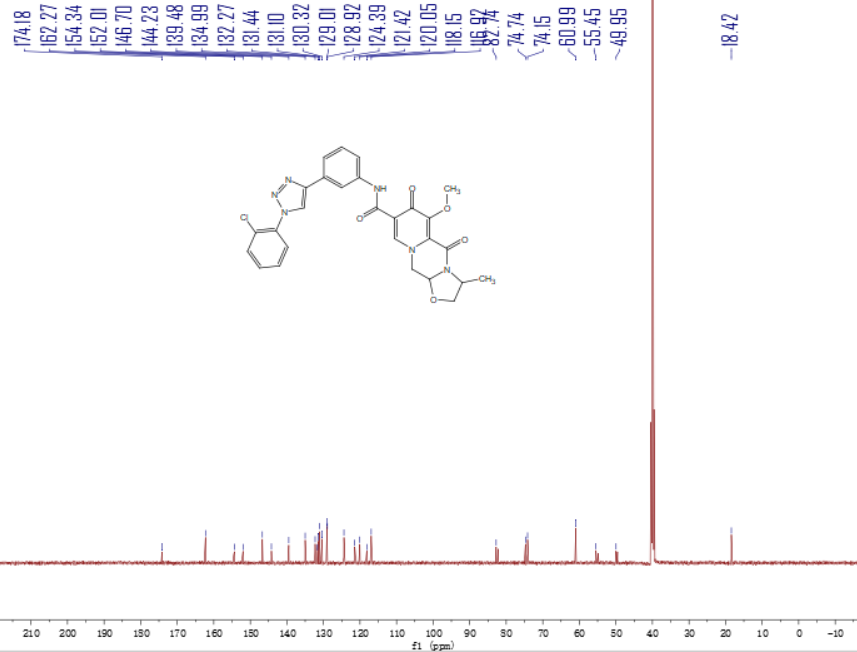

Supplement: Supplementary file 1 [file DataSheet1.zip › supplementary experimental data/4.The data of 1H NMR and 13C NMR spectrums.docx]
