# Supplementary figures and images for: Design and synthesis of cabotegravir derivatives bearing 1,2,3-triazole and evaluation of anti-liver cancer activity
Source: Front Pharmacol. 2023 Oct 6;14:1265289. doi: 10.3389/fphar.2023.1265289 (PMC10590056; doi:10.3389/fphar.2023.1265289)

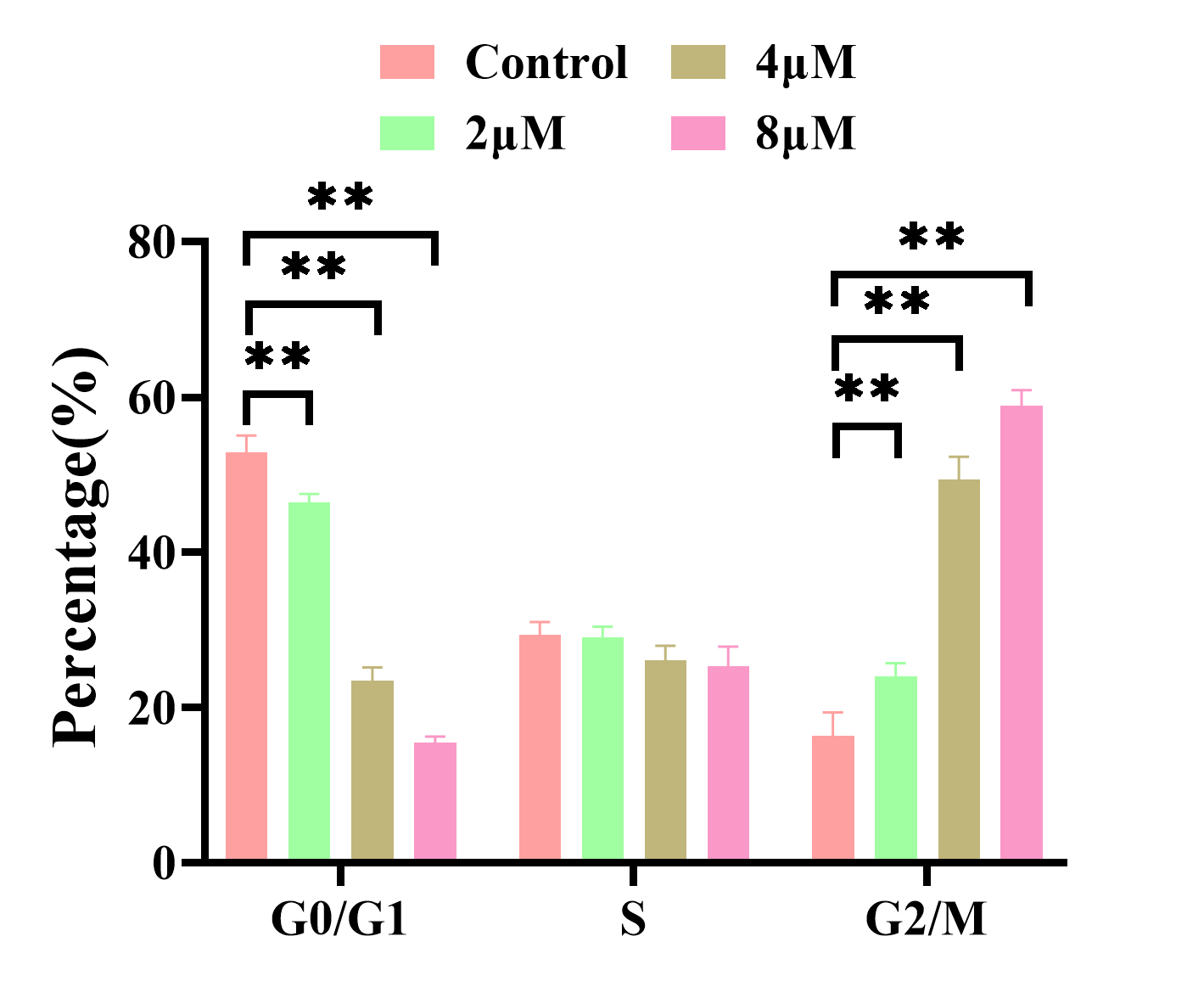

Supplement: Supplementary file 1 [file DataSheet1.zip › supplementary experimental data/2.Cell cycle/KJ-12.tif]

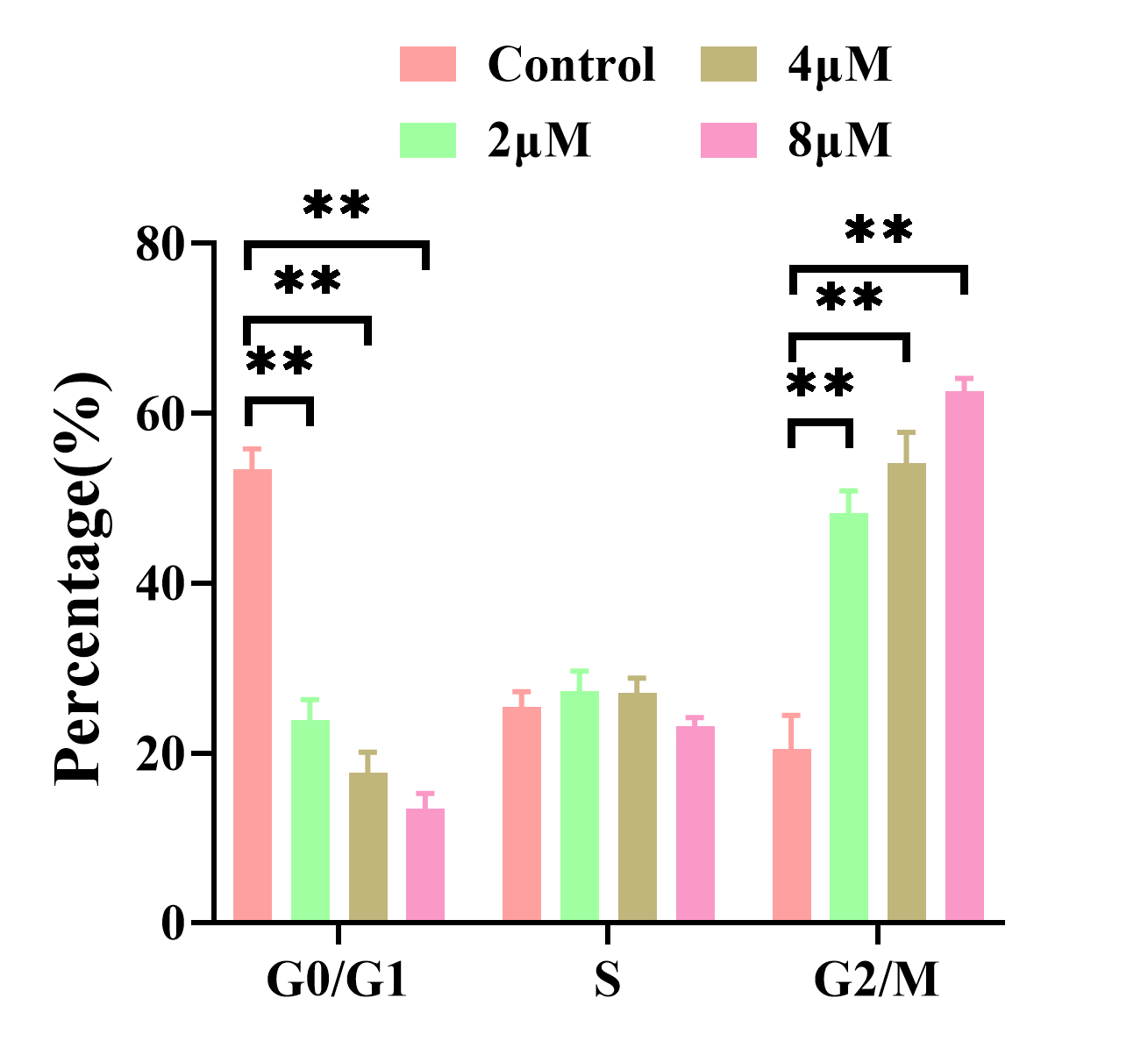

Supplement: Supplementary file 1 [file DataSheet1.zip › supplementary experimental data/2.Cell cycle/KJ-5.tif]

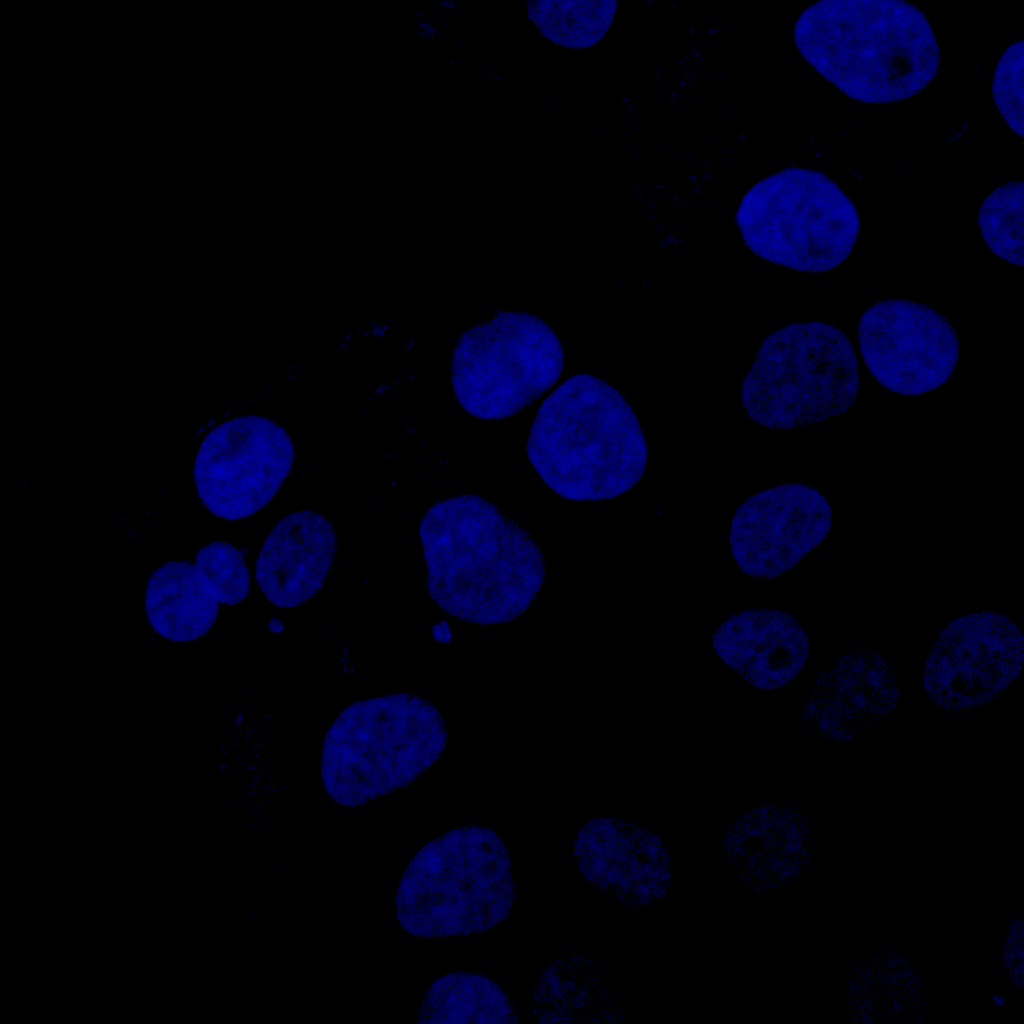

Supplement: Supplementary file 1 [file DataSheet1.zip › supplementary experimental data/3.p-H2AX/KJ-12/2-DAPI.tif]

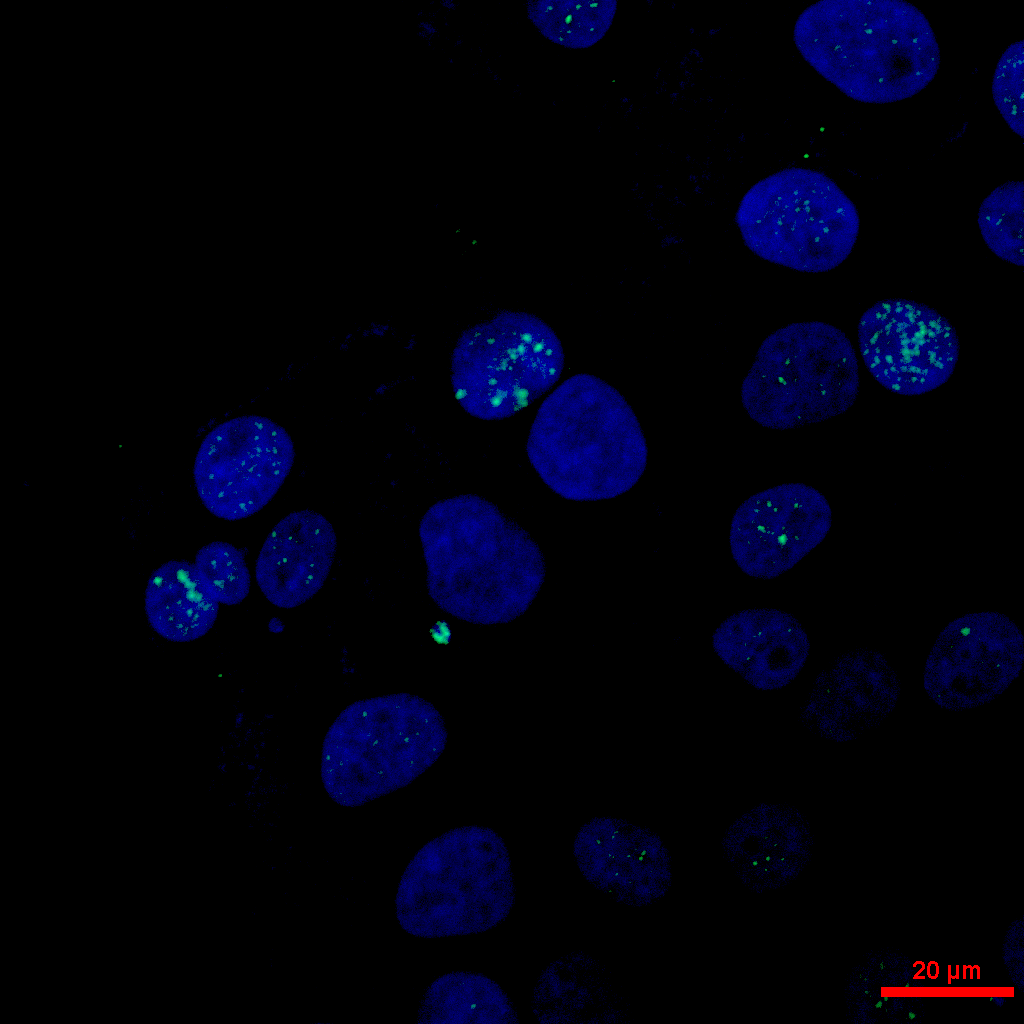

Supplement: Supplementary file 1 [file DataSheet1.zip › supplementary experimental data/3.p-H2AX/KJ-12/2-Merge.tif]

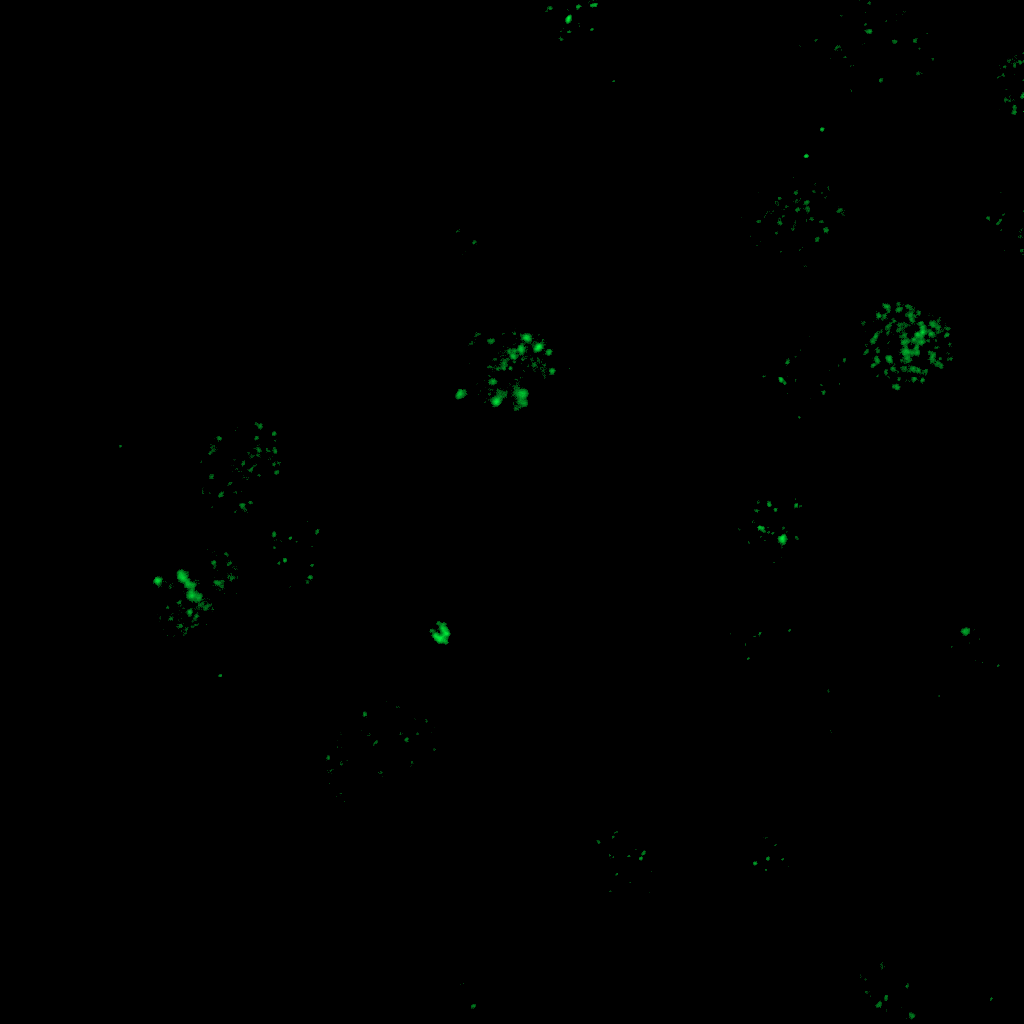

Supplement: Supplementary file 1 [file DataSheet1.zip › supplementary experimental data/3.p-H2AX/KJ-12/2-p-H2AX.tif]

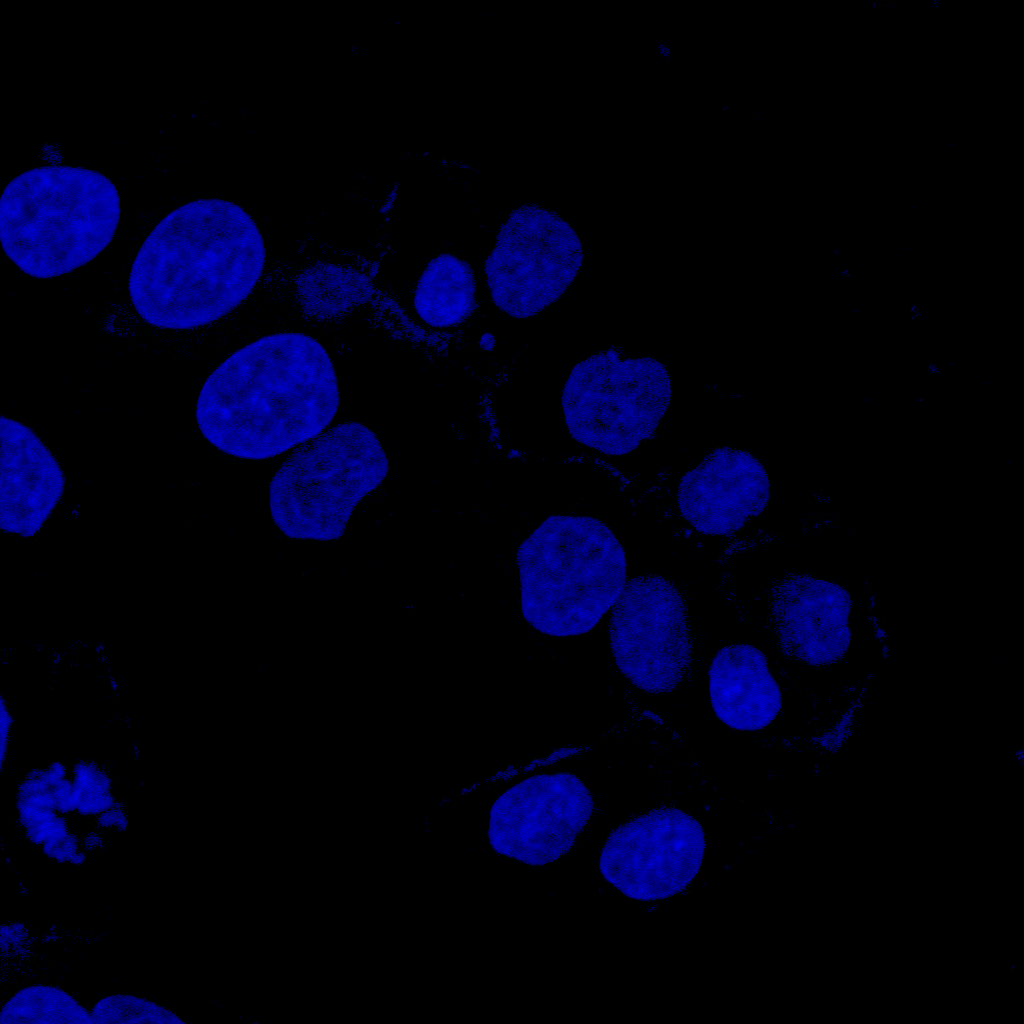

Supplement: Supplementary file 1 [file DataSheet1.zip › supplementary experimental data/3.p-H2AX/KJ-12/4-DAPI.tif]

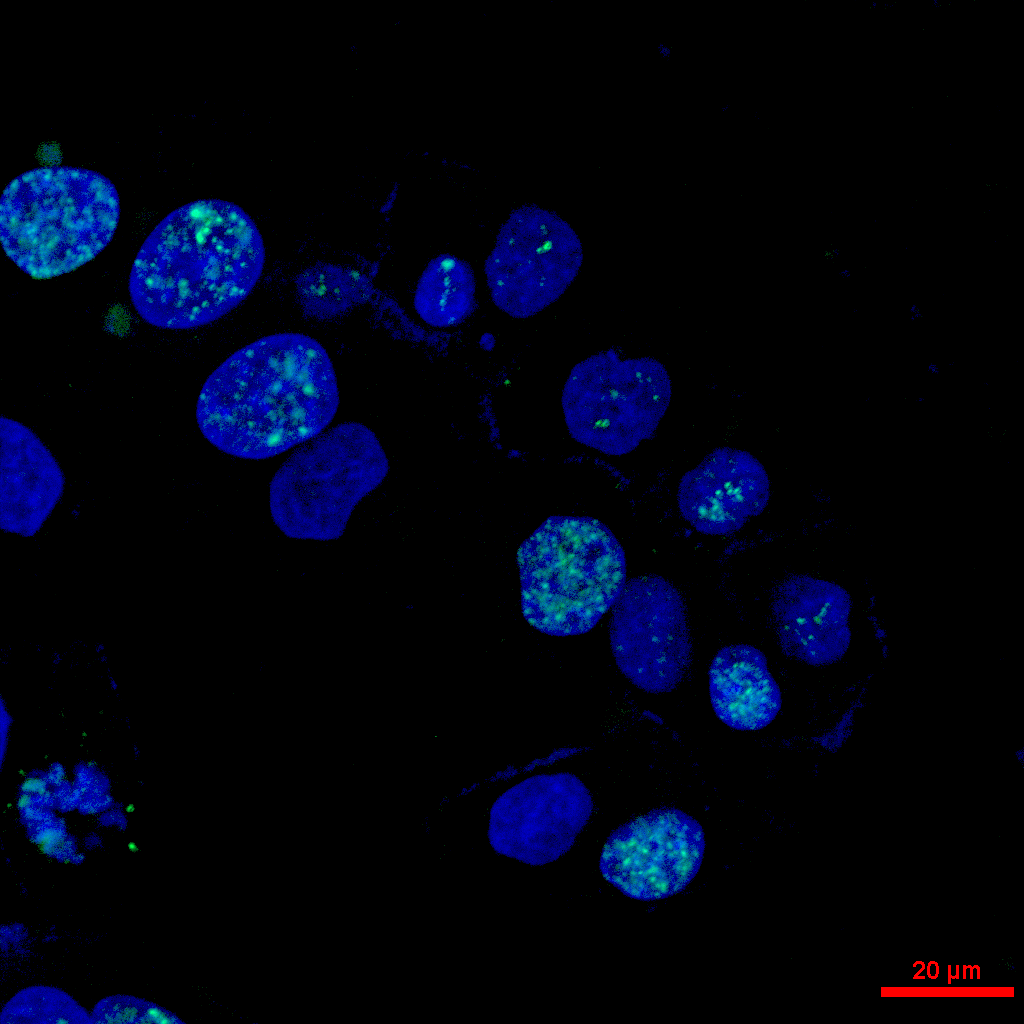

Supplement: Supplementary file 1 [file DataSheet1.zip › supplementary experimental data/3.p-H2AX/KJ-12/4-Merge.tif]

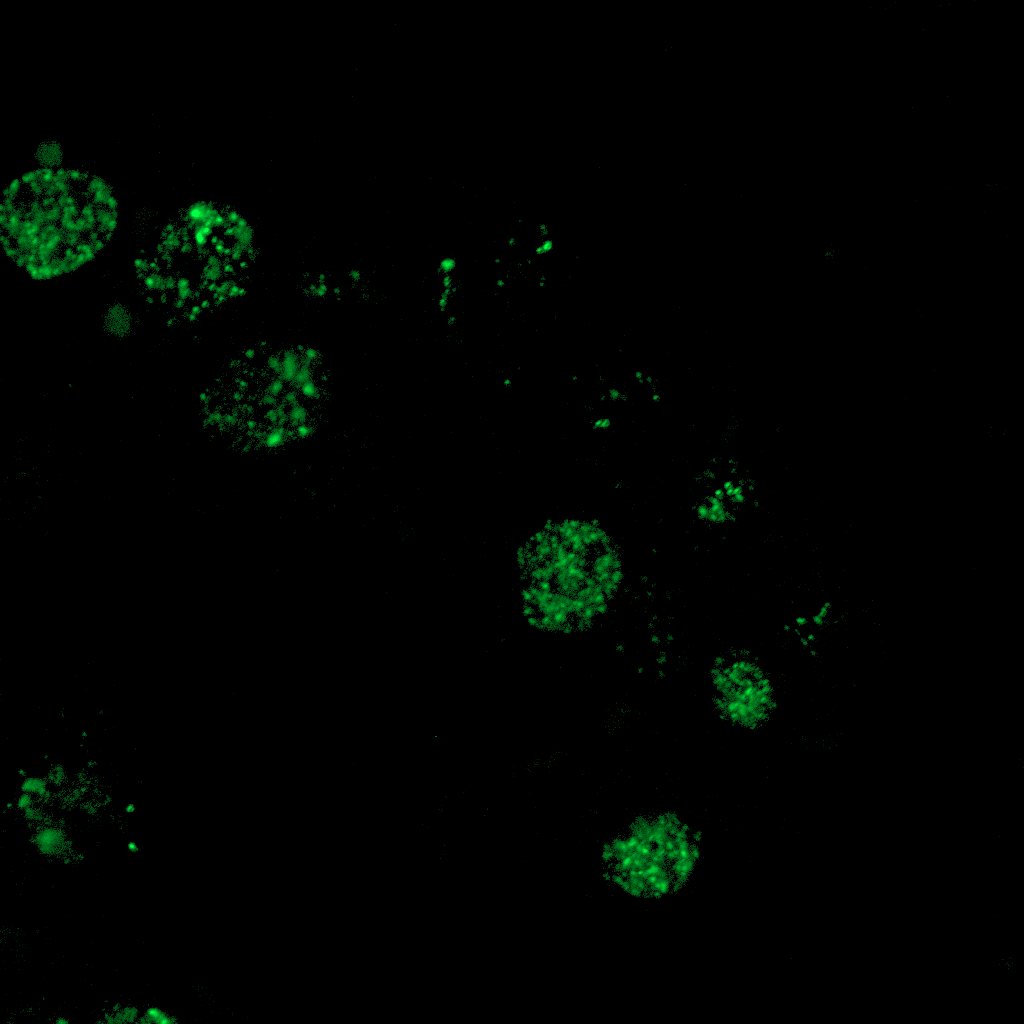

Supplement: Supplementary file 1 [file DataSheet1.zip › supplementary experimental data/3.p-H2AX/KJ-12/4-p-H2AX.tif]

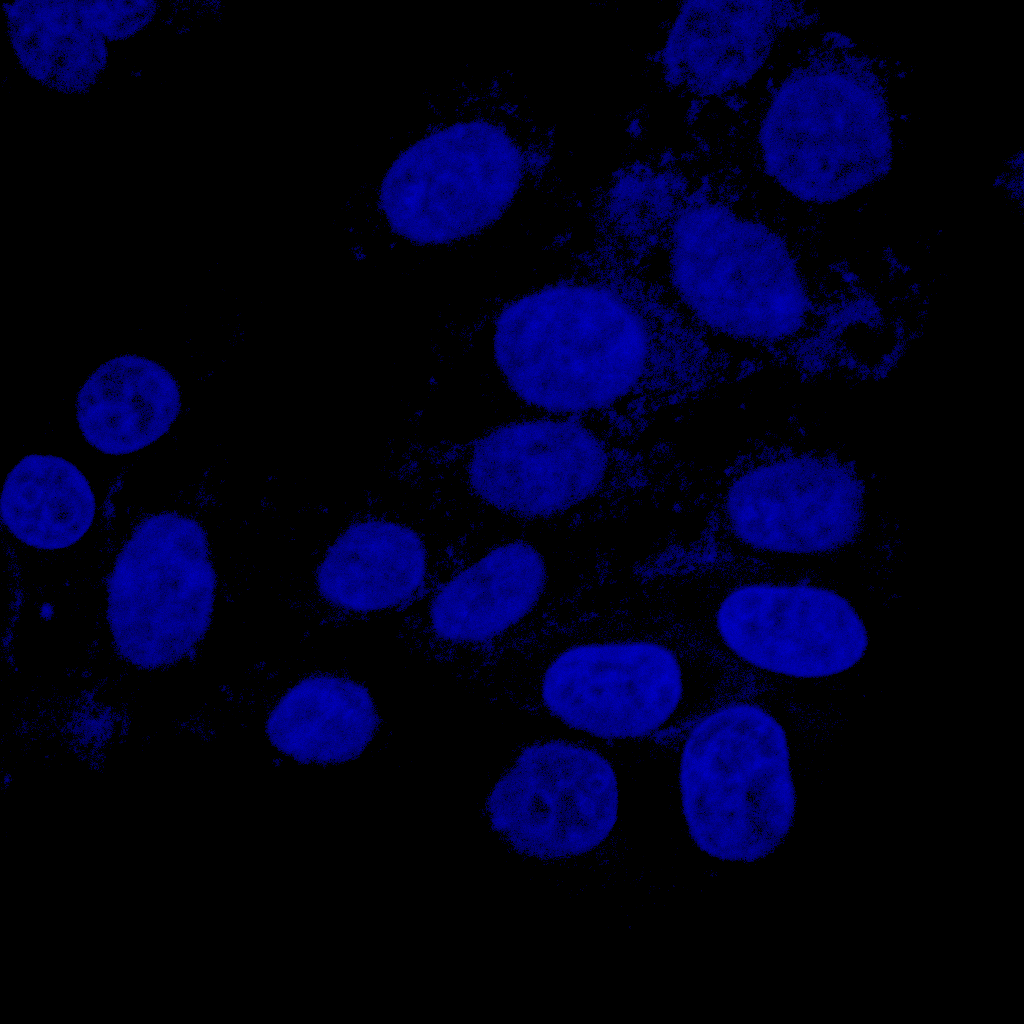

Supplement: Supplementary file 1 [file DataSheet1.zip › supplementary experimental data/3.p-H2AX/KJ-12/8-DAPI.tif]

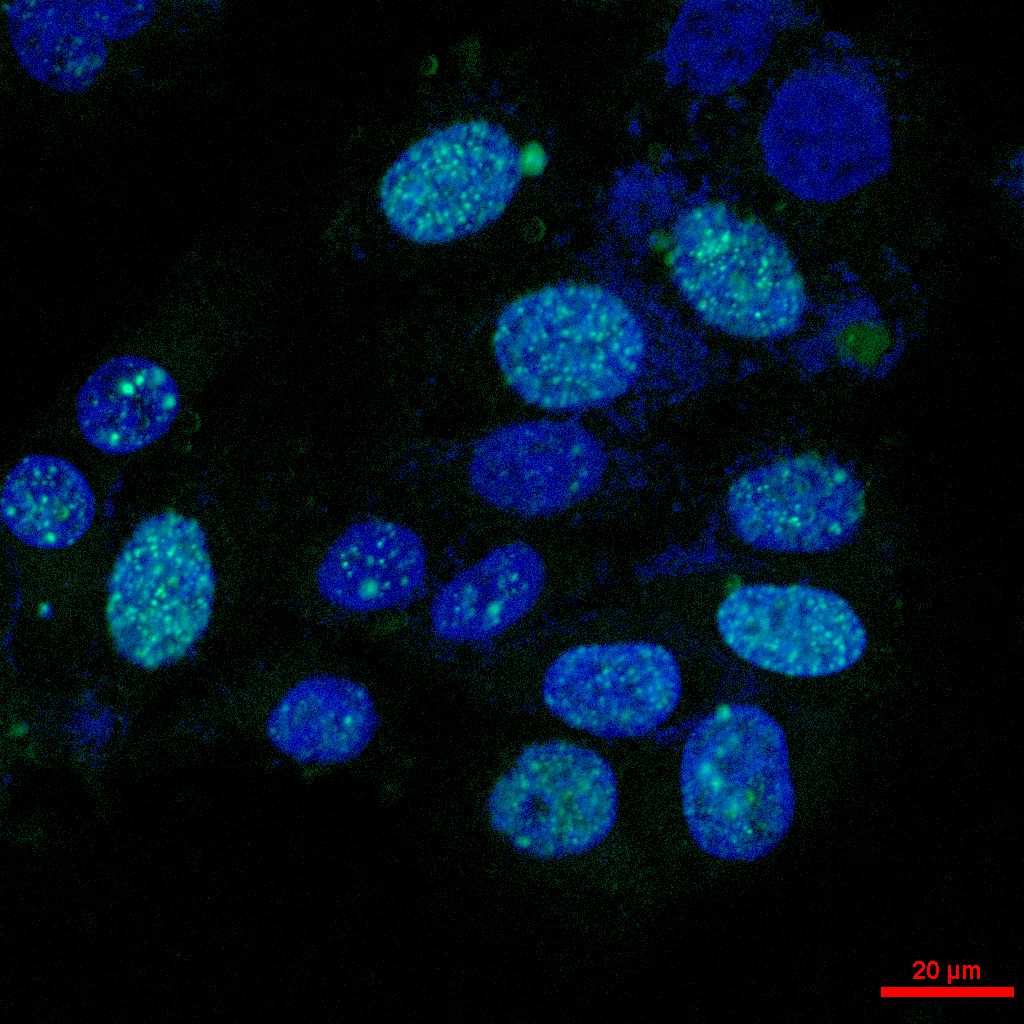

Supplement: Supplementary file 1 [file DataSheet1.zip › supplementary experimental data/3.p-H2AX/KJ-12/8-Merge.tif]

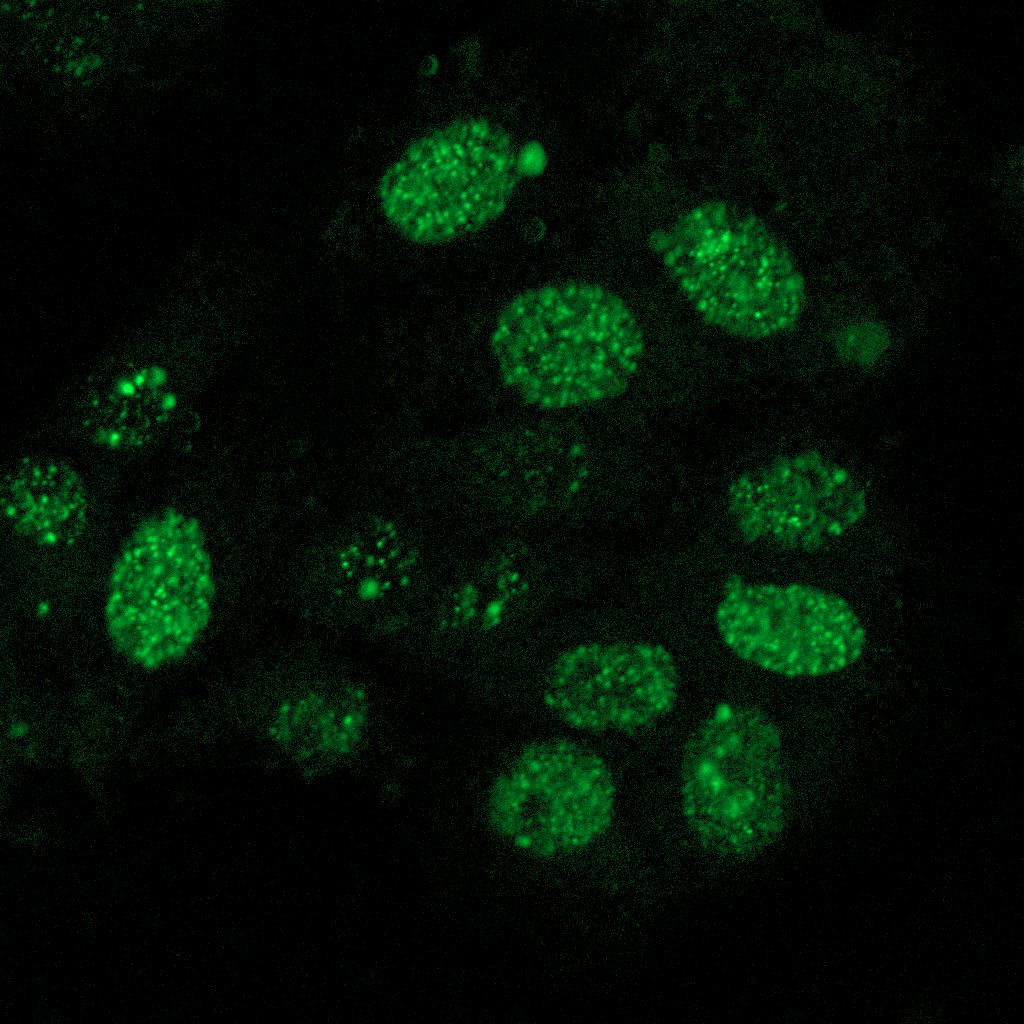

Supplement: Supplementary file 1 [file DataSheet1.zip › supplementary experimental data/3.p-H2AX/KJ-12/8-p-H2AX.tif]

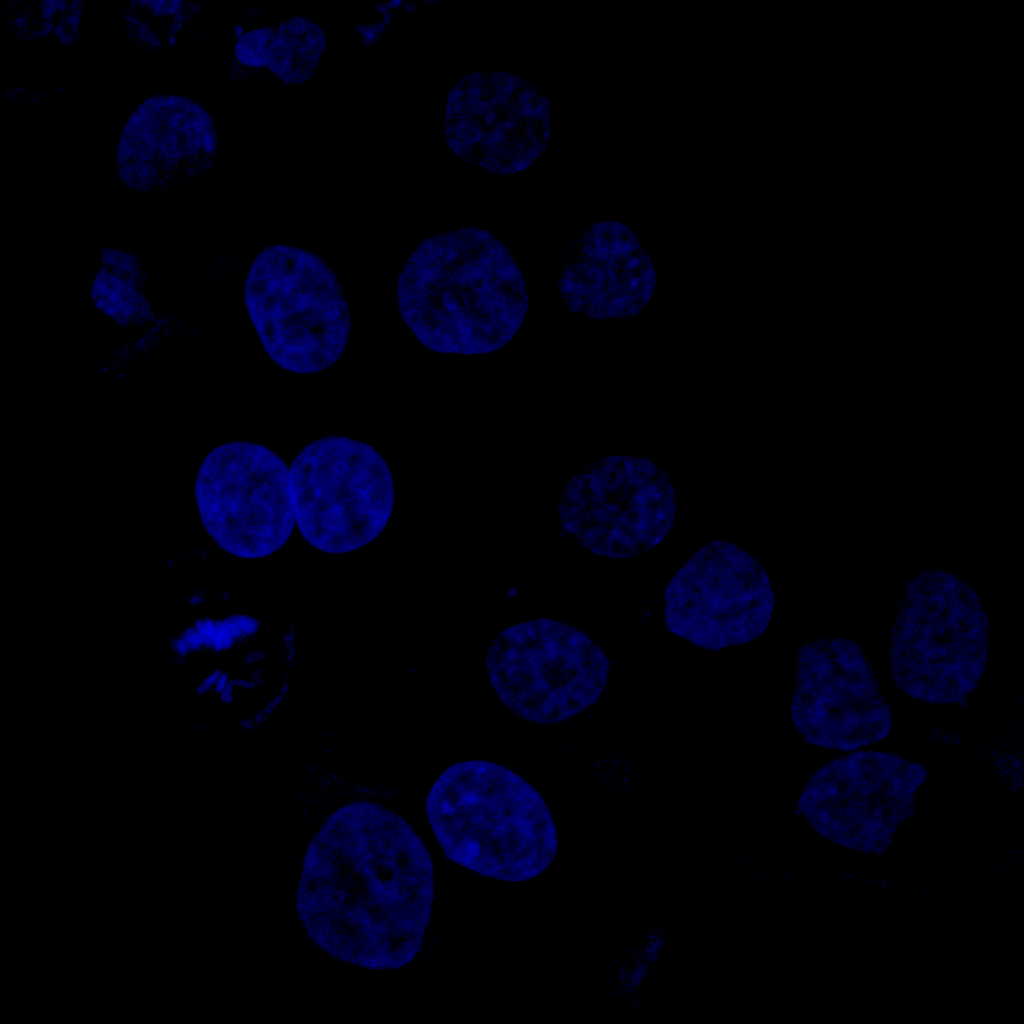

Supplement: Supplementary file 1 [file DataSheet1.zip › supplementary experimental data/3.p-H2AX/KJ-12/C-DAPI.tif]

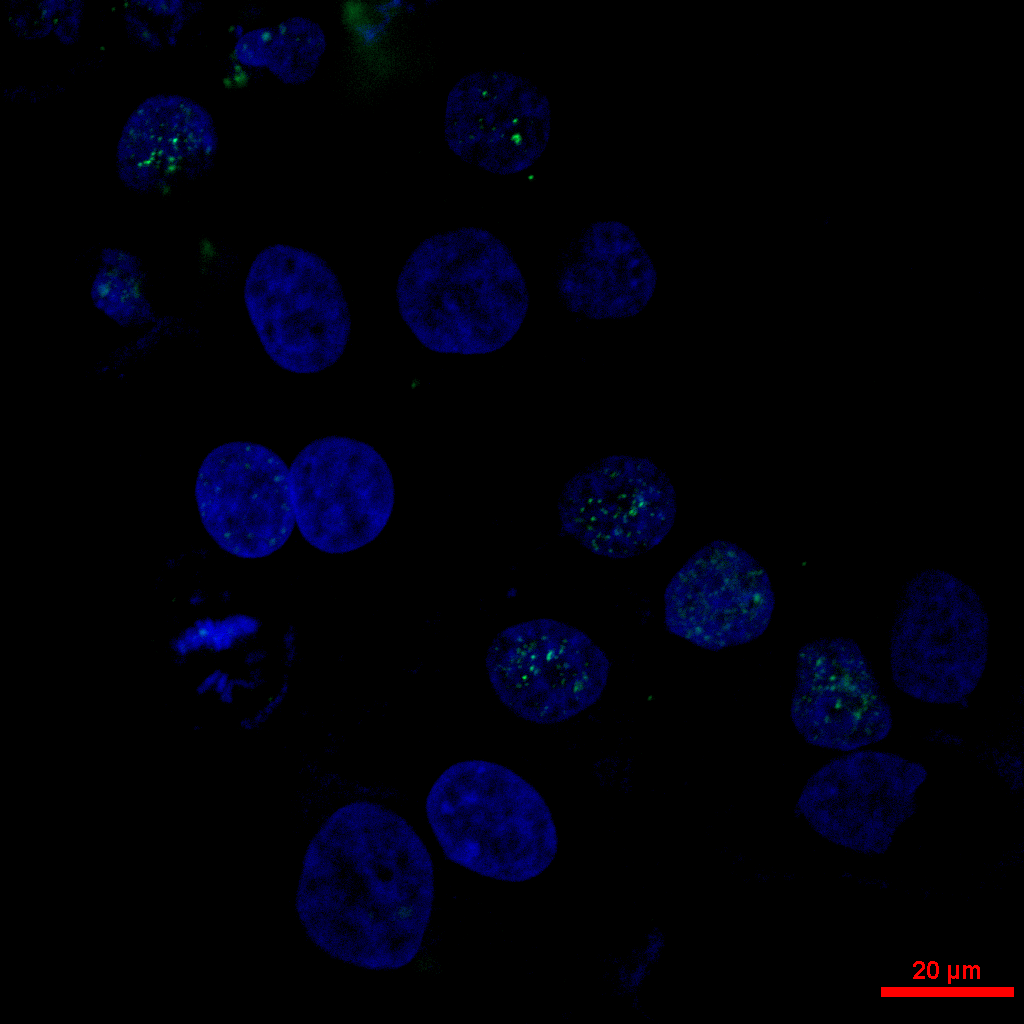

Supplement: Supplementary file 1 [file DataSheet1.zip › supplementary experimental data/3.p-H2AX/KJ-12/C-Merge.tif]

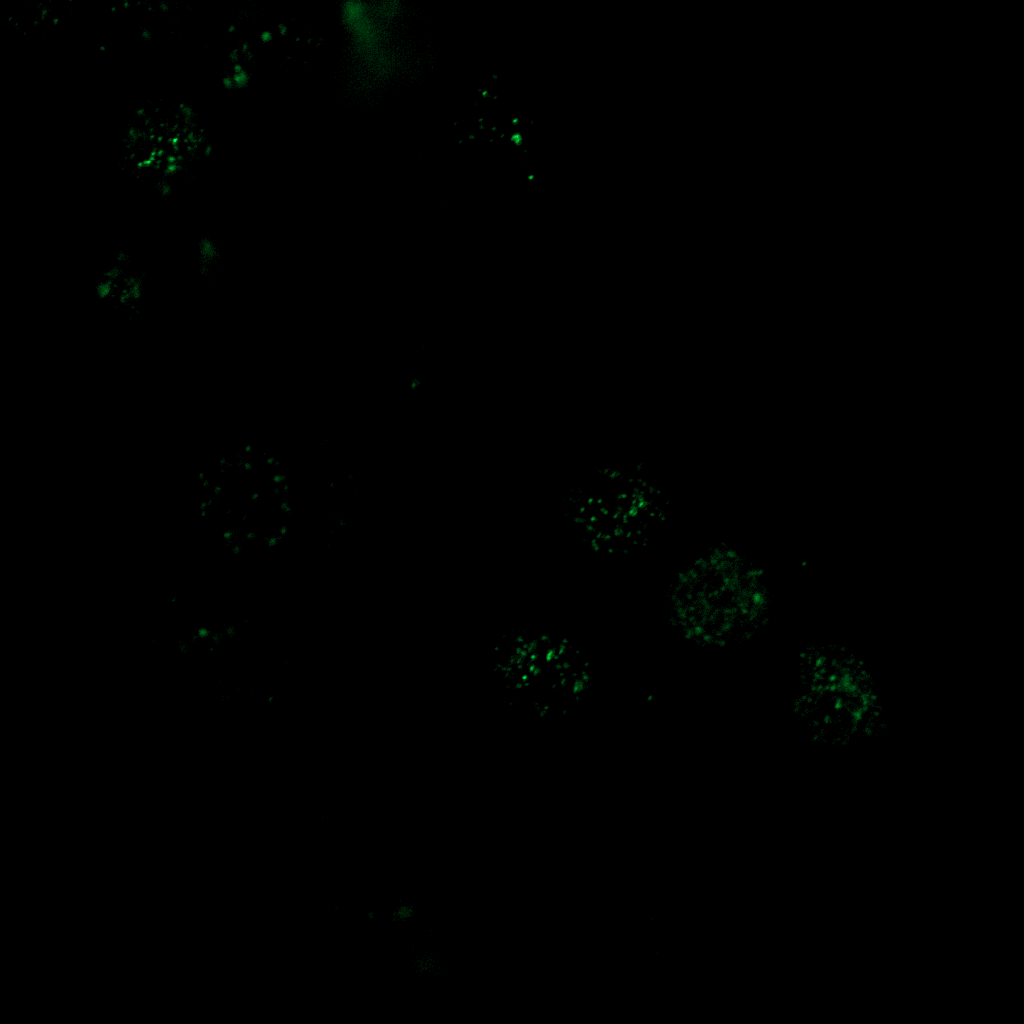

Supplement: Supplementary file 1 [file DataSheet1.zip › supplementary experimental data/3.p-H2AX/KJ-12/C-p-H2AX.tif]

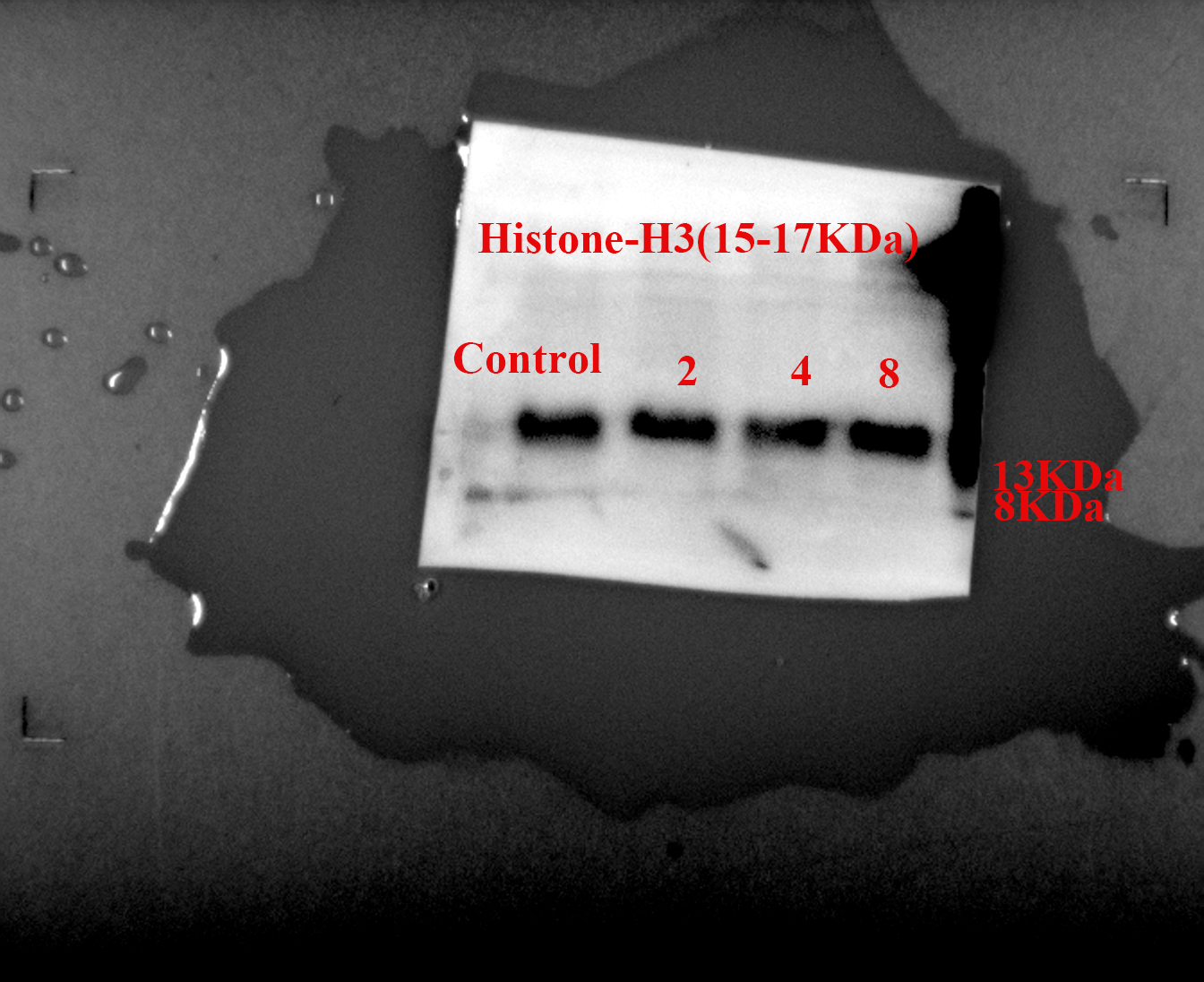

Supplement: Supplementary file 1 [file DataSheet1.zip › supplementary experimental data/3.p-H2AX/KJ-12/Histone-8.29-3-3 - ╕▒▒╛.tif]

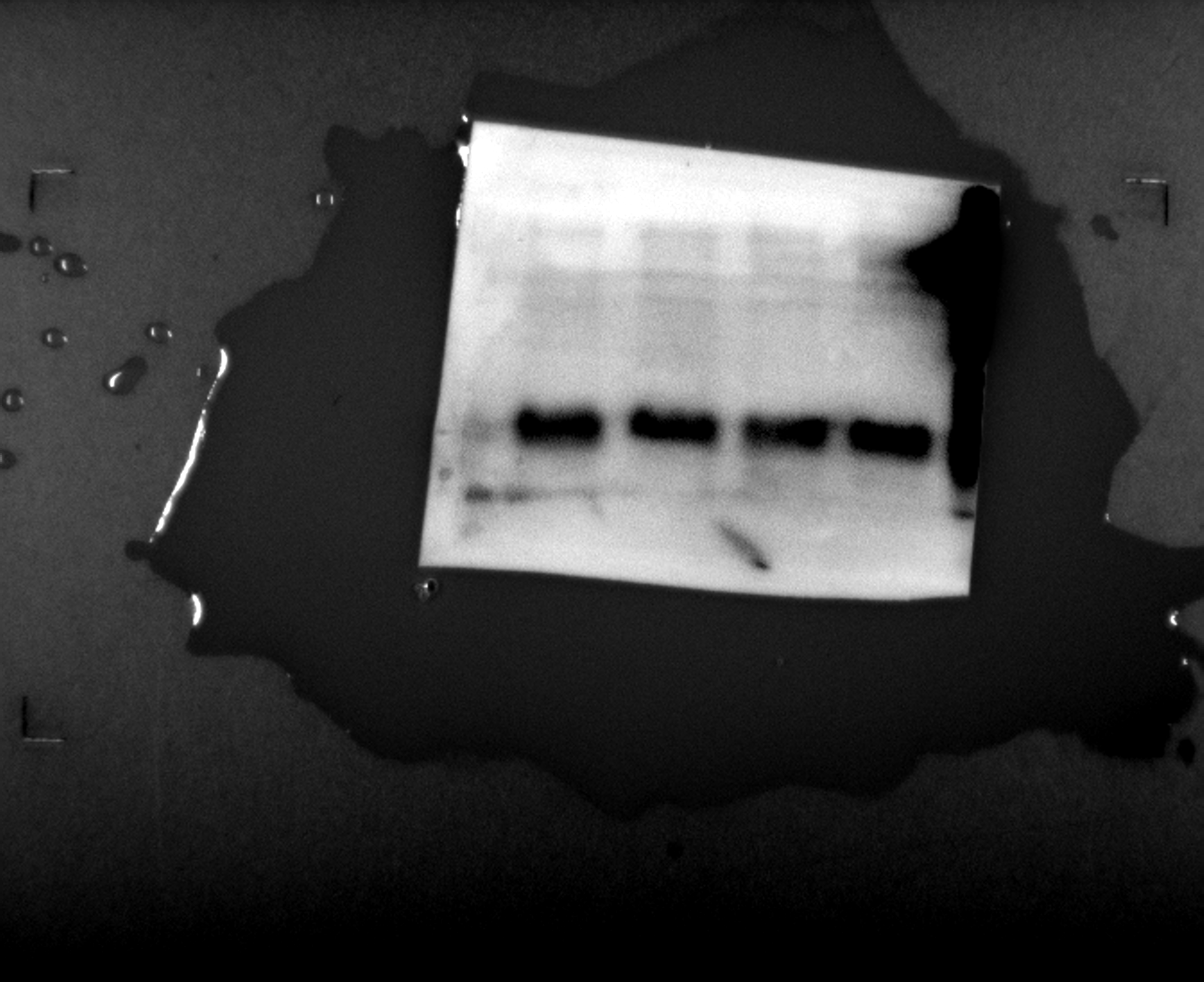

Supplement: Supplementary file 1 [file DataSheet1.zip › supplementary experimental data/3.p-H2AX/KJ-12/Histone-8.29-3-3.tif]

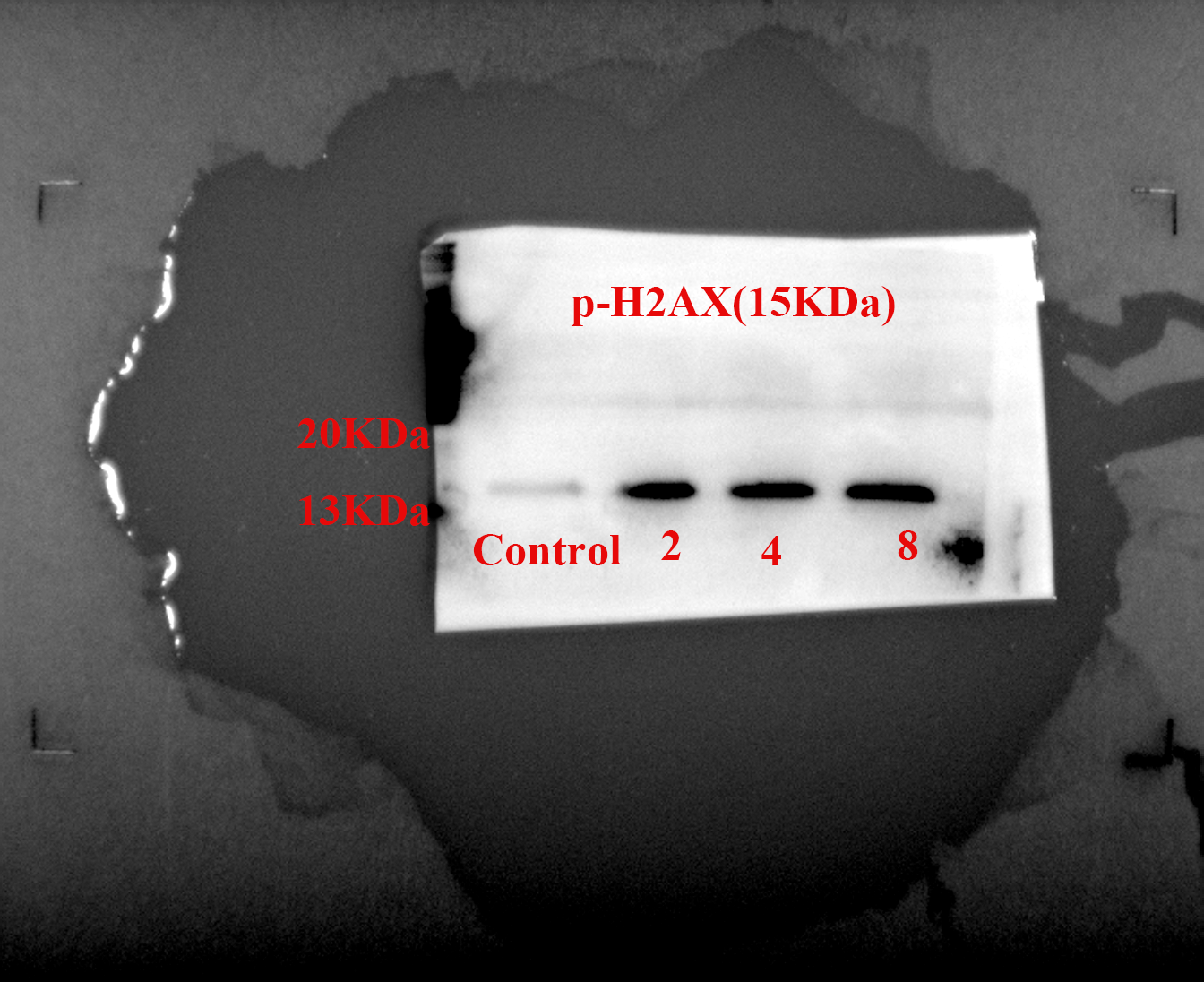

Supplement: Supplementary file 1 [file DataSheet1.zip › supplementary experimental data/3.p-H2AX/KJ-12/p-H2AX-2-4 - ╕▒▒╛.tif]

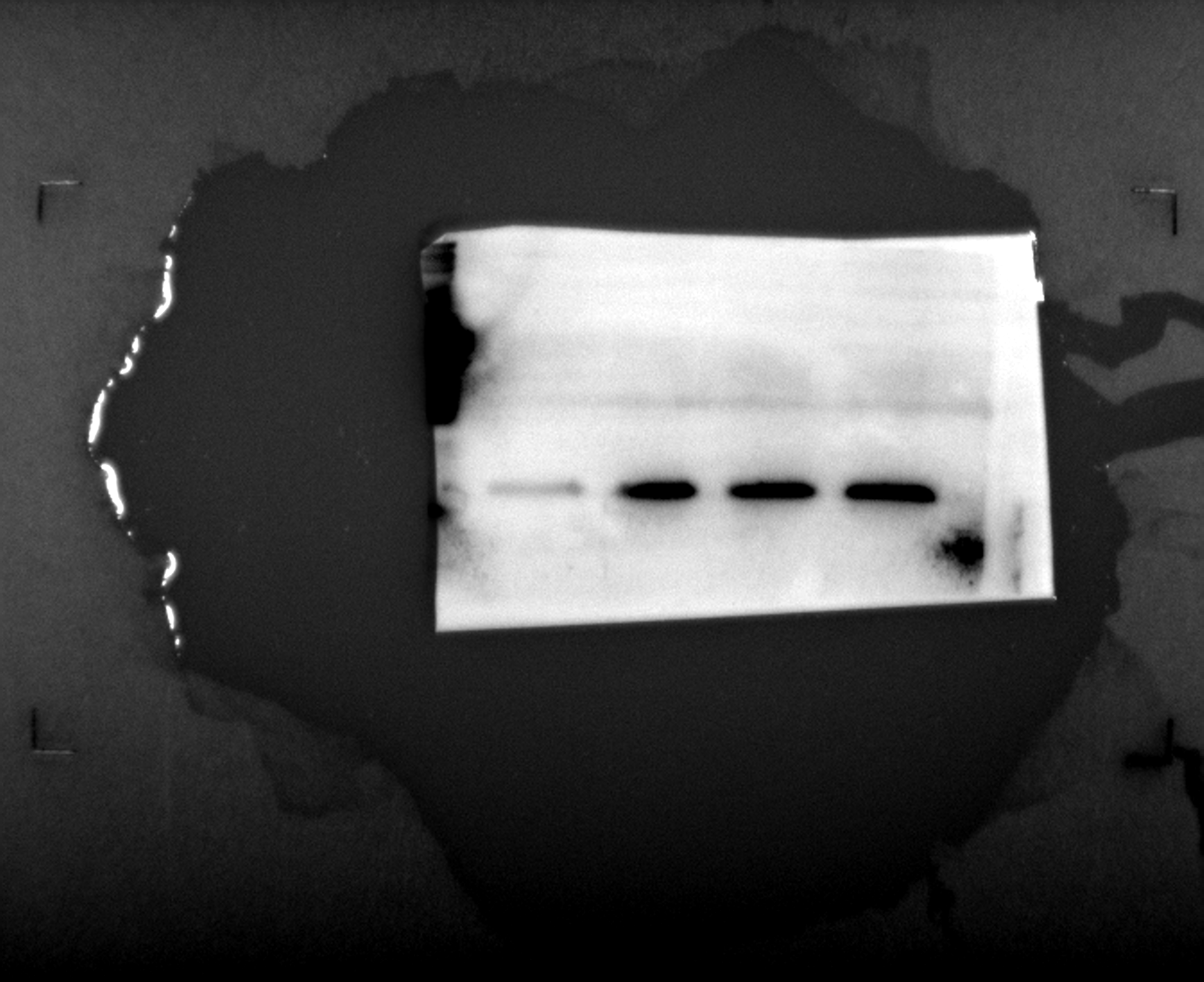

Supplement: Supplementary file 1 [file DataSheet1.zip › supplementary experimental data/3.p-H2AX/KJ-12/p-H2AX-2-4.tif]

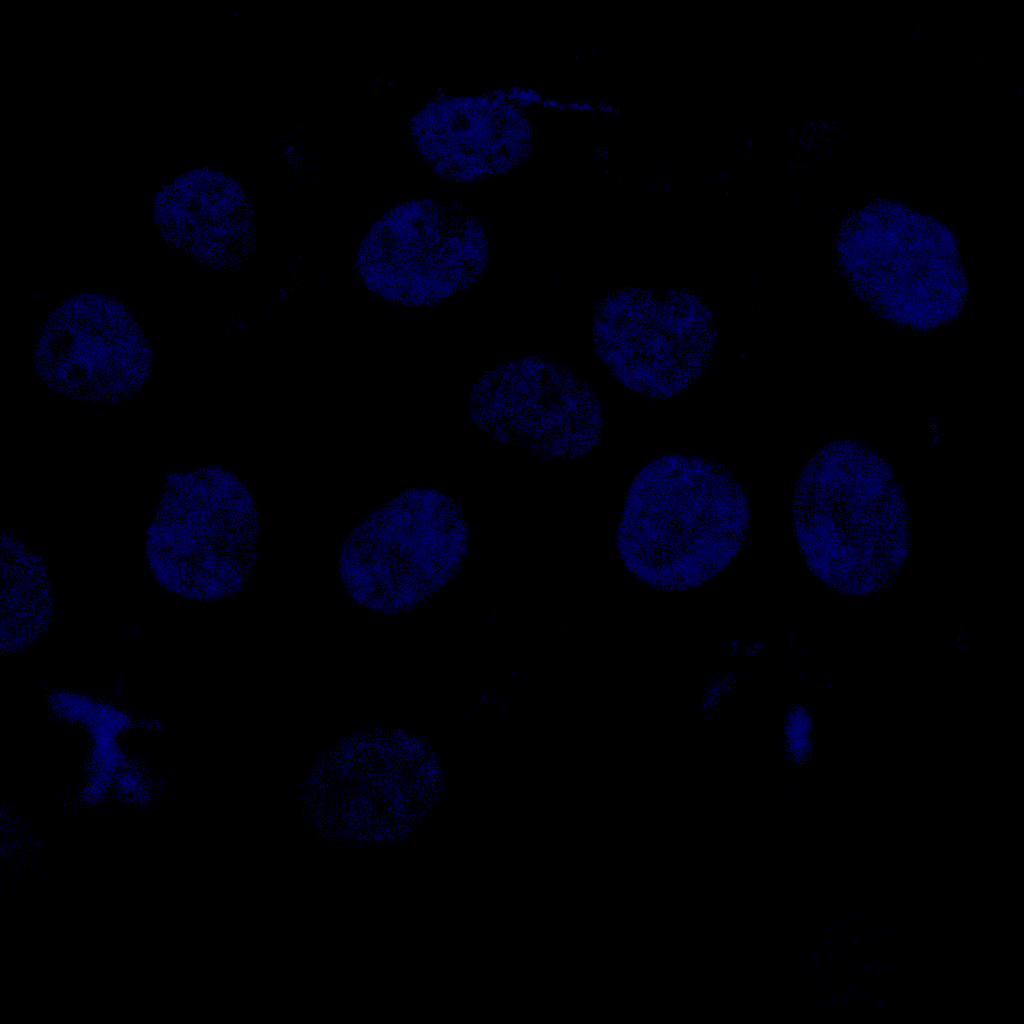

Supplement: Supplementary file 1 [file DataSheet1.zip › supplementary experimental data/3.p-H2AX/KJ-5/2-DAPI.tif]

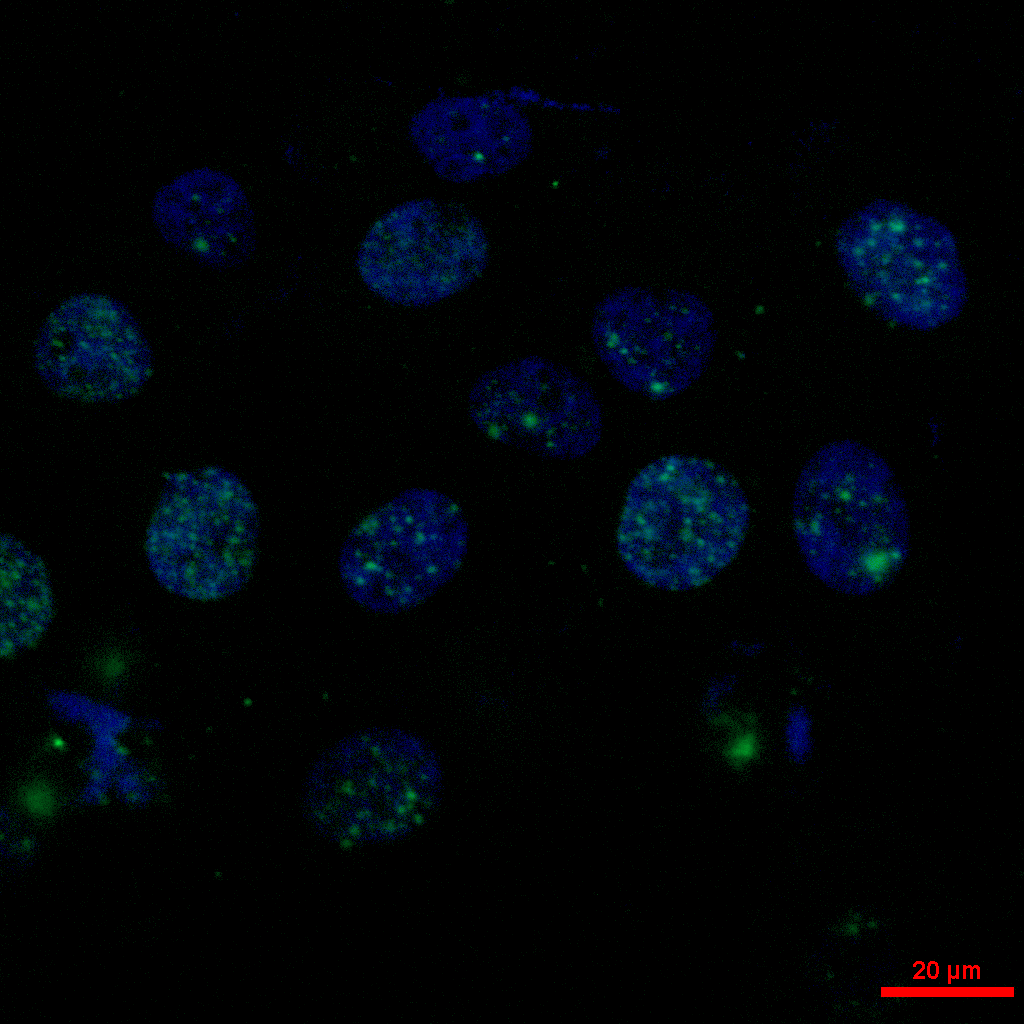

Supplement: Supplementary file 1 [file DataSheet1.zip › supplementary experimental data/3.p-H2AX/KJ-5/2-Merge.tif]

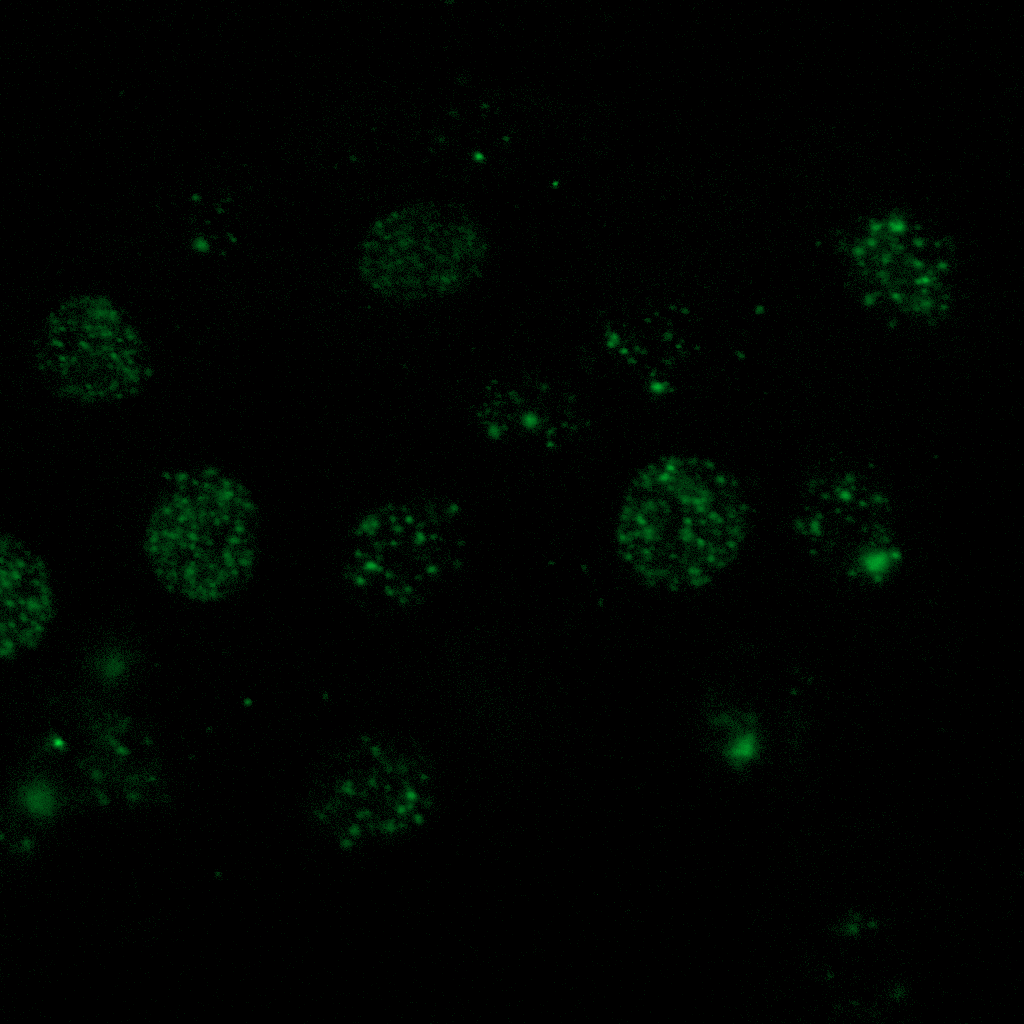

Supplement: Supplementary file 1 [file DataSheet1.zip › supplementary experimental data/3.p-H2AX/KJ-5/2-p-H2AX.tif]

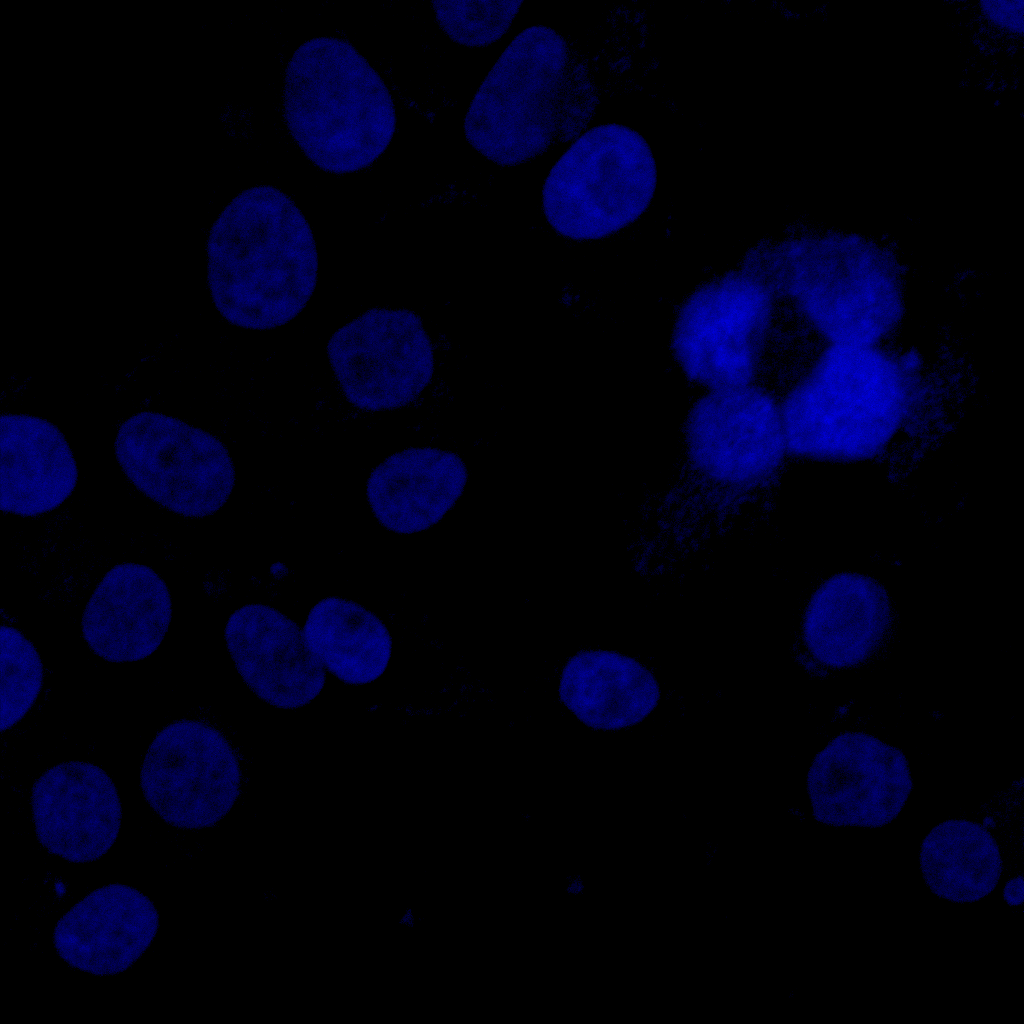

Supplement: Supplementary file 1 [file DataSheet1.zip › supplementary experimental data/3.p-H2AX/KJ-5/4-DAPI.tif]

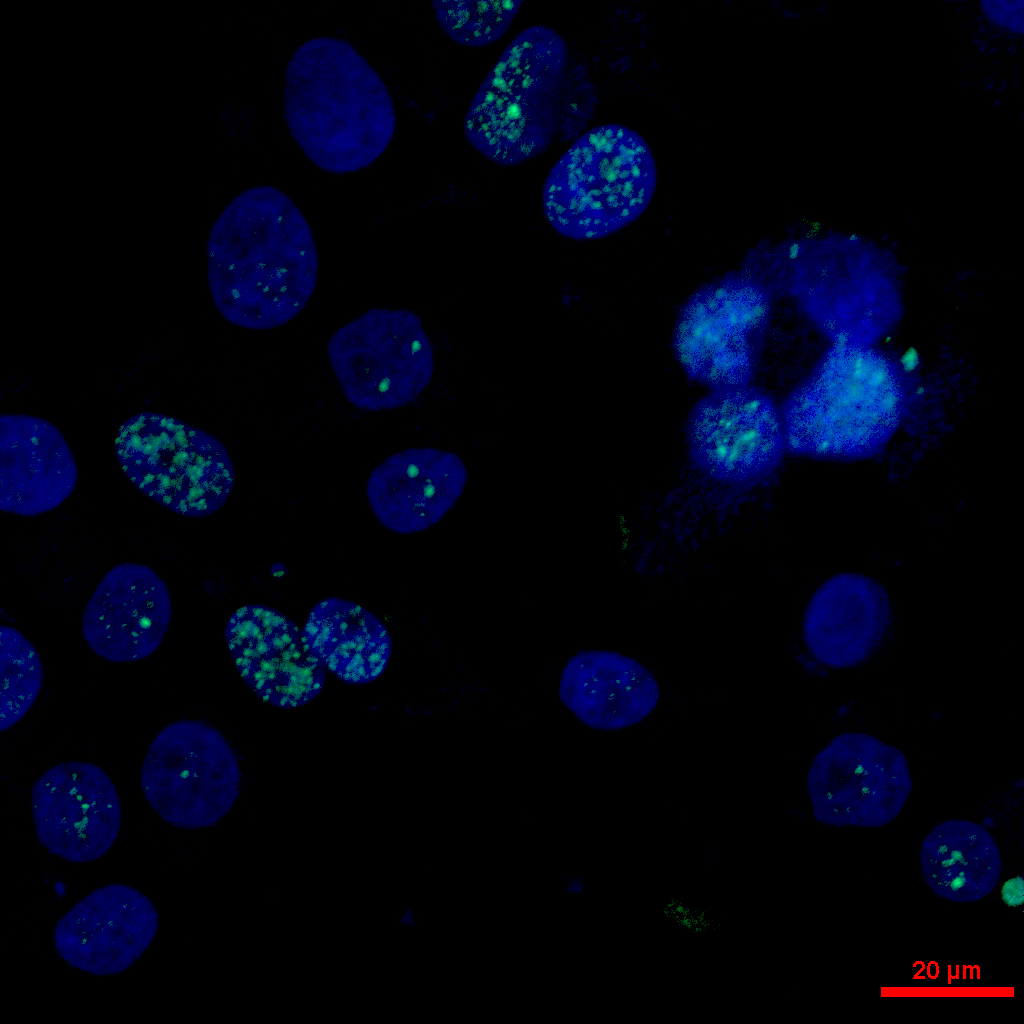

Supplement: Supplementary file 1 [file DataSheet1.zip › supplementary experimental data/3.p-H2AX/KJ-5/4-Merge.tif]

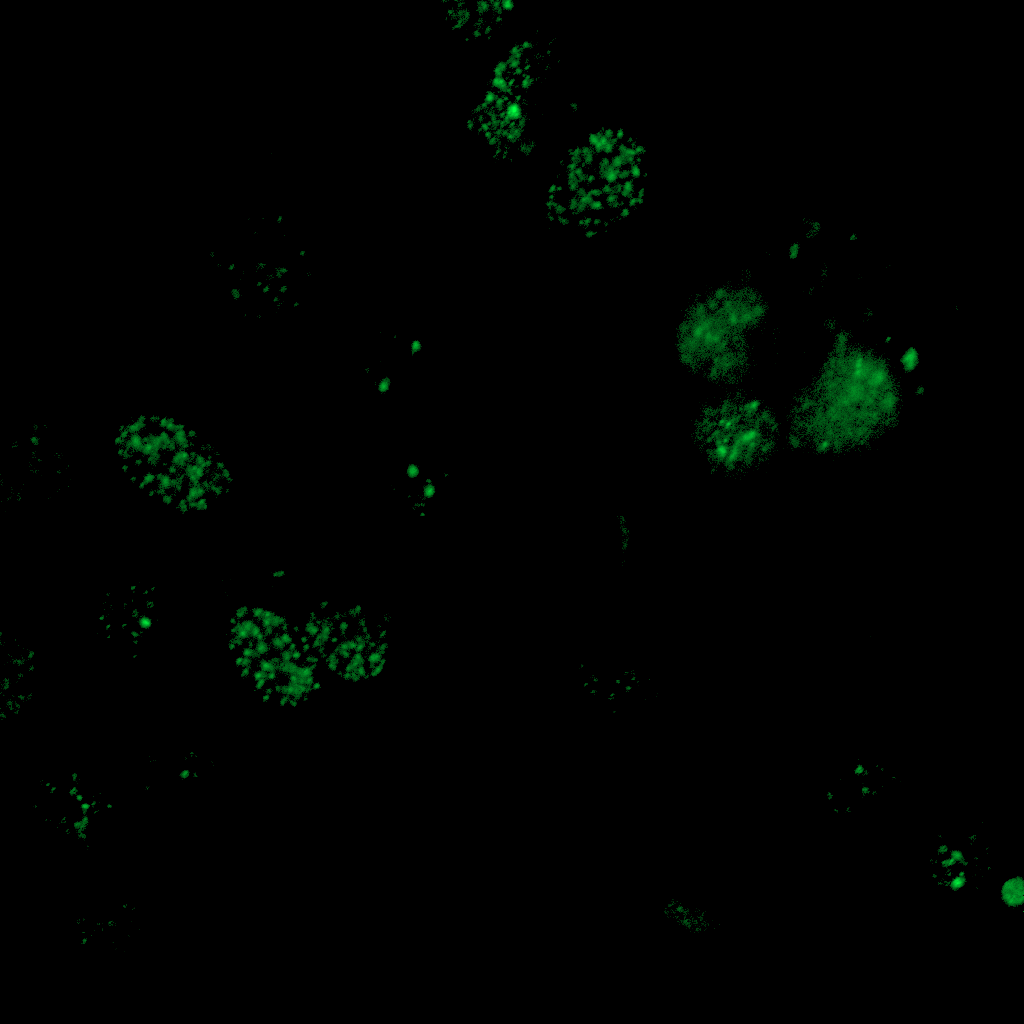

Supplement: Supplementary file 1 [file DataSheet1.zip › supplementary experimental data/3.p-H2AX/KJ-5/4-p-H2AX.tif]

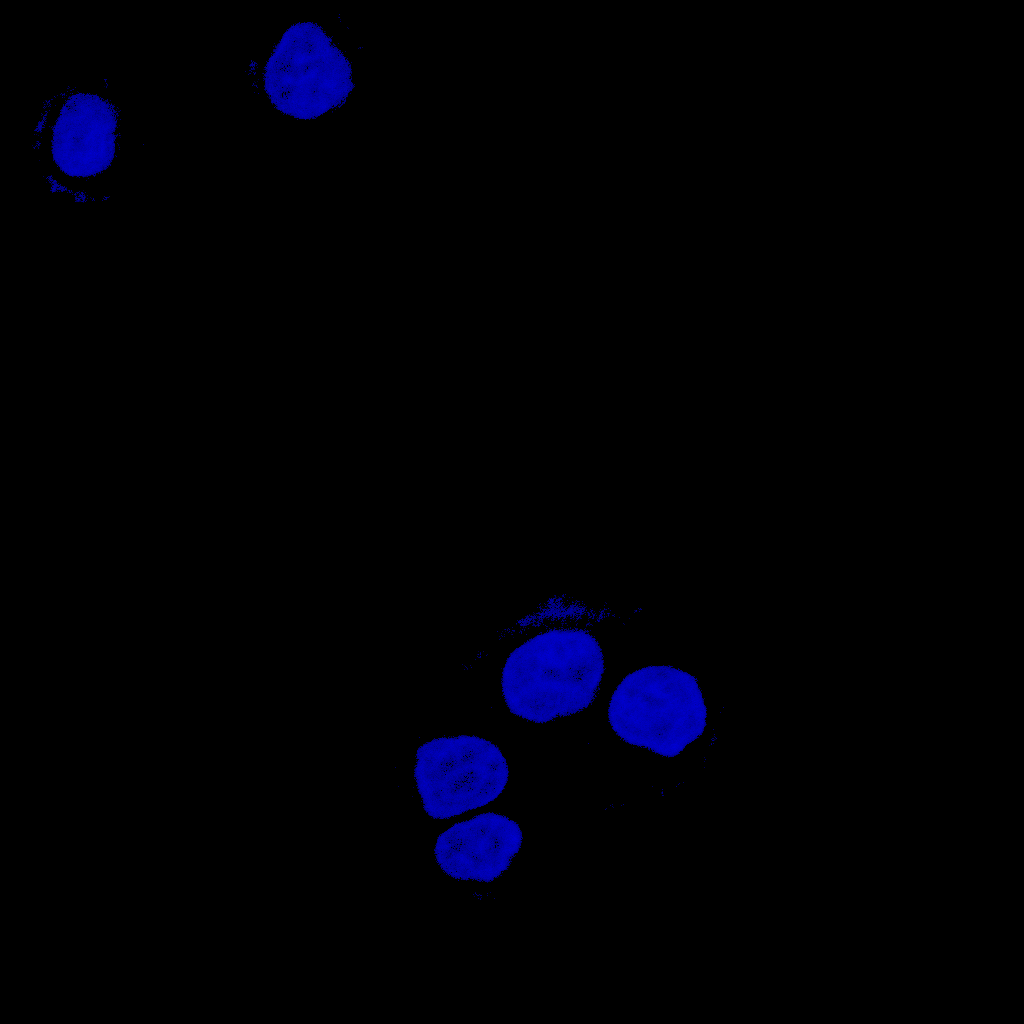

Supplement: Supplementary file 1 [file DataSheet1.zip › supplementary experimental data/3.p-H2AX/KJ-5/8-DAPI.tif]

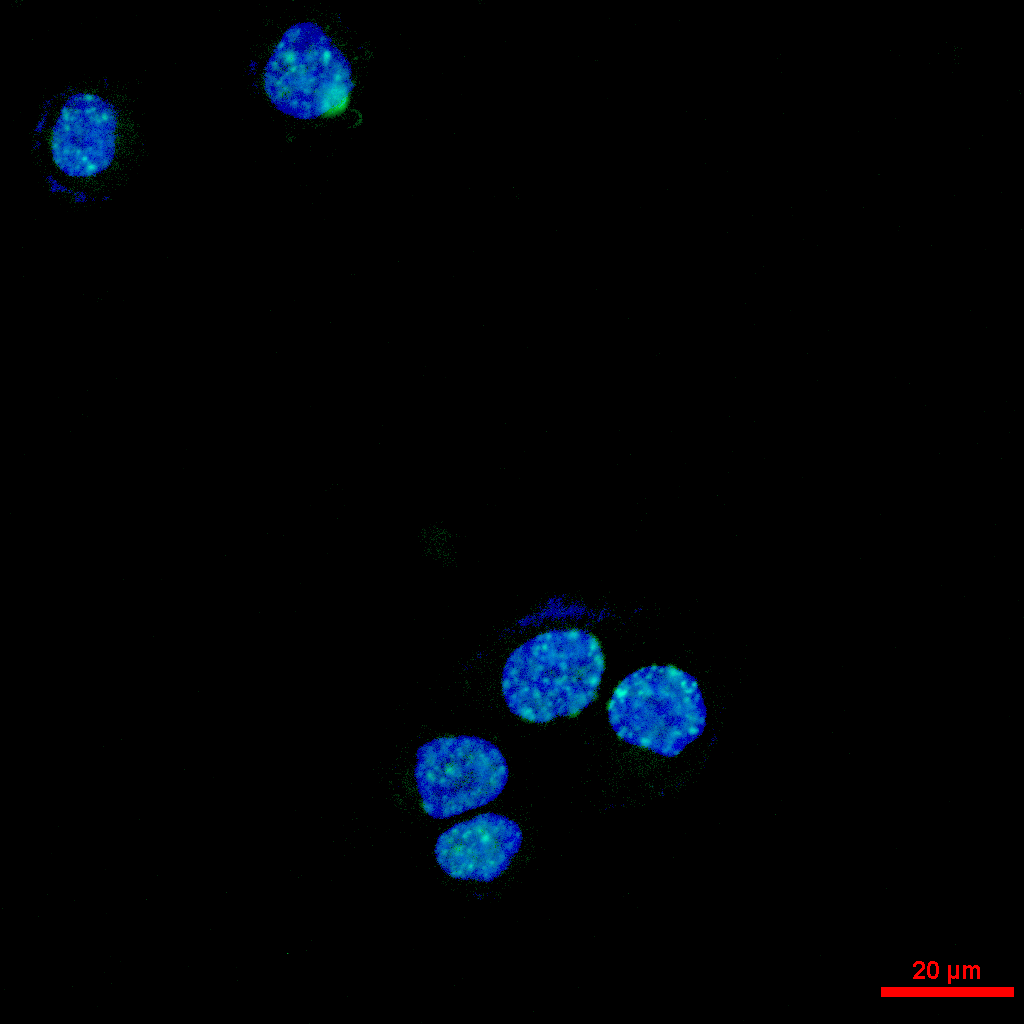

Supplement: Supplementary file 1 [file DataSheet1.zip › supplementary experimental data/3.p-H2AX/KJ-5/8-Merge.tif]

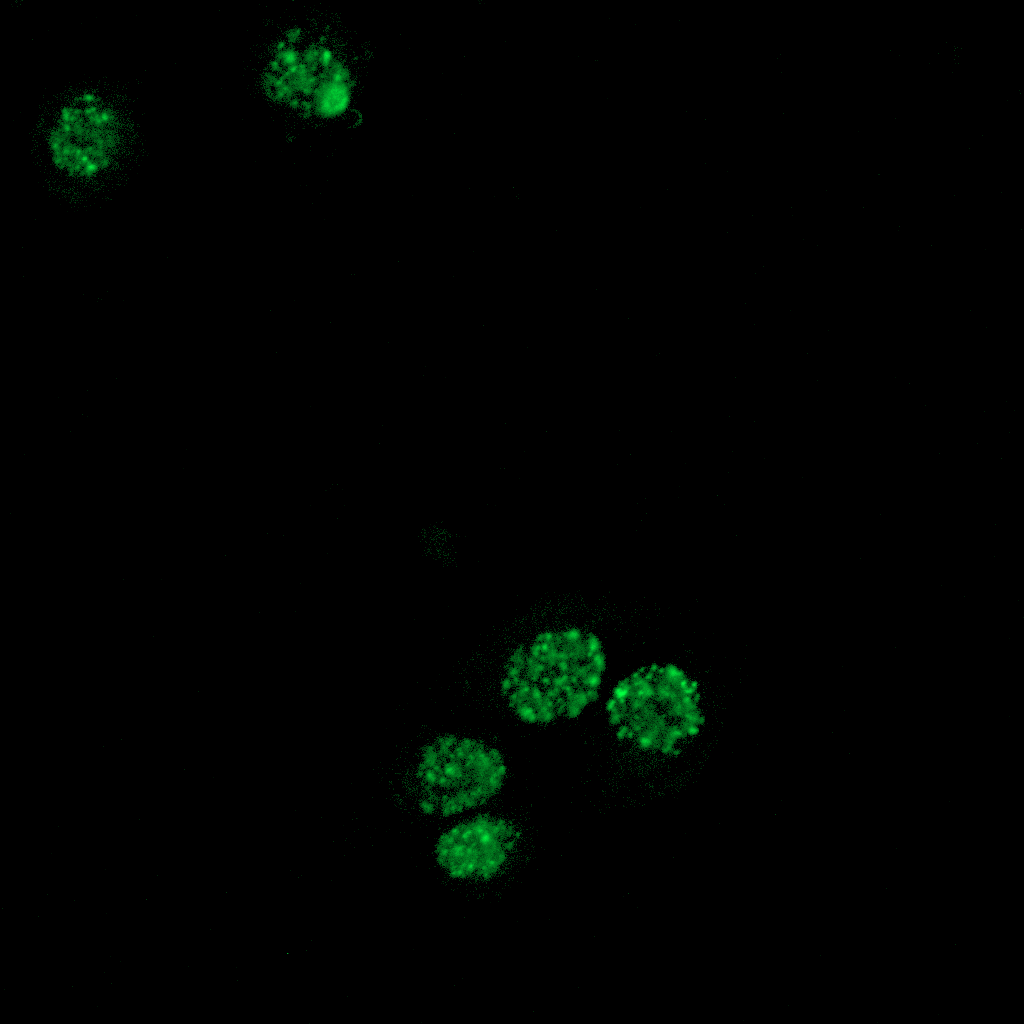

Supplement: Supplementary file 1 [file DataSheet1.zip › supplementary experimental data/3.p-H2AX/KJ-5/8-p-H2AX.tif]

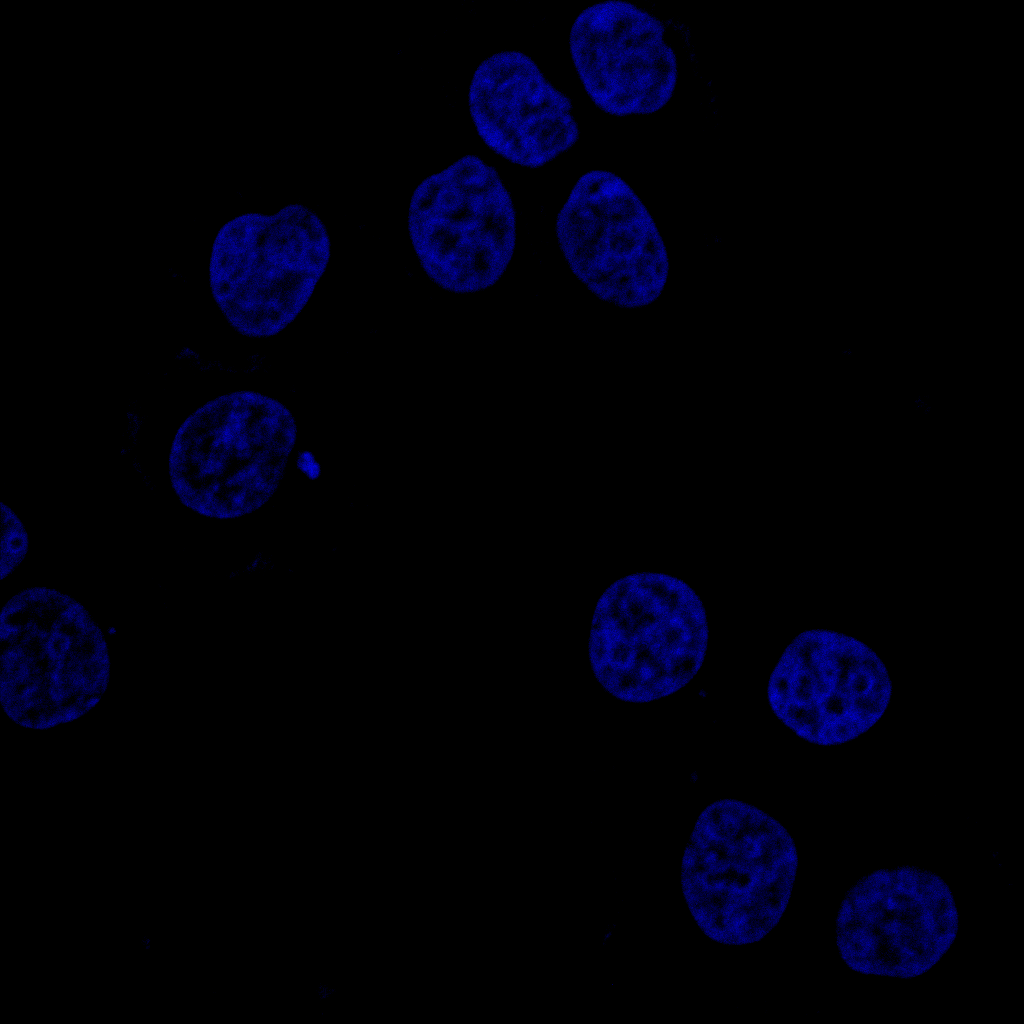

Supplement: Supplementary file 1 [file DataSheet1.zip › supplementary experimental data/3.p-H2AX/KJ-5/C-DAPI.tif]

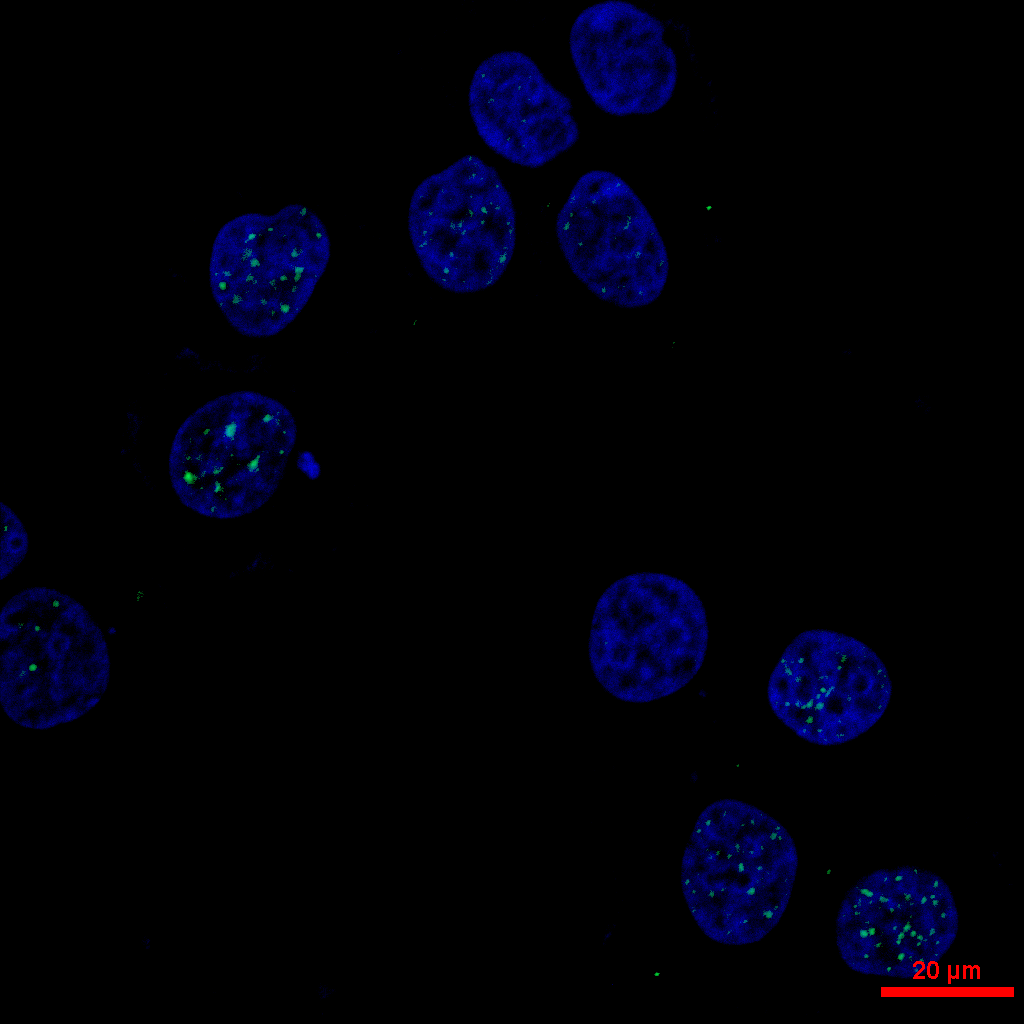

Supplement: Supplementary file 1 [file DataSheet1.zip › supplementary experimental data/3.p-H2AX/KJ-5/C-Merge.tif]

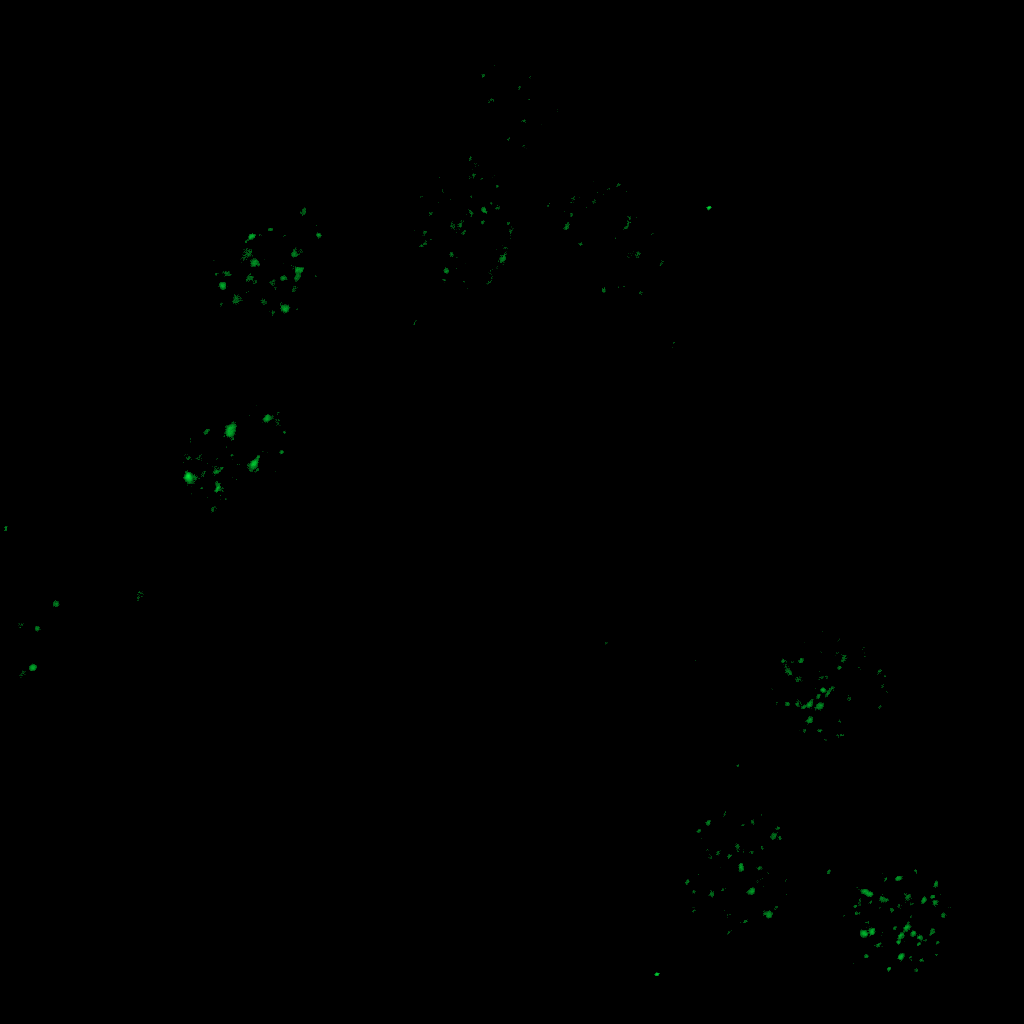

Supplement: Supplementary file 1 [file DataSheet1.zip › supplementary experimental data/3.p-H2AX/KJ-5/C-p-H2AX.tif]

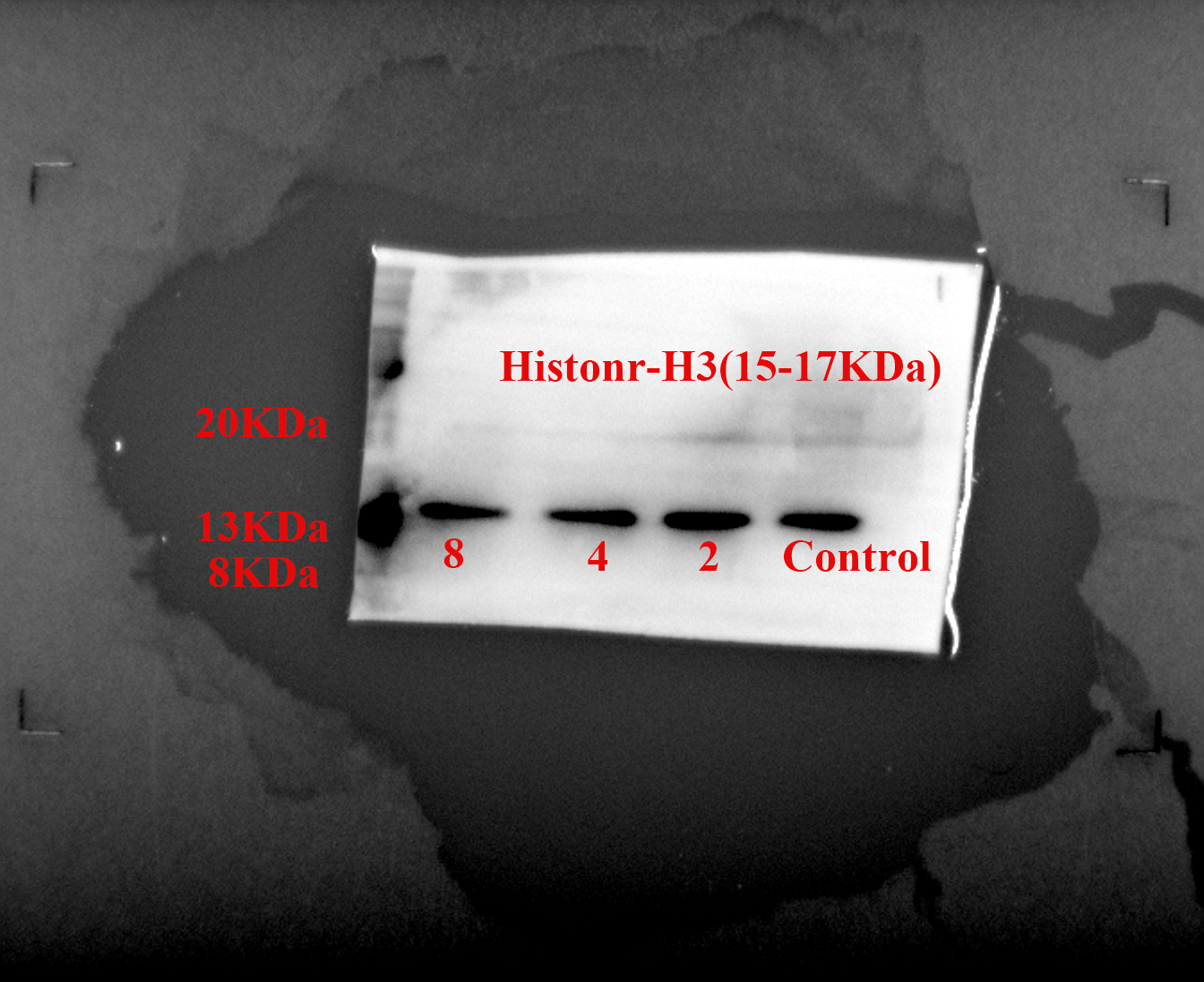

Supplement: Supplementary file 1 [file DataSheet1.zip › supplementary experimental data/3.p-H2AX/KJ-5/Histone-1 - ╕▒▒╛.tif]

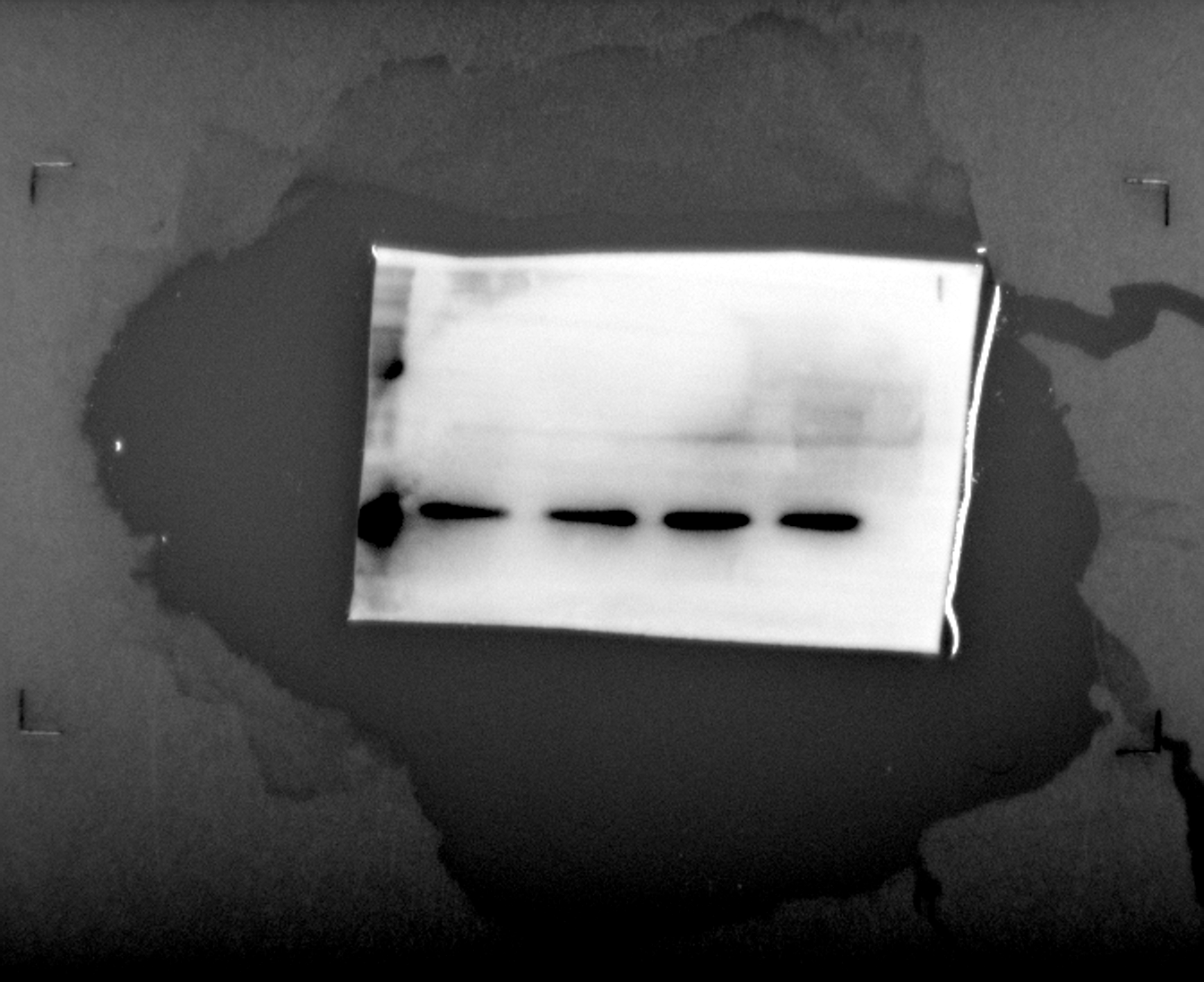

Supplement: Supplementary file 1 [file DataSheet1.zip › supplementary experimental data/3.p-H2AX/KJ-5/Histone-1.tif]

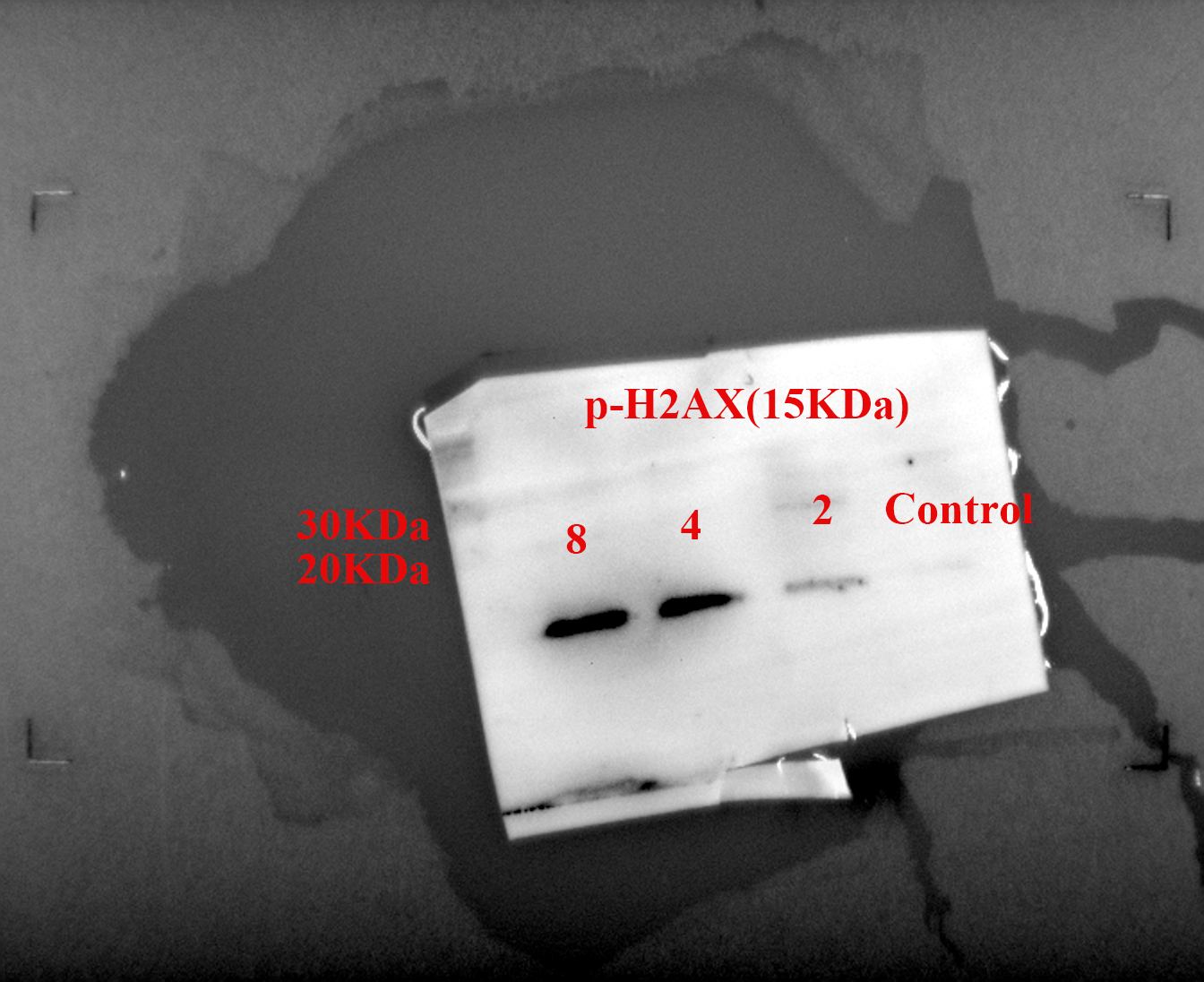

Supplement: Supplementary file 1 [file DataSheet1.zip › supplementary experimental data/3.p-H2AX/KJ-5/p-H2AX-2-3 - ╕▒▒╛.tif]

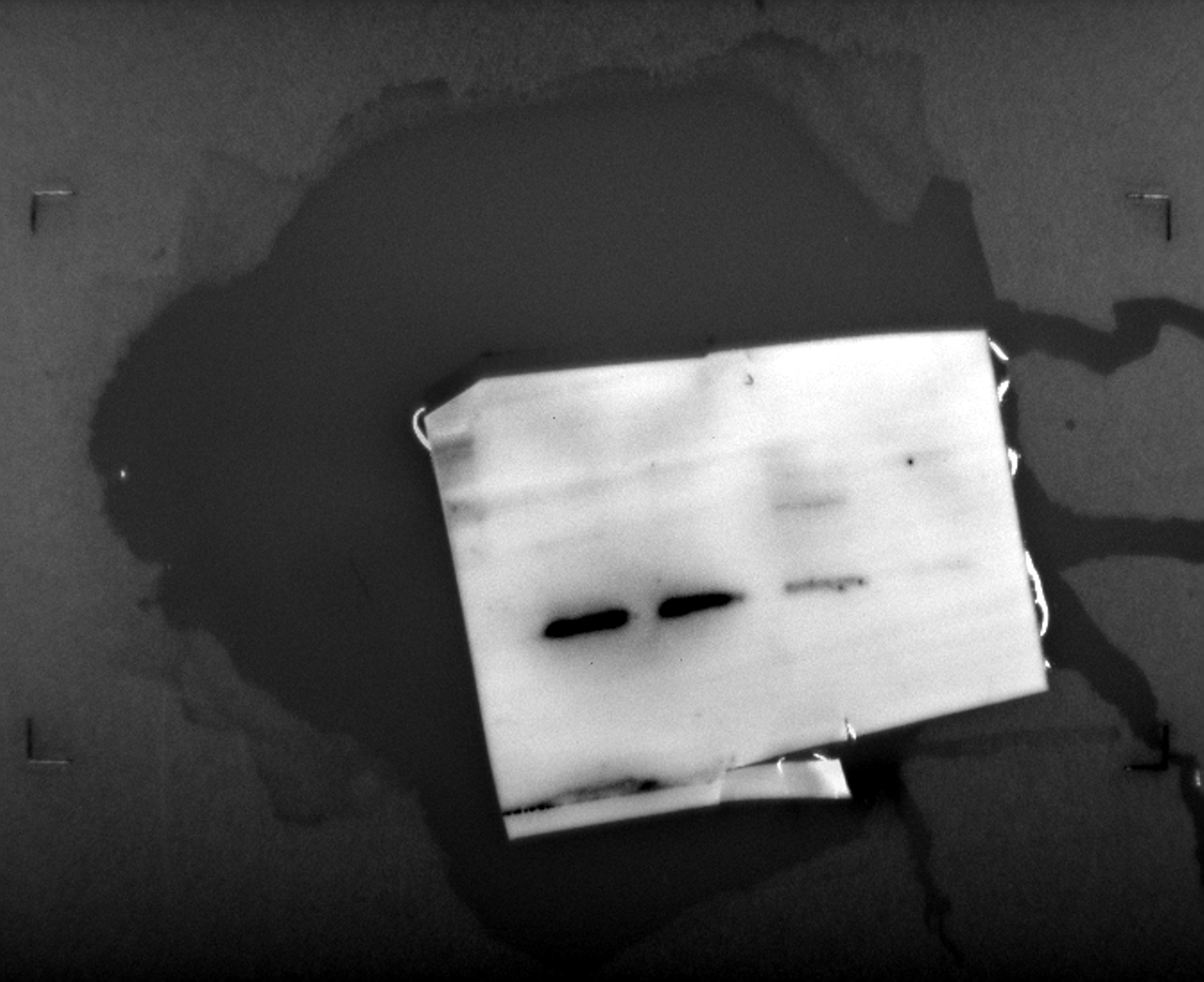

Supplement: Supplementary file 1 [file DataSheet1.zip › supplementary experimental data/3.p-H2AX/KJ-5/p-H2AX-2-3.tif]
